# Detection and sequencing of Ap_2_N-capped RNAs in human cells

**DOI:** 10.1073/pnas.2608168123

**Published:** 2026-09-15

**Authors:** Pavel Vopalensky, Ondřej Nešuta, Maria-Bianca Mititelu, Anton Škríba, Ambra Spampinato, Klára Viktorinová, Zuzana Buchová, Jana Březinová, Paul E. Reyes-Gutierrez, Ondřej Lukšan, Hana Cahova

**Affiliations:** ^a^ Institute of Organic Chemistry and Biochemistry of the Czech Academy of Sciences, Prague 6 16000, Czechia; ^b^ Department of Cell Biology, Faculty of Science, Charles University, Prague 2 12800, Czechia

**Keywords:** RNA modification, RNA cap, tRNA, dinucleoside diphosphates, Ap_2_N-RNA Seq

## Abstract

It was long believed that the eukaryotic RNA was primarily capped with 7-methylguanosine (m^7^G). However, the expanding field of noncanonical RNA caps recently revealed the presence of other structures (nicotinamide adenine dinucleotide, flavin adenine dinucleotide, dinucleoside polyphosphates) at the 5′ end of various mRNAs and noncoding RNAs. We report here on the detection and quantification of a new type of RNA cap—diadenosine diphosphate (Ap_2_A) and adenosine guanosine diphosphate (Ap_2_G)—on mammalian RNA. We have established a liquid chromatography–mass spectrometry (LC-MS) method to measure Ap_2_N-RNA levels and observed its increase under oxidative stress. To identify Ap_2_N-capped RNAs, we have developed a sequencing method (Ap_2_N-RNA Seq) by leveraging the ability of truncated T4 RNA Ligase 2 to selectively ligate Ap_2_N-capped RNAs to an RNA adaptor. We applied this method to human short RNA and identified a subset of tRNA-derived species as candidate Ap_2_N-capped RNAs. Among these, Ap_2_N-RNA Seq revealed previously uncharacterized 3′ tRNA fragments consistent with Ap_2_A capping at their 5′ ends. This expands current understanding of tRNA capping and processing and broadens the toolkit for studying the RNA life cycle.

## Significance

Apart from the canonical eukaryotic mRNA cap, an increasing number of noncanonical caps (nicotinamide adenine dinucleotide, dinucleotide polyphosphates) have been discovered on other RNA types in both prokaryotes and eukaryotes. Here, using liquid chromatography–mass spectrometry, we found the dinucleoside diphosphate (Ap_2_N) serving as 5′ RNA cap in human cells. To gain insights into the Ap_2_N capping, we developed a specific Ap_2_N-RNA sequencing protocol and applied it on short RNA. The most prominent Ap_2_N-RNA-seq candidates corresponded to tRNAs and previously unknown tRNA-derived fragments. Our work suggests that the Ap_2_N cap may be formed as an intermediate in a RNA-processing pathway and provides tools to investigate the roles of Ap_2_N-capped RNAs in cellular physiology.

Dinucleoside polyphosphates (Np*_n_*Ns), particularly diadenosine triphosphate (Ap_3_A) and diadenosine tetraphosphate (Ap_4_A), have been reported to accumulate in response to diverse stress conditions and have therefore been discussed as “alarmones” (1–4). In addition to potentially being synthesized through other biosynthetic pathways (5), Np*_n_*Ns are formed by certain aminoacyl-tRNA synthetases (6, 7). They can be degraded by NudiX enzymes, a widely distributed family of hydrolases present in all domains of life (8). Generally, NudiX hydrolases cleave nucleoside diphosphates (hence “Nudi”) linked to some moiety (denoted by “X”). Although the role of extracellular Np*_n_*Ns as agonists of purinergic receptors (9) is well understood, their intracellular function remains a subject of ongoing studies.

It has been discovered that Np_*n*_Ns play the role of 5′ RNA caps in bacteria (10, 11) and that the concentration of capped RNA depends on the environment inside the bacterial cell, which changes with the phase of growth or the level of stress. The most probable mechanism of Np_*n*_N-RNA formation is direct incorporation of Np_*n*_Ns into RNA by RNA polymerases (12–15). Recently we have reported that also mammalian cells contain RNA capped with diadenosine tetraphosphate (Ap_4_A) (16), one of the most common Np*_n_*Ns, which has been discovered to play an important role, for example, during mast cell activation (17). In our search for new noncanonical RNA caps and their potential association with intracellular stress, we have also examined other types of Np*_n_*N-RNA, for example dinucleoside diphosphates (Np_2_Ns), which represent the least explored class of Np*_n_*Ns. Examples of Np_2_Ns include diadenosine diphosphate (Ap_2_A), adenosine guanosine diphosphate (Ap_2_G), and diguanosine diphosphate (Gp_2_G), which have been detected in human myocardial tissue (18), in adrenal glands (19), and in releasable granules of human platelets where they were reported to act as endogenous modulators of platelet aggregation (20, 21). Ap_2_N-RNAs have been proposed to exist as intermediates produced, for example, during ligation reactions (22, 23), but they have never been detected in vivo.

Here, we report the detection of Ap_2_N-RNA in a human cell line (Expi293F), employing liquid chromatography–mass spectrometry (LC–MS). We have also developed a method for the selective sequencing of RNA bearing this chemical structure and refer to it as Ap_2_N-RNA Seq. By applying this method to RNA isolated from Expi293F cells, we identified subsets of tRNA-derived RNAs, including previously uncharacterized 3′ tRNA fragments, as candidate Ap_2_N-capped species.

## Results

### Detection of Ap_2_N RNA Cap by LC–MS.

**Fig. 1.**
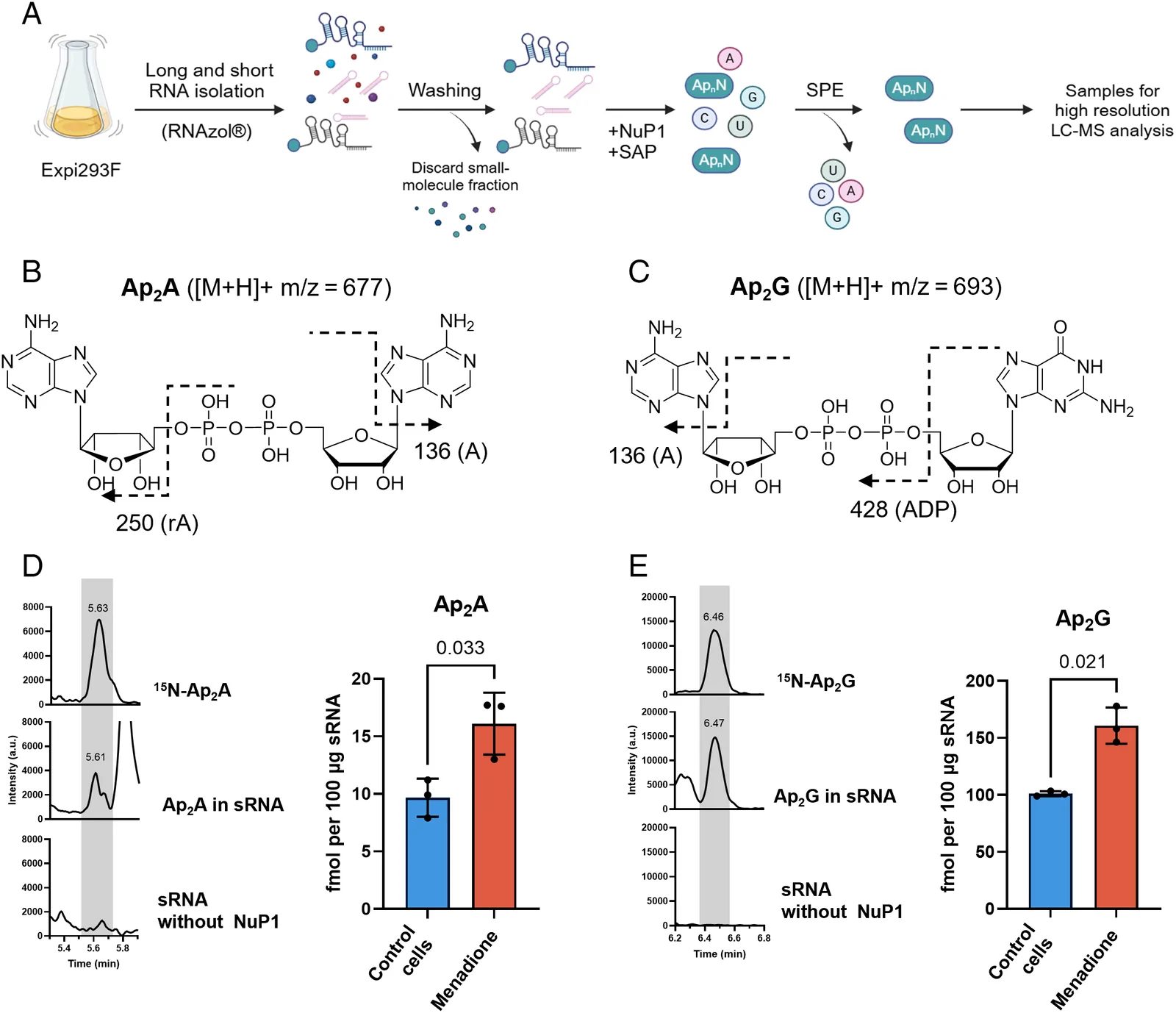
Detection of Ap_2_A and Ap_2_G RNA caps by LC–MS. (*A*) Experimental design of RNA isolation and digestion with subsequent LC–MS analysis. (*B* and *C*) Chemical structures of Ap_2_A and Ap_2_G formed after RNA digestion. The arrows depict the fragment ions used for detection with the MRM method. (*D*) MRM chromatograms showing the spiked ^15^N-Ap_2_A standard (MRM transition 687 → 255), Ap_2_A (MRM transition 677 → 250) in Expi293F sRNA after digestion with NuP1 and SAP, and sRNA treated without NuP1 (negative control). Ap_2_A-RNA quantification in sRNA, isolated from control and menadione-treated Expi293F cells. (*E*) MRM chromatograms showing the spiked ^15^N-Ap_2_G standard (MRM transition 698 → 433), Ap_2_G (MRM transition 693 → 428) in Expi293F sRNA after digestion with NuP1 and SAP, and sRNA treated without NuP1 (negative control). Ap_2_G-RNA quantification in sRNA, isolated from control and menadione-treated Expi293F cells. Data are shown as mean ± SD from three biological replicates. The level of significance was calculated using the paired *t* test and expressed as *P*-value.

To assess the presence of Ap_2_N-capped RNAs, we isolated short- and long-RNA fractions (sRNA and lRNA, respectively) from Expi293F cells. We then washed the RNA to remove noncovalently bound molecules and digested it by nuclease P1 (NuP1) and shrimp alkaline phosphatase (SAP) into free nucleosides and intact RNA caps (Fig. 1*A*). Subsequently, the samples were purified by solid phase extraction (SPE) to wash out free nucleosides and thus improve the signal-to-noise ratio in the following LC–MS analysis. Using a triple quadrupole mass spectrometer, we detected the Ap_2_A and Ap_2_G molecules originating from the digested sRNA. The amounts of Ap_2_A and Ap_2_G in the lRNA fraction were at or near the limit of detection and were not reproducible across biological replicates; we therefore focused subsequent analyses only on the sRNA fraction. The presence of the Ap_2_A and Ap_2_G molecules was confirmed by identical retention time and corresponding multiple reaction monitoring (MRM) transitions with N^15^ labeled Ap_2_A and Ap_2_G standard spikes (Fig. 1 and *SI Appendix*, Figs. S1 and S2). Ap_2_A was detected as protonated ion [M+H]^+^ with m/z = 677.1 fragmented into ions with m/z = 250.0 and m/z = 136.0, corresponding to adenosine (rA) and adenine (A) fragments, respectively (Fig. 1*B* and *SI Appendix*, Tables S1–S4), while Ap_2_G as [M+H]^+^ m/z = 693.1 fragmented into masses of [M+H]^+^ m/z = 428.0 and [M+H]^+^ m/z = 136.0 corresponding to adenosine diphosphate (ADP) and adenine (A) fragments (Fig. 1*C*). RNA treated in the absence of NuP1 did not contain ions mentioned above, indicating that the observed Ap_2_A and Ap_2_G in NuP1-treated samples were originally covalently bound to isolated RNA (Fig. 1 *D* and *E*). To further confirm that observed masses correspond to expected Ap_2_N caps, we also analyzed putative structures with the same mass such as ApAp, pApA, and Ap_2_G (3′ to 5′). None of them had the same retention as Ap_2_A or Ap_2_G (*SI Appendix*, Fig. S3).

The amount of Np*_n_*Ns (such as Ap_3_A and Ap_4_A) was previously reported to increase with the intracellular response to stress (4, 24). We therefore examined the effects of different stress conditions on Ap_2_A-RNA and Ap_2_G-RNA levels. Paraquat (25) and menadione (26) were used as mainly oxidative stress inducers and validated by GSH/GSSG assays, whereas bleomycin-induced DNA damage (27) was confirmed by detection of phosphorylated H2AX (γH2AX) by western blot (*SI Appendix*, Fig. S4). Paraquat treatment did not produce substantial changes in Ap_2_A-RNA nor Ap_2_G-RNA, whereas bleomycin induced a limited increase only in Ap_2_G-RNA (*SI Appendix*, Fig. S5). In contrast, menadione treatment robustly increased both RNA caps in the sRNA fraction (Fig. 1 *D* and *E*) and was therefore selected for subsequent analyses. The measured amount of Ap_2_A-RNA was 9.7 ± 1.7 fmol per 100 μg of sRNA for control cells and 16.1 ± 2.7 fmol per 100 μg of sRNA for menadione-treated cells and the measured amount of Ap_2_G-RNA was 101.1 ± 2.2 fmol per 100 μg of sRNA for control cells and 160.8 ± 15.9 fmol per 100 μg of sRNA and for menadione-treated cells, respectively. These data indicate that sRNA fraction contains Ap_2_A- and Ap_2_G-RNA and its levels increase under menadione induced stress.

### Formation and Degradation of Ap_2_N-RNA.

**Fig. 2.**
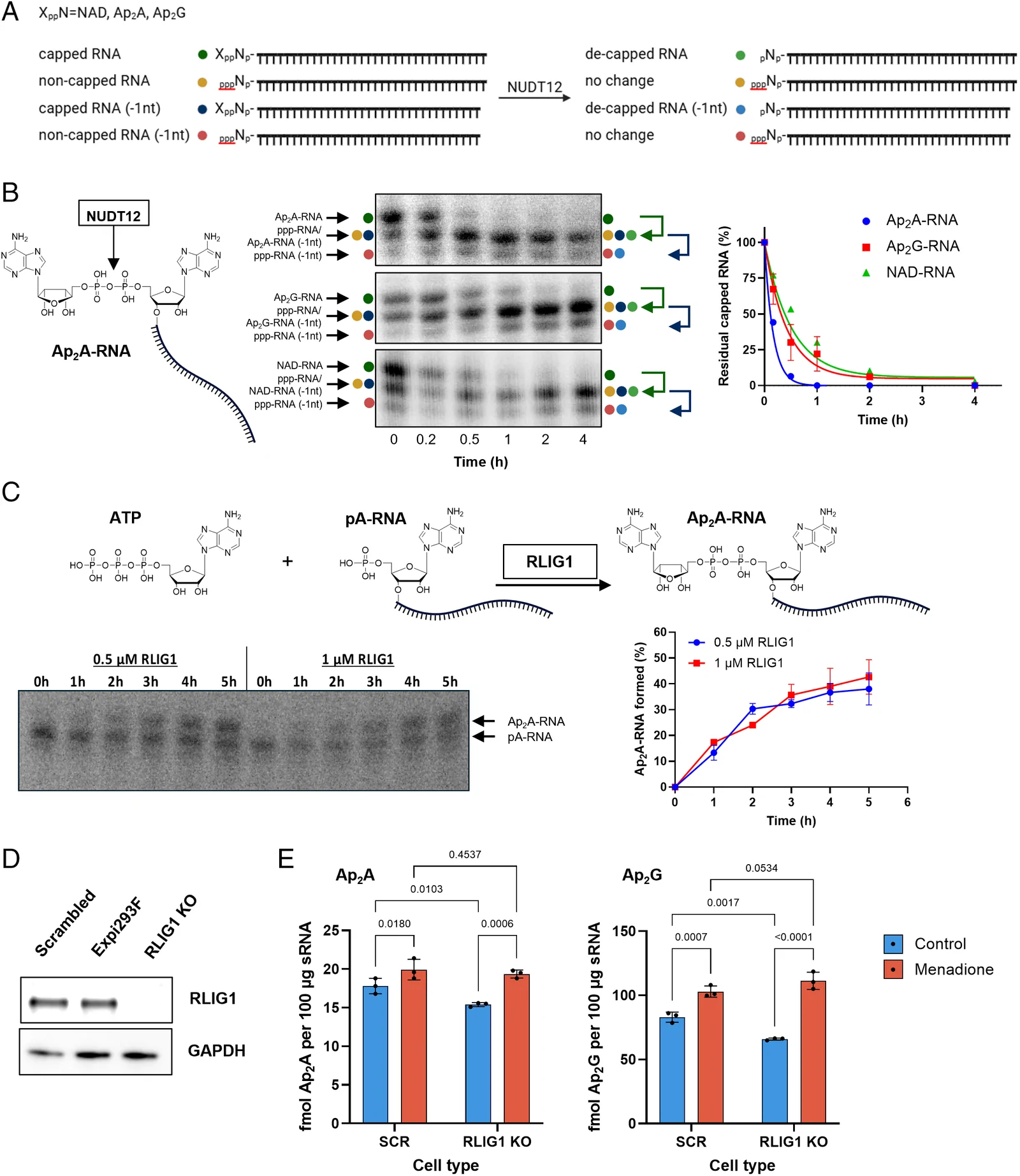
Degradation and formation of Ap_2_N-RNA. (*A*) Scheme of various RNA entities (as products of T7 RNA polymerase) present in the reaction mixture and their changes after NUDT12 treatment. Their color labels match with the PAGE labels in panel *B* in order to clarify bands interpretation. (*B*) Chemical structures of Ap_2_A-RNA and position of Nudt12 cleavage indicated by arrow. Examples of PAGE analysis of ^32^P-radiolabeled Ap_2_A-, Ap_2_G-, and NAD-RNA after incubation with NUDT12 (1 µM) in time-dependent manner (0 to 4 h of incubation). Quantification of RNA decapping activity of NUDT12 on NAD-, Ap_2_A-, and Ap_2_G-RNA as substrates. Data are shown as mean ± SD from experiments performed in triplicates. (*C*) Formation of Ap_2_A-RNA by enzymatic activity of human RLIG1 in vitro. Example of ^32^P-radiolabeled p-RNA capping by RLIG1 in time-dependent manner (0 to 5 h) and its quantification of three independent replicates. (*D*) Representative western blot analysis of the scrambled (SCR), unmodified (Expi293F), and RLIG1 KO Expi293F cell lines showing protein levels of RLIG1. GAPDH was used as a loading control. (*E*) LC–MS quantification of the levels of Ap_2_A- and Ap_2_G-RNA in sRNA fraction isolated from the SCR and RLIG1 KO cell lines. Data are shown as mean ± SD from three biological replicates. The level of significance was calculated using the paired *t* test and expressed as *P*-value.

Because the steady-state levels of Ap_2_N-RNA molecules may be influenced by the rate of cellular formation and degradation, we examined enzymes potentially involved in both processes. Ap_2_N caps have a very similar chemical structure to NAD (*SI Appendix*, Fig. S6), the decapping of which has been extensively studied (28–33). Therefore, we tested whether typical human NAD-RNA decapping enzymes, such as DXO (30) and NUDT12 (32), can also cleave Ap_2_A-RNA and Ap_2_G-RNA. We prepared radioactively labeled Ap_2_A-, Ap_2_G-, and NAD-RNA by in vitro transcription and incubated it with both enzymes (Fig. 2 *A* and *B* and *SI Appendix*, Fig. S6). We observed a similar cleavage pattern with all the Ap_2_A, Ap_2_G, and NAD RNA caps: NUDT12 cleaves the pyrophosphate bond within the cap whereas DXO cleaves off the entire cap structure, leaving the RNA one nucleotide shorter (Fig. 2*A* and *SI Appendix*, Fig. S6). In the time dependence cleavage experiment, surprisingly, Ap_2_A-RNA showed the highest cleavage rate by both enzymes (Fig. 2*B* and *SI Appendix*, Fig. S6).

To understand whether the increase in Ap_2_N-RNA following menadione stress was related to altered expression levels of these decapping enzymes DXO and NUDT12, we performed a differential RNA-seq analysis of control and menadione-stressed cells (*SI Appendix*, Fig. S7 and Dataset S1). Unexpectedly, we found that the genes for NUDT12 and DXO were slightly upregulated under the stress conditions (*SI Appendix*, Fig. S7). Among the other Nudix family genes, only NUDT15 and NUDT21 showed significant expression changes (*SI Appendix*, Fig. S7 and Dataset S1) on the mRNA level. However, these enzymes have not been implicated in 5′-end RNA processing. Subsequent RT-qPCR analysis showed comparable expression levels of NUDT12 and DXO mRNA between control and stressed cells (*SI Appendix*, Fig. S7 and Table S5). Because both RNA-seq and RT–qPCR measure mRNA abundance and do not account for protein levels, which better reflects the enzymatic activity, we next performed a proteomic analysis of control and menadione-stressed cells (*SI Appendix*, Fig. S8). Although we observed down-regulation of some specific genes in menadione-stressed cells, we did not observe any significant change in DXO, NUDT12 nor any other NudiX enzymes (Dataset S2). One protein was upregulated under stress conditions, while five were downregulated; however, none of these proteins have been previously implicated in RNA processing. We additionally examined NUDT12 and DXO by western blotting (*SI Appendix*, Fig. S8). Menadione treatment was associated with a modest reduction in western blot signal for both enzymes; however, this trend was variable and was not supported by significant changes in the corresponding bottom–up proteomics data. Therefore, we do not interpret the western blot result as evidence for reduced total protein abundance. Instead, the discrepancy may reflect methodological differences between antibody-based detection and peptide-based proteomics, including possible effects of protein processing, degradation, or stress-induced posttranslational modifications on antibody recognition (34). Collectively, these data indicate that menadione treatment does not cause a robust detectable decrease in total NUDT12, DXO, or Nudix enzyme abundance under our experimental conditions. The increased Ap_2_N-RNA levels observed after 3.5 h of menadione treatment may therefore involve regulation at other levels or contributions from additional, as-yet-unidentified factors.

Therefore, we examined the mechanism underlying Ap_2_N-RNA formation. To date, Np*_n_*N-capped RNAs have been attributed to the direct incorporation of free Np*_n_*Ns into nascent RNA by RNA polymerase as NCINs (10, 12, 13). This mechanism requires the presence of free Np*_n_*Ns in the nucleus. Because the reports of Ap_2_A existing in free form are rather limited to a few specific tissues, we extracted the fraction of small molecules from Expi293F cells and analyzed it using LC–MS. We detected Ap_2_A, and its signal increased upon menadione treatment, whereas Ap_2_G was not detected (*SI Appendix*, Fig. S9). Although free Ap_2_A could, in principle, be incorporated directly into RNA as a NCIN, the absence of detectable free Ap_2_G led us to consider an alternative route for Ap_2_N–RNA formation. We therefore investigated a putative RNA-capping enzyme–mediated mechanism. Based on theory that RNA capping enzymes and RNA ligases may belong to the same superfamily (35–37), we hypothesized that an RNA ligase might be responsible for the formation of Ap_2_N-capped RNA. Recently, two works have independently described and characterized a new type of human RNA ligase (RLIG1) (22, 23) that can specifically ligate 5′ phosphate-RNA (5′ p-RNA) to 3′ OH-RNA. This process requires the formation of 5′ adenosine phosphoanhydride intermediate RNA, which has, in principle, the same chemical structure as Ap_2_N-RNA. We therefore purified recombinant human RLIG1 from *Escherichia coli* and tested whether this enzyme could convert 5′ p-RNA into Ap_2_N-RNA. We prepared 5′ p-RNA starting either with adenosine monophosphate (5′ pA-RNA) or guanosine monophosphate (5′ pG-RNA) by in vitro transcription and subsequent 5′ polyphosphatase treatment. These RNAs were incubated with or without RLIG1 in the presence of ATP and analyzed either by PAGE (radioactively labeled) (Fig. 2*C* and *SI Appendix*, Fig. S10) or by LC–MS as described above. Both assays showed formation of Ap_2_A-RNA and Ap_2_G-RNA upon incubation of RLIG1 with 5′ pA-RNA or 5′ pG-RNA, respectively, whereas no Ap_2_A-RNA nor Ap_2_G-RNA was detected in the absence of RLIG1 (Fig. 2*C* and *SI Appendix*, Fig. S10). This demonstrates that RLIG1 can catalyze the formation of Ap_2_N-capped RNA in vitro.

To investigate whether RLIG1 contributes to the formation of Ap_2_N-RNA also in vivo, we first assessed its expression. Although RNA-seq suggested an increase in RLIG1 expression upon menadione treatment (Dataset S1 and *SI Appendix*, Fig. S10), this trend was not supported by RT-qPCR nor by proteomic analysis (Dataset S2 and *SI Appendix*, Figs. S8 and S10) nor by western blot analysis (*SI Appendix*, Fig. S8). To ultimately investigate the role of RLIG1 in Ap_2_N-RNA formation, we generated Expi293F cells with a knockout of RLIG1 (hereafter referred to as RLIG1 KO, Fig. 2*D* and *SI Appendix*, Fig. S11). As a control, cells expressing a scrambled sgRNA were generated using the same system (hereafter referred to as SCR). Both cell lines were subjected to menadione-induced stress, after which the short RNA fraction was isolated. We then compared the levels of Ap_2_A- and Ap_2_G-capped RNA under control conditions and upon menadione stress (Fig. 2*E*). Under control conditions, the abundance of both RNA caps was lower in RLIG1 KO cells compared to SCR cells. However, upon menadione treatment, the levels of both caps increased in both cell lines to similar extents. The absolute levels of Ap_2_A- and Ap_2_G-capped RNAs in the SCR and RLIG1 KO cell lines differ slightly from those observed in the parental Expi293F line (Fig. 1 *D* and *E*), which may reflect altered cellular physiology associated with lentivirus-based generation of the SCR and RLIG1 KO cell lines. These results suggest that RLIG1 contributes to the formation of a minor subset of Ap_2_N-capped RNAs (approximately 20%), whereas the majority of these RNA species are likely generated by other, yet unidentified mechanisms.

### Development of Ap_2_N-RNA Sequencing Method.

**Fig. 3.**
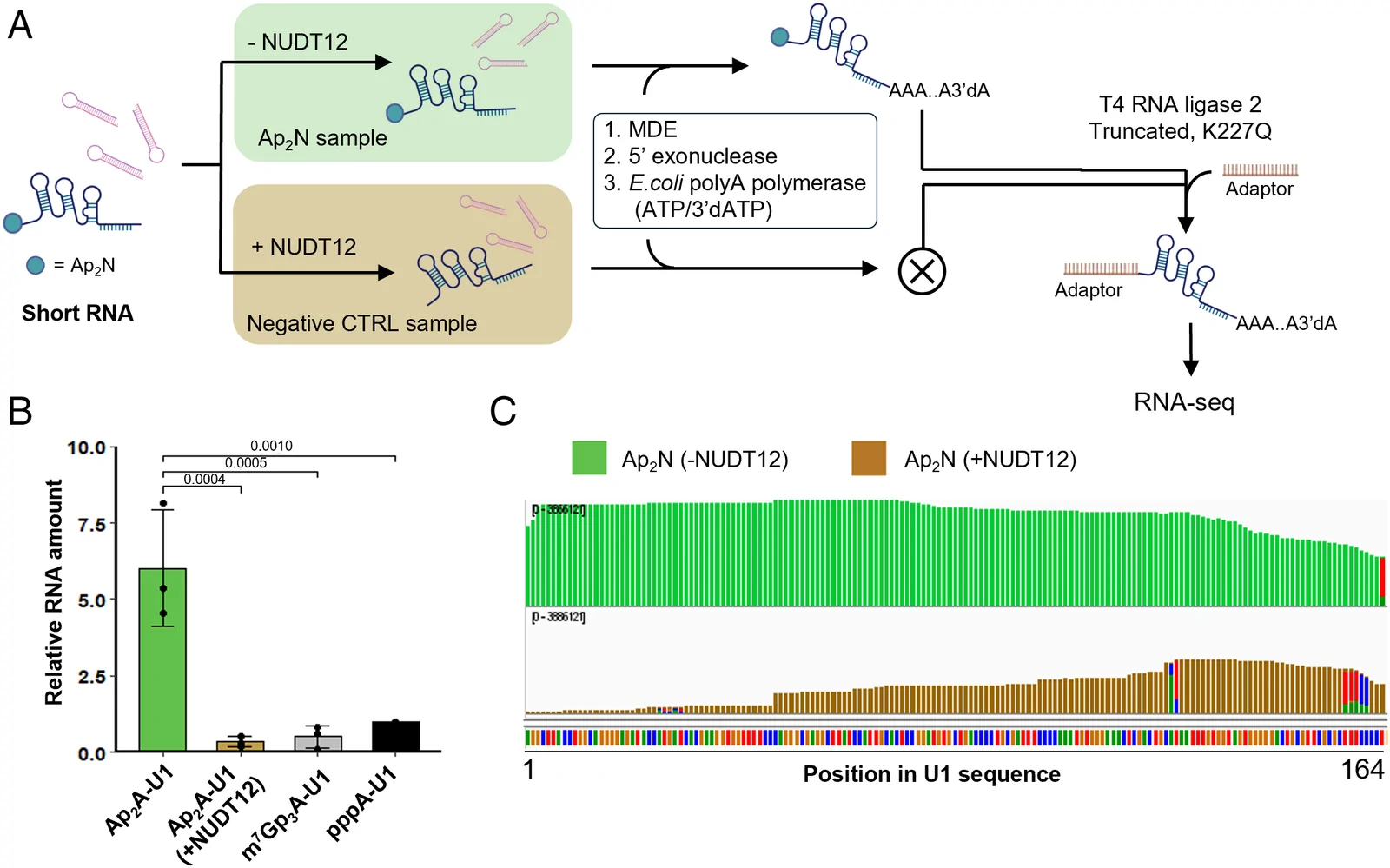
Ap_2_N-RNA Seq protocol. (*A*) Overview of the Ap_2_N-RNA Seq method. First, the isolated short RNA is either left untreated (Ap_2_N sample) or treated with NUDT12 to remove the Ap_2_N cap (Negative CTRL). Subsequently, all m^7^G capped RNAs are removed by incubation with the mRNA decapping enzyme (MDE) and 5′ exonuclease. Afterward, a short polyA tail is added by *E. coli* polyA polymerase. During this step, 70% of 3′ dATP (cordycepin) is added to the reaction to shield the 3′ end of RNA and avoid ligation and circularization in the subsequent step. Finally, an RNA adaptor is ligated to Ap_2_N-RNA by truncated T4 RNA ligase 2 K227Q, the resulting RNA is reverse transcribed using polyT oligo, amplified, and subjected to dsDNA Illumina sequencing. (*B*) RT-qPCR analysis of the products of the Ap_2_N-RNA Seq method applied to a model Ap_2_A-U1 snRNA. The protocol significantly enriches the Ap_2_A-U1 RNA compared to the negative control (Ap_2_A-U1 + NUDT12) or U1 RNAs capped with other caps (m^7^Gp_3_A and pppA). The significance values represent the adjusted *P*-value (ANOVA). (*C*) RNA-Seq results of Ap_2_N-RNA Seq applied to model Ap_2_A-U1 RNA. After removing the sequence of the 5′ adaptor and mapping the reads to the in vitro synthetized U1 reference, the 5′ end of the ligated RNA correctly maps to the +1 position of the U1 sequence in the Ap_2_N sample (green area) compared to ~50% less reads of the negative control sample (+NUDT12, brown area) with rather nonspecific distribution along the sequence.

Next, we aimed to find out which RNAs are capped with Ap_2_N in order to elucidate their metabolism and role in vivo. To this end, we developed a sequencing technique to identify RNAs bearing 5′ Ap_2_N caps (named Ap_2_N-RNA Seq). For this purpose, we leveraged the properties of truncated T4 RNA ligase 2 K227Q, which requires a 5′ preadenylated ssDNA or RNA substrate for ligation to 3′ OH- RNA adaptor. We assumed that, by ligating isolated cellular RNA to a synthetic RNA adaptor and then performing RNA sequencing, we would be able to identify the sequence of natural RNA originally having Ap_2_N caps (Fig. 3*A*). First, we tested the specificity of truncated T4 RNA ligase 2 K227Q toward other RNA caps such as m^7^Gp_3_A-, NAD-, Ap_4_A-, and ADP-ribose. This experiment confirmed Ap_2_A-RNA as the only substrate leading to two ligation products—linear RNA with an attached adaptor and circularized original Ap_2_A-RNA (*SI Appendix*, Fig. S12). Although we did not observe any ligated product with m^7^Gp_3_A-RNA, given the huge excess of m^7^G capped RNA in cells, we included an mRNA decapping enzyme (MDE) treatment to remove this cap prior to the ligation reaction.

As a proof of principle, we performed Ap_2_N-RNA Seq with Ap_2_A-U1 RNA synthetized in vitro (*SI Appendix*, Tables S5 and S6). Based on the reported exonuclease activity of DXO (38), we selected NUDT12—one of the two identified Ap_2_N-RNA decapping enzymes—to generate a negative control for the sequencing protocol. First, we verified that NUDT12 efficiently removes the Ap_2_A cap from IVT prepared Ap_2_A-U1 RNA (*SI Appendix*, Fig. S12). Second, we assessed the formation of the ligation product (Adaptor-U1 RNA) by RT-qPCR (Fig. 3*B*) and observed significant enrichment in Ap_2_A-U1 RNA samples compared to the negative control (Ap_2_A-U1 [+NUDT12]), m^7^Gp_3_A-U1 and pppA-U1. The identity of the PCR product was confirmed by Sanger sequencing (*SI Appendix*, Fig. S12). Next, we implemented other steps (e.g., polyadenylation) to make our protocol compatible with reverse transcription and downstream high-throughput sequencing (Fig. 3*A*). The negative control is essential for the downstream analysis, where candidate hits are defined by fold enrichment in the -NUDT12 sample compared to the +NUDT12 control. The analysis of Ap_2_N-RNA Seq data confirmed the enrichment of the Adaptor-U1 RNA ligation product in Ap_2_A-U1 RNA samples compared to the negative control treatment (Fig. 3*C*).

### Identification of Ap_2_N-RNA in Expi293F Cells.

**Fig. 4.**
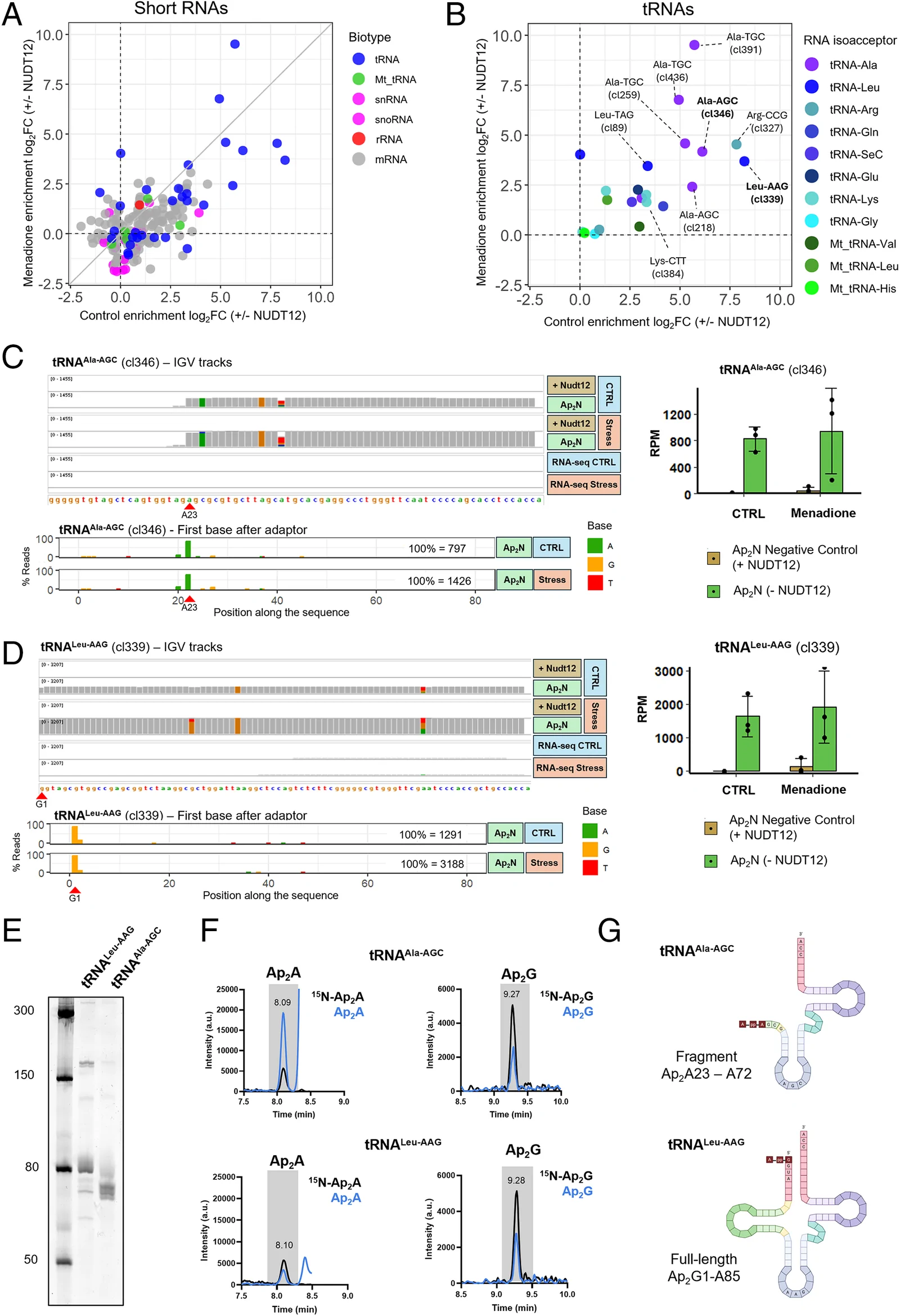
Identification of Ap_2_N-RNAs. (*A*) Scatter plot showing log_2_ fold-change Ap_2_N-RNA enrichment (−NUDT12 Ap_2_N vs. +NUDT12 control) in control cells (x-axis) and menadione-treated samples (y-axis). Average values across replicates are plotted (n = 3, except for control cells, +NUDT12 samples, where n = 2). (*B*) Detail of the scatter plot shown in *A* with particular focus on tRNAs. (*C* and *D*) Integrative genomics viewer (IGV) snapshots of the representative tRNA^Ala-AGC^ (*C*) and tRNA^Leu-AAG^ (*D*) sequencing reads aligned to the reference at different experimental conditions: The control cells (CTRL, indicated by light blue box) and menadione stressed cells (stress, light red box) were subjected to standard short RNA seq (RNA-seq, the two bottom tracks) or Ap_2_N Seq without NUDT12 decapping step (Ap_2_N, green box) or with the NUDT12 decapping step as negative control (+NUDT12, brown box). In both control and menadione-treated samples, the read depth was much greater in −NUDT12 samples (Ap_2_N, green box) compared to +NUDT12 control samples and nontargeted short RNA-Seq. The column graphs represent the quantities of uniquely aligned reads normalized to reads per million (RPM). The black dots represent individual values from biological replicates. The two tracks under the IGV snapshot represent the quantification of the first nucleotide present downstream of the ligated 5′ adaptor sequence and its position within the mapped reads. The number of reads composing 100% on the y-axis is indicated in the track. See *SI Appendix*, Fig. S14 for all other hits and replicates. (*E*) 8% PAGE gel analysis of pull-down RNA of tRNA^Ala-AGC^ and tRNA^Leu-AAG^ used for LC–MS and ONT direct RNA sequencing. (*F*) LC–MS analysis of Ap_2_A and Ap_2_G RNA caps in selected pulled-down hits (tRNA^Ala-AGC^ and tRNA^Leu-AAG^) identified by Ap_2_N-RNA Seq. MRM chromatograms are showing the spiked ^15^N-Ap_2_A (MRM transition 687 → 255) and ^15^N-Ap_2_G (MRM transition 698 → 433) standards (black line) and corresponding caps (MRM transitions 677 → 250 and 693 → 428 for Ap_2_A and Ap_2_G, respectively) (blue line) detected by the MRM method. (*G*) Schematic representation of the tRNA^Ala-AGC^ A23 fragment capped with Ap_2_A and full-length tRNA^Leu-AAG^ capped with Ap_2_G.

Next, we applied the Ap_2_N-RNA Seq protocol to a fraction of sRNA from Expi293F control cells and cells treated with menadione. After trimming and filtering (*SI Appendix*, Fig. S13), we mapped the reads to the human genome and calculated the enrichment in samples treated and not treated with NUDT12 (+/−NUDT12). Interestingly, tRNAs were the most prominently enriched RNAs in samples not treated with NUDT12, suggesting the presence of Ap_2_N at their 5′ end (Fig. 4*A* and *SI Appendix*, Fig. S14 and Dataset S3). To investigate these tRNA hits further, we used specific algorithm dedicated for tRNA mapping (39). The most enriched hits were for example tRNA^Ala-TGC^, tRNA^Ala-AGC^, tRNA^Leu-TAG^, tRNA^Leu-AAG^, tRNA^Lys-TTT^, tRNA^Lys-CTT^, tRNA^Arg-CCG^ (Fig. 4*B* and Dataset S3). Remarkably, we did not observe any substantial difference in the abundance of these hits between control and menadione-treated samples. To exclude the possibility that the RNA hits identified by Ap_2_N-RNA Seq were not enriched due to naturally high intracellular concentrations of the given RNAs, we compared the results of Ap_2_N-RNA Seq analysis with those of a standard short RNA-Seq analysis of Expi293F control and menadione-treated cells (*SI Appendix*, Fig. S13 and Dataset S3). We did not observe any correlation between these two types of datasets, suggesting that the hits obtained by the Ap_2_N-RNA Seq method were enriched in the dataset due to the presence of the Ap_2_N cap. To reveal the exact 5′ position of Ap_2_N within the sequence of the most enriched RNA hits, we focused on the junction between the ligated adaptor and the downstream RNA sequence. As predicted by others (22, 23), we expected Ap_2_N to be formed on exposed, fragile loops of tRNA (i.e., the anticodon region and T or D loops) during the RNA repair process. However, we did not observe any tRNA fragments of such types. We found that whereas tRNA^Leu-TAG^ and other tRNAs (e.g., tRNA^Lys-CTT^, *SI Appendix*, Fig. S14) were ligated to the adaptor in their full form, vast majority of tRNA^Ala-TGC^ and tRNA^Ala-AGC^ were ligated in position A23 (according to common tRNA nomenclature) of their reference sequence (Fig. 4*C* and *SI Appendix*, Fig. S14). This indicates that above-mentioned tRNAs were originally present in form of 3′ fragments capped by Ap_2_A. Surprisingly, this type of tRNA fragment has not been reported yet in the MINTbase 2.0 database (40–42).

To orthogonally confirm the Ap_2_N-RNA Seq results, using antisense DNA probes (*SI Appendix*, Table S5) we pulled down the RNAs of the two most enriched hits (tRNA^Ala-AGC^ and tRNA^Leu-AAG^) as well as one nonenriched RNA (U1 snRNA) serving as negative control (Fig. 4*E* and *SI Appendix*, Fig. S15). All the pulled-down RNAs were further digested and analyzed by LC–MS. As expected, U1 snRNA did not contain any Ap_2_A and showed only trace amounts of Ap_2_G (*SI Appendix*, Fig. S15). Pulled-down tRNA^Ala-AGC^ contained approximately eightfold higher levels of Ap_2_A compared to Ap_2_G (Fig. 4*F*). Although most Ap_2_N-RNA Seq reads supported the presence of Ap_2_A-tRNA fragment, analysis of the mapped reads also identified a small subset of reads in which the adaptor was ligated to the full-length tRNA^Ala^ at the 5′-terminal G (*SI Appendix*, Fig. S16).

Interestingly, the pulled-down tRNA^Leu-AAG^ contained not only the expected Ap_2_G cap but also detectable Ap_2_A. Since the vast majority of tRNA^Leu-AAG^ reads in Ap_2_N-RNA Seq contained the adaptor ligated to the 5′ terminal G, we were unable to explain the presence of Ap_2_A in the pulled-down RNA. However, the PAGE analysis suggested the presence of potentially contaminating RNA species (*SI Appendix*, Fig. S4*D*). Therefore, we analyzed the composition of both pulled-down tRNAs following deacylation and polyadenylation using Oxford Nanopore direct RNA sequencing (ONT dRNA-seq). While the tRNA^Ala-AGC^ sample consisted predominantly of tRNA^Ala^ (approximately 60%), the tRNA^Leu-AAG^ pull-down unexpectedly contained 61% Mt-tRNA^Val-TAC^ (mitochondrial tRNA^Val^) reads and only 20% of the targeted tRNA^Leu^ (*SI Appendix*, Figs. S16 and S17). Because the antisense probe used for the tRNA^Leu-AAG^ pull-down shows no sequence homology to Mt-tRNA^Val-TAC^, the origin of this co-enrichment remains unclear. However, closer inspection of Mt-tRNA^Val-TAC^ reads in the Ap_2_N-RNA Seq dataset revealed adaptor ligation at the 5′-terminal A, which likely accounts for the detection of Ap_2_A in the LC–MS analysis of the tRNA^Leu-AAG^ pull-down (*SI Appendix*, Figs. S14 and S17).

Since the pull-down samples of both tRNAs contained contaminants, we set out to confirm their existence by yet another independent method. First, we performed a northern blot analysis specific for tRNA^Ala-TGC^ on sRNA treated with and without NUDT12 and subsequently ligated to a 5′ adaptor via a potential Ap_2_N moiety. Unfortunately, this experiment did not provide any conclusive result, presumably because the concentration of the tRNA fragment was too low. Therefore, we carried out an additional sequencing experiment to confirm the existence of the Ap_2_A-tRNA^Ala-TGC^ fragment and Ap_2_G-tRNA^Leu-AAG^. In principle, we performed the same protocol as shown in Fig. 3*A*, but after ligation of the adaptor, we did not proceed with general PCR amplification and sequencing. Instead, the RNA was reverse transcribed with a primer specific for the 3′ end of tRNA^Ala-TGC^or Ap_2_G-tRNA^Leu-AAG^ (*SI Appendix*, Table S5). Subsequently, we amplified cDNA with a forward adaptor primer and the same primer used for RT. The resulting amplicons were cut from the agarose gels (*SI Appendix*, Figs. S16 and S17), cloned into plasmids and sequenced by the Sanger procedure. Indeed, we observed the adaptor ligated to the tRNA^Ala-TGC^ fragment at position A23 (*SI Appendix*, Fig. S16) in two independent experiments using two different adaptors and two reverse transcriptases. Moreover, we also observed adaptor attached to the 5′ end of Ap_2_G-tRNA^Leu-AAG^. The results of these experiments confirm the existence of a 3′ tRNA fragments capped with Ap_2_A and full-length Ap_2_G capped Ap_2_G-tRNA^Leu-AAG^.

To further support our findings, we separated 1.6 mg of sRNA isolated from Expi293F control cells by denaturing PAGE and excised regions corresponding to tRFs (A), type I tRNAs (B), type II tRNAs (C) (43), and three regions migrating above tRNAs [D–F; region E contained U1 snRNA and 5.8S rRNA (44–46)] (*SI Appendix*, Fig. S18). From each gel fraction, 7 µg RNA was digested as described above. A small aliquot of the digest (200 ng) was analyzed by an LC–MS method targeting internal tRNA modifications, whereas the remaining digest was purified by SPE and analyzed using the LC–MS method developed for Ap_2_N caps. LC–MS analysis of internal modifications detected characteristic tRNA modifications, including 1-methylguanosine (m^1^G), 2-methylguanosine (m^2^G), and 5-methyluridine (m^5^U), in fractions A–C (tRFs, type I tRNAs, and type II tRNAs) (47, 48) (*SI Appendix*, Fig. S19). LC–MS analysis of Ap_2_N caps detected Ap_2_A in the tRF (A) and type II tRNA (C) fractions, and Ap_2_G in both tRNA fractions (B and C) as well as in fraction D (*SI Appendix*, Fig. S20). Notably, traces of tRNA-characteristic internal modifications were also detected in fraction D (*SI Appendix*, Fig. S19), consistent with the presence of minor tRNA material in this region. Because fraction D migrates immediately above the tRNA bands, the Ap_2_G signal is most plausibly explained by limited carryover or partial overlap during gel excision rather than a distinct non-tRNA RNA species. Collectively, these results support that Ap_2_A and Ap_2_G caps are enriched in tRNA/tRF-containing fractions and are consistent with the Ap_2_N-RNA Seq findings.

## Discussion

In summary, we used LC–MS to identify and quantify Ap_2_N RNA caps, particularly Ap_2_A and Ap_2_G, in human cells. Their abundance increased in RNA isolated from cells exposed to oxidative stress. We further demonstrated that human RLIG1 can generate Ap_2_N-capped RNAs in vitro. However, in vivo data indicated that RLIG1 contributed only ~20% of the total Ap_2_N-RNA pool, suggesting the existence of additional sources. Notably, we detected free Ap_2_A, which could potentially be incorporated into RNA as an NCIN, analogous to other Np*_n_*Ns (14).

To identify RNAs bearing Ap_2_N at their 5′ termini, we developed a selective method termed Ap_2_N-RNA Seq. Application of this approach to sRNA isolated from control and menadione-treated cells identified tRNA-derived species as the predominant candidate Ap_2_N-capped RNAs. To date, the only reported tRNA capping event is the canonical m^7^G cap found on tRNA precursors (49). LC–MS analysis of selected pull-down hits provided orthogonal support for the presence of Ap_2_N caps in candidate-enriched RNA fractions and further supported the Ap_2_N-RNA Seq workflow. Interestingly, we did not observe any systemic enrichment of RNA hits in RNA isolated from menadione-treated cells in comparison with control cells. This finding contradicts the quantification of Ap_2_N by LC–MS, where we observed a significantly larger amount of Ap_2_N-RNA. The explanation could be that Ap_2_N-RNA is formed by two different processes. Potentially there might be a single basal population of RNA having Ap_2_N at the 5′ termini, which we have been able to identify by the Ap_2_N-RNA Seq method. The second population of Ap_2_N-RNA might be formed during oxidative damage to RNA at rather random positions during the process of RNA repair by RLIG1, as has been proposed by others (22) or by an unknown mechanism. These molecules contribute to the total amount of Ap_2_N measured in NuP1 digested RNA by LC–MS, but, on the other hand, the rather random distribution of breaks would not lead to a detectable enrichment in our Ap_2_N-RNA Seq analysis. However, the validation of this hypothesis is out of scope of this report and will be the subject of a future study.

The major hits of Ap_2_N-RNA Seq corresponded to processed, full-length tRNAs (e.g., tRNA^Leu^, tRNA^Lys^) carrying Ap_2_G at their 5′ termini. Importantly, tRNA^Lys-CTT^ was previously reported to co-purify with native human RLIG1 (23), consistent with a potential role of this enzyme in Ap_2_G-tRNA formation. We also identified previously unreported 3′ tRNA fragments derived from tRNA^Ala-TGC^ and tRNA^Ala-AGC^ that are consistent with Ap_2_A capping at position A23. Although this specific fragment type is not annotated in MINTbase v2.0, multiple corresponding upstream 5′ tRF-Ala fragments are listed without further characterization. Notably, one such 5′ tRF-Ala fragment (tDR-1:20) has been reported to regulate mRNA abundance and splicing in *Arabidopsis thaliana* (50). Importantly, the detected fragments and caps are unlikely to arise from the canonical tRNA intron–excision/RTCB ligation pathway, given the distinct tRNA species and fragment sequence observed here and the 3′ to 5′ linkage in Ap_2_G intermediate of RTCB ligation (51–53). Next, we asked how abundant the parental tRNAs of the identified fragments are in cells. Because our RNA-seq protocol does not capture charged tRNAs, we analyzed a dedicated tRNA quantification dataset (54) generated from human brain tissue and HEK293T cells, the latter being closely related to Expi293F cells. Surprisingly, the parental tRNAs corresponding to our fragments (clusters 218, 346, 259, 436, and 391) represented less than 8% of the tRNA^Ala-TGC^ and tRNA^Ala-AGC^ isodecoder pools in both samples. In contrast, the most abundant tRNA^Ala-TGC^ and tRNA^Ala-AGC^ isodecoders, which together account for approximately 75 to 80% of these pools, were not among the most enriched Ap_2_N-RNA Seq hits (*SI Appendix*, Table S7). These observations suggest that Ap_2_N-capped tRNAs and their fragments may represent a specialized subset with functions distinct from canonical translation. The biological roles of these fragments and other Ap_2_N-RNA species identified in this study remain to be determined and will be addressed in future work. We speculate that Ap_2_N-RNAs could serve as substrates for as-yet-unidentified ligases involved in the generation of circular or chimeric RNAs. More broadly, we anticipate that the Ap_2_N-RNA Seq method will facilitate future investigations into the formation, turnover, and biological functions of Ap_2_N-RNAs.

### Limitation of the Study.

In this work we focused on Ap_2_A and Ap_2_G and optimized the LC–MS/MS workflow accordingly. Reliable analysis of other Ap_2_N caps (such as Ap_2_C and Ap_2_U) would require a dedicated analytical pipeline, including optimization of digestion and LC–MS conditions, and was therefore not pursued here. In addition, the present Ap_2_N-RNA Seq adaptor design does not allow unambiguous inference of Ap_2_C, because the adaptor’s 3′ cytidine stretch is removed during adaptor trimming. The paired ±NUDT12 design is crucial for specificity of the method, nevertheless, the NUDT12-treated control by the matter of principle contains fewer template molecules, which requires higher number of PCR amplification cycles in the method. This might lead to increased background in the negative control primarily reducing the fold change and thus decreasing sensitivity. Consequently, the approach does not allow direct quantification of individual hits. Targeted tRNA pull-downs can also co-enrich unrelated RNAs; we therefore profiled pull-down composition by direct Nanopore sequencing, revealing that purity can be limited and should be assessed explicitly. Finally, because Ap_2_N-RNA Seq was designed as an exploratory, broadly applicable workflow, without prior assumptions about RNA class, RNA samples were not subjected to tRNA-specific treatments prior to library preparation, such as deacylation or demethylation. Thus, our sequencing data likely underrepresent aminoacylated (charged) tRNAs and may also be affected by mature tRNA structure and nucleotide modifications which can interfere with SuperScript IV and cause misincorporations at various sites. Since tRNAs were identified as the strongest hits, future studies will focus on disentangling the origin and role of Ap_2_N capping across different forms of tRNA. Overall, despite these limitations, our work provides an enabling methodology and broadens current understanding of tRNA and tRNA-fragment biology through the analysis of Ap_2_N capping.

## Material and Methods

### Cell Culture.

Expi293F cells were maintained in suspension culture in Expi293 Expression Medium (ThermoFisher A1435101) shaking at 120 RPM at 37 °C and 8% CO_2_. The cells were split after reaching concentration of 3 to 5 × 10^6^ cells/mL to the density 0.3 to 0.5 × 10^6^ cells/mL. Menadione (Sigma Aldrich M2518-100G, stock solution 2 mM in 96% EtOH) treatment was performed for 3.5 h with 40 μM final concentration and the negative control was treated with the same volume of 96% EtOH.

### Generation of RLIG1 Knock-Out in Expi293F Cell Line.

The RLIG1 knock-out line and the control Scrambled (SCR) cell lines were generated by lentiviral vectors containing Cas9 and two sgRNAs targeting Exon4 and Exon6 of the RLIG1 gene, or scrambled sgRNA sequences, respectively. Single-cell clones were sorted and screened for RLIG1 protein expression. Relevant clones were further confirmed by Sanger sequencing of the genomic DNA.

### RNA Isolation and Digestion for LC–MS Analysis.

RNA isolation was performed using 1 mL of RNAzol (Sigma-Aldrich #R4533) per 10^7^ cells according to the manufacturer’s instructions for short and long RNA fraction isolation. Isolated RNA (120 µg) was digested by Nuclease P1 (60 U), followed by rSAP treatment (3 U) and spiked with 3 µL of ^15^N-Ap_2_A (2 nM) and ^15^N-Ap_2_G standards (8 nM). After the digestion, the samples were purified by SPE to remove nucleosides and retain phosphate containing RNA caps. Samples were concentrated in vacuo and redissolved in 15 µL of 50 mM ammonium acetate. Control samples were going through the same protocol without the addition of Nuclease P1 enzyme. The samples were analyzed using HPLC system (Acquity Premier, Waters) equipped with an Acquity BEH Amide column and Mass spectrometer (Xevo TQ Absolute, Waters) and multiple-reaction monitoring method (MRM). The Ap_2_A and Ap_2_G quantitation was done from three biological replicates.

### Ap_2_N-RNA Seq Protocol.

5 μg of the short RNA fraction (or 1 μg in vitro synthetized U1 RNA for the protocol establishment) was decapped by NUDT12 (5 μM in 1× decapping buffer 10 mM Tris.HCl pH 8.5, 100 mM KCl, 2 mM DTT, 2 mM MgCl_2_, and 2 mM MnCl_2_) for negative control samples or incubated in the buffer without the NUDT12 enzyme (Ap_2_N sample). Zymo Clean and Concentrator 5 (Zymo Research #D4014) was used for all purification steps between enzymatic treatments. Afterward, all samples were treated with mRNA decapping enzyme (MDE, NEB #M0608) to remove canonical mRNA cap and subsequently with Terminator Exonuclease (Lucigen #TER51020) to degrade all noncapped RNA. Next, the RNA was polyA tailed with *E. coli* Poly(A) Polymerase (NEB #M0276) and the 5′ adaptor (*SI Appendix*, Table S5) was ligated using of T4 RNA ligase 2 truncated K227Q (NEB, #M0351). cDNA was prepared using SuperScript™ IV First-Strand Synthesis System (ThermoFisher Scientific #18091050) and PCR amplified by Phusion® High-Fidelity DNA Polymerase (NEB #M0530) and finally purified using Agencourt® RNACleanTM XP (Beckman Coulter #A66514). The Illumina sequencing was performed commercially at Novogene (Cambridge, UK), using standard dsDNA 2 × 150 bp paired-end sequencing at the NovaSeq platform providing ~40 Mr per sample. The overlapping paired-end reads were merged using fastp (0.20.1) (7) and only reads containing single copy of both adaptors (the 5′ Ap2N-RNA-Seq and the reverse polyT-adaptor, *SI Appendix*, Table S5) in appropriate orientation were kept. After adaptor trimming a modified pipeline of Hoffmann et al. (9, 39) was used (*SI Appendix*, Fig. S17 and *Methods*).

More detailed materials and methods are available in *SI Appendix*.

## Supplementary Material

Appendix 01 (PDF)

Dataset S01 (XLSX)

Dataset S02 (XLSX)

Dataset S03 (XLSX)

## Data Availability

The authors have cited additional references within *SI Appendix*. The Illumina sequencing data are stored in the ENA database under the Accession No. PRJEB85513 (55). The mass spectrometry data are stored at Zenodo (https://doi.org/10.5281/zenodo.18978998) (56).

## References

[1] A. G. McLennan, Dinucleoside polyphosphates—friend or foe? Pharmacol. Ther. **87**, 73–89 (2000). 10.1016/s0163-7258(00)00041-3 11007992

[2] X. Ji , Alarmone Ap4A is elevated by aminoglycoside antibiotics and enhances their bactericidal activity. Proc. Natl. Acad. Sci. U.S.A. **116**, 9578–9585 (2019). 10.1073/pnas.1822026116 PMC6511005 31004054

[3] B. R. Bochner, P. C. Lee, S. W. Wilson, C. W. Cutler, B. N. Ames, AppppA and related adenylylated nucleotides are synthesized as a consequence of oxidation stress. Cell **37**, 225–232 (1984). 10.1016/0092-8674(84)90318-0 6373012

[4] F. Ferguson, A. G. McLennan, M. D. Urbaniak, N. J. Jones, N. A. Copeland, Re-evaluation of diadenosine tetraphosphate (Ap4A) from a stress metabolite to bona fide secondary messenger. Front. Mol. Biosci. **7**, 606807 (2020). 10.3389/fmolb.2020.606807 PMC7705103 33282915

[5] K. H. Götz , Formation of the alarmones diadenosine triphosphate and tetraphosphate by ubiquitin- and ubiquitin-like-activating enzymes. Cell Chem. Biol. **26**, 1535–1543.e5 (2019). 10.1016/j.chembiol.2019.08.004 31492597

[6] P. G. Zamecnik, M. L. Stephenson, C. M. Janeway, K. Randerath, Enzymatic synthesis of diadenosine tetraphosphate and diadenosine triphosphate with a purified lysyl-sRNA synthetase. Biochem. Biophys. Res. Commun. **24**, 91–97 (1966). 10.1016/0006-291x(66)90415-3 5338216

[7] O. Goerlich, R. Foeckler, E. Holler, Mechanism of synthesis of adenosine(5’)tetraphospho(5’)adenosine (AppppA) by aminoacyl-tRNA synthetases. Eur. J. Biochem. **126**, 135–142 (1982). 10.1111/j.1432-1033.1982.tb06757.x 7128581

[8] A. G. McLennan, The Nudix hydrolase superfamily. Cell. Mol. Life Sci. **63**, 123–143 (2006). 10.1007/s00018-005-5386-7 PMC11136074 16378245

[9] V. Jankowski , Dinucleoside polyphosphates: Strong endogenous agonists of the purinergic system. Br. J. Pharmacol. **157**, 1142–1153 (2009). 10.1111/j.1476-5381.2009.00337.x PMC2743832 19563527

[10] O. Hudeček , Dinucleoside polyphosphates act as 5′-RNA caps in bacteria. Nat. Commun. **11**, 1052 (2020). 10.1038/s41467-020-14896-8 PMC7044304 32103016

[11] D. J. Luciano, R. Levenson-Palmer, J. G. Belasco, Stresses that raise Np4A levels induce protective nucleoside tetraphosphate capping of bacterial RNA. Mol. Cell **75**, 957–966.e8 (2019). 10.1016/j.molcel.2019.05.031 PMC6731127 31178354

[12] R. Benoni, M. Culka, O. Hudeček, L. Gahurova, H. Cahová, Dinucleoside polyphosphates as RNA building blocks with pairing ability in transcription initiation. ACS Chem. Biol. **15**, 1765–1772 (2020). 10.1021/acschembio.0c00178 32530599

[13] D. J. Luciano, J. G. Belasco, Np(4)A alarmones function in bacteria as precursors to RNA caps. Proc. Natl. Acad. Sci. U.S.A. **117**, 3560–3567 (2020). 10.1073/pnas.1914229117 PMC7035476 32019889

[14] V. M. Serianni , Molecular insight into 5′ RNA capping with Np*n*Ns by bacterial RNA polymerase. Nat. Chem. Biol. **22**, 917–924 (2026). 10.1038/s41589-025-02134-5 PMC13226041 41513851

[15] W. Duan , Molecular basis for noncanonical transcription initiation from Np4A alarmones. Nat. Chem. Biol. **22**, 906–916 (2025). 10.1038/s41589-025-02044-6 PMC12781554 41094128

[16] J. František Potužník , Diadenosine tetraphosphate (Ap4A) serves as a 5′ RNA cap in mammalian cells. Angew. Chem. Int. Ed. Engl. **63**, e202314951 (2024). 10.1002/anie.202314951 37934413

[17] Y. Ofir-Birin , Structural switch of lysyl-tRNA synthetase between translation and transcription. Mol. Cell **49**, 30–42 (2013). 10.1016/j.molcel.2012.10.010 PMC3766370 23159739

[18] J. Luo , Identification and characterization of diadenosine 5′, 5‴-P1, P2-diphosphate and diadenosine 5′, 5‴-P1, P3-triphosphate in human myocardial tissue. FASEB J. **13**, 695–705 (1999). 10.1096/fasebj.13.6.695 10094930

[19] J. Jankowski , Identification of dinucleoside polyphosphates in adrenal glands. Biochem. Biophys. Res. Commun. **304**, 365–370 (2003). 10.1016/s0006-291x(03)00596-5 12711324

[20] J. Jankowski , Dinucleotides as growth-promoting extracellular mediators: Presence of dinucleoside diphosphates Ap2A, Ap2G, and Gp2G in releasable granules of platelets. J. Biol. Chem. **276**, 8904–8909 (2001). 10.1074/jbc.M009527200 11115507

[21] M. Magnone , Adenylic dinucleotides produced by CD38 are negative endogenous modulators of platelet aggregation. J. Biol. Chem. **283**, 24460–24468 (2008). 10.1074/jbc.M710568200 PMC3259817 18606819

[22] Y. Yuan , Chemoproteomic discovery of a human RNA ligase. Nat. Commun. **14**, 842 (2023). 10.1038/s41467-023-36451-x PMC9931718 36792600

[23] Y. Hu , Biochemical and structural insights into a 5’ to 3’ RNA ligase reveal a potential role in tRNA ligation. Proc. Natl. Acad. Sci. U.S.A. **121**, e2408249121 (2024). 10.1073/pnas.2408249121 PMC11494293 39388274

[24] J. C. Baker, M. K. Jacobson, Alteration of adenyl dinucleotide metabolism by environmental stress. Proc. Natl. Acad. Sci. U.S.A. **83**, 2350–2352 (1986). 10.1073/pnas.83.8.2350 PMC323294 3458199

[25] S. Alizadeh, G. Anani-Sarab, H. Amiri, M. Hashemi, Paraquat induced oxidative stress, DNA damage, and cytotoxicity in lymphocytes. Heliyon **8**, e09895 (2022). 10.1016/j.heliyon.2022.e09895 PMC9287805 35855999

[26] S. Alireza Ghodsi, M. Joseph Matthew, M. Amanda, G. Michelle, D. Khoa Dang, Menadione induces DNA damage and superoxide radical level in HEK293 cells. Cell Biol. **7**, 14–22 (2019).

[27] J. Chen, M. K. Ghorai, G. Kenney, J. Stubbe, Mechanistic studies on bleomycin-mediated DNA damage: Multiple binding modes can result in double-stranded DNA cleavage. Nucleic Acids Res. **36**, 3781–3790 (2008). 10.1093/nar/gkn302 PMC2441780 18492718

[28] J. F. Potužník, H. Cahova, If the 5’ cap fits (wear it)—Non-canonical RNA capping. RNA Biol. **21**, 1–13 (2024). 10.1080/15476286.2024.2372138 PMC11253889 39007883

[29] M.-B. Mititelu , *Arabidopsis thaliana* NudiXes have RNA-decapping activity. RSC Chem. Biol. **4**, 223–228 (2023). 10.1039/d2cb00213b PMC9994101 36908703

[30] X. Jiao , 5′ end nicotinamide adenine dinucleotide cap in human cells promotes RNA decay through DXO-mediated deNADding. Cell **168**, 1015–1027.e10 (2017). 10.1016/j.cell.2017.02.019 PMC5371429 28283058

[31] M.-G. Song, Y. Li, M. Kiledjian, Multiple mRNA decapping enzymes in mammalian cells. Mol. Cell **40**, 423–432 (2010). 10.1016/j.molcel.2010.10.010 PMC2982215 21070968

[32] E. Grudzien-Nogalska , Structural and mechanistic basis of mammalian Nudt12 RNA deNADding. Nat. Chem. Biol. **15**, 575–582 (2019). 10.1038/s41589-019-0293-7 PMC6527130 31101919

[33] S. Sharma , Mammalian nudix proteins cleave nucleotide metabolite caps on RNAs. Nucleic Acids Res. **48**, 6788–6798 (2020). 10.1093/nar/gkaa402 PMC7337524 32432673

[34] C. L. Hawkins, M. J. Davies, Detection, identification, and quantification of oxidative protein modifications. J. Biol. Chem. **294**, 19683–19708 (2019). 10.1074/jbc.REV119.006217 PMC6926449 31672919

[35] C. K. Ho, S. Shuman, Bacteriophage T4 RNA ligase 2 (gp24.1) exemplifies a family of RNA ligases found in all phylogenetic domains. Proc. Natl. Acad. Sci. U.S.A. **99**, 12709–12714 (2002). 10.1073/pnas.192184699 PMC130525 12228725

[36] A. Martins, S. Shuman, An RNA ligase from *Deinococcus radiodurans**. J. Biol. Chem. **279**, 50654–50661 (2004). 10.1074/jbc.M407657200 15333634

[37] M.-C. Unciuleac, Y. Goldgur, S. Shuman, Structure and two-metal mechanism of a eukaryal nick-sealing RNA ligase. Proc. Natl. Acad. Sci. U.S.A. **112**, 13868–13873 (2015). 10.1073/pnas.1516536112 PMC4653202 26512110

[38] X. Jiao, J. H. Chang, T. Kilic, L. Tong, M. Kiledjian, A mammalian pre-mRNA 5’ end capping quality control mechanism and an unexpected link of capping to pre-mRNA processing. Mol. Cell **50**, 104–115 (2013). 10.1016/j.molcel.2013.02.017 PMC3630477 23523372

[39] A. Hoffmann , Accurate mapping of tRNA reads. Bioinformatics **34**, 1116–1124 (2018). 10.1093/bioinformatics/btx756 29228294

[40] S. Muthukumar, C.-T. Li, R.-J. Liu, C. Bellodi, Roles and regulation of tRNA-derived small RNAs in animals. Nat. Rev. Mol. Cell Biol. **25**, 359–378 (2024). 10.1038/s41580-023-00690-z 38182846

[41] Z. Su, B. Wilson, P. Kumar, A. Dutta, Noncanonical roles of tRNAs: tRNA fragments and beyond. Annu. Rev. Genet. **54**, 47–69 (2020). 10.1146/annurev-genet-022620-101840 PMC7686126 32841070

[42] V. Pliatsika , MINTbase v2.0: A comprehensive database for tRNA-derived fragments that includes nuclear and mitochondrial fragments from all The Cancer Genome Atlas projects. Nucleic Acids Res. **46**, D152–d159 (2018). 10.1093/nar/gkx1075 PMC5753276 29186503

[43] Y. Kim, K. Opron, Z. F. Burton, A tRNA- and anticodon-centric view of the evolution of aminoacyl-tRNA synthetases, tRNAomes, and the genetic code. Life **9**, 37 (2019). 10.3390/life9020037 PMC6616430 31060233

[44] K. Klimešová, H. Petržílková, C. Bařinka, D. Staněk, SART3 associates with a post-splicing complex. J. Cell Sci. **136**, jcs260380 (2023). 10.1242/jcs.260380 36620952

[45] K. Klimešová , TSSC4 is a component of U5 snRNP that promotes tri-snRNP formation. Nat. Commun. **12**, 3646 (2021). 10.1038/s41467-021-23934-y PMC8206348 34131137

[46] D. A. Basello , Dynamic interaction of spliceosomal snRNPs with coilin explains Cajal body characteristics. J. Cell Biol. **224**, e202309128 (2025). 10.1083/jcb.202309128 PMC12189012 40560171

[47] D. Su , Quantitative analysis of ribonucleoside modifications in tRNA by HPLC-coupled mass spectrometry. Nat. Protoc. **9**, 828 (2014). 10.1038/nprot.2014.047 PMC4313537 24625781

[48] M. Zhang, Z. Lu, TRNA modifications: Greasing the wheels of translation and beyond. RNA Biol. **22**, 1–25 (2025). 10.1080/15476286.2024.2442856 PMC12710937 39723662

[49] T. Ohira, T. Suzuki, Precursors of tRNAs are stabilized by methylguanosine cap structures. Nat. Chem. Biol. **12**, 648–655 (2016). 10.1038/nchembio.2117 27348091

[50] Y. Li , The functions of a 5’ tRNA-Ala-derived fragment in gene expression. Plant Physiol. **193**, 1126–1141 (2023). 10.1093/plphys/kiad361 37350495

[51] J. L. Gerber , Structural and mechanistic insights into activation of the human RNA ligase RTCB by archease. Nat. Commun. **15**, 2378 (2024). 10.1038/s41467-024-46568-2 PMC10944509 38493148

[52] A. K. Chakravarty, R. Subbotin, B. T. Chait, S. Shuman, RNA ligase RtcB splices 3’-phosphate and 5’-OH ends via covalent RtcB-(histidinyl)-GMP and polynucleotide-(3’)pp(5’)G intermediates. Proc. Natl. Acad. Sci. U.S.A. **109**, 6072–6077 (2012). 10.1073/pnas.1201207109 PMC3341019 22474365

[53] X. Zhang , Structural basis of pre-tRNA intron removal by human tRNA splicing endonuclease. Mol. Cell **83**, 1328–1339.e4 (2023). 10.1016/j.molcel.2023.03.015 37028420

[54] A. G. Torres, O. Reina, C. Stephan-Otto Attolini, L. Ribas de Pouplana, Differential expression of human tRNA genes drives the abundance of tRNA-derived fragments. Proc. Natl. Acad. Sci. U.S.A. **116**, 8451–8456 (2019). 10.1073/pnas.1821120116 PMC6486751 30962382

[55] P. Vopalensky , Illumina sequencing data. ENA. https://www.ebi.ac.uk/ena/browser/view/PRJEB85513. Deposited 5 March 2025.

[56] A. Skriba , Mass spectrometry data. Zenodo. 10.5281/zenodo.18978998. Deposited 12 March 2026.

