# Supplementary material for: Detection and sequencing of Ap2N-capped RNAs in human cells
Source: Proc Natl Acad Sci U S A. 2026 Sep 15;123(38):e2608168123. doi: 10.1073/pnas.2608168123 (PMC13598233; doi:10.1073/pnas.2608168123)
Supplement: Supplementary file 1 [file pnas.2608168123.sapp.pdf]

Supporting Information

Discovery and Sequencing of Ap_2N-Capped RNAs in Human Cells

Pavel Vopalensky[a] [c], Ondřej Nešuta[a] [c], Maria-Bianca Mititelu[a][b][c], Anton Škríba[a] [c], Ambra Spampinato[a] Klára Viktorinová[a], Zuzana Buchová[a], Jana Březinová[a], Paul E. Reyes-Gutierrez[a], Ondřej Lukšan[a] and Hana Cahova*[a]

[a] Pavel Vopalensky, Ondřej Nešuta, Maria-Bianca Mititelu, Anton Škríba, Ambra Spampinato, Klára Viktorinová, Zuzana Buchová, Jana Březinová, Paul E. Reyes-Gutierrez, Ondřej Lukšan and Hana Cahova
Institute of Organic Chemistry and Biochemistry of the CAS
Flemingovo náměstí 2, Prague 6 (Czechia)

[b] Maria-Bianca Mititelu
Department of Cell Biology, Faculty of Science
Charles University
Viničná 7, Prague 2 (Czechia)

[c] These authors contributed equally: Pavel Vopalensky, Ondřej Nešuta, Maria-Bianca Mititelu, and Anton Škríba

*Corresponding author: Hana Cahova
Email: cahova@uochb.cas.cz

Contents:

Methods

Schemes S1-S6

Tables S1-S7

Figures S1-S42

Cell culture

Expi293F cells were provided by Zuzana Vaitová from Kvido Stříšovský group (IOCB Prague). They were maintained in suspension culture in $Expi^{293}$ Expression Medium (ThermoFisher A1435101) shaking at 120 RPM at 37 °C and 8% CO_2. The cells were split after reaching concentration of 3-5x10^6 cells/mL to the density 0.3-0.5x10^6 cells/mL.

Menadione treatment

3x10^5 cells were cultured in 200 mL of $Expi^{293}$ Expression Medium and 3 days post-subculture cells were directly treated with 20 to 100 µM final concentration of menadione (Sigma Aldrich M2518-100G, stock solution 2 mM in 96% EtOH) or with 96% EtOH (Control cells). After 3.5 h of incubation, cells were harvested by centrifugation for 5 min at 300 × g at 4 °C and washed with ice-cold 1x PBS. The cells were stored at -70 °C until the RNA isolation was performed. To quantify oxidative status of the cells, total glutathione (GSH + GSSG) and GSH/GSSG ratio were measured using the ROS-Glo™ H_2O_2 assay (Promega #G8820) according to the manufacturer's instructions. For all subsequent experiments, 100 million of cells in 35 mL of $Expi^{293}$ Expression Medium were treated with menadione at final concentration of 40 µM for 3.5 hours. Afterwards, the cells were immediately put on ice, and harvested by centrifugation 400 × g for 5 minutes at 4 °C. The cellular pellet was snap frozen in liquid nitrogen and kept at -70 °C until further processing.

Paraquat and bleomycine treatment

The treatment with paraquat (Scientific Laboratory Supplies # 36541-100MG) and bleomycine (ThermoFisher # J60727.MA) was performed in a similar way as described above for menadione treatment with several exceptions: i) sterile water was used as negative (vehicle) control, ii) the concentration of paraquat in samples for RNA isolation was 160 µM and for bleomycine treatment 5 µg/mL iii) The efficacy of bleomycine treatment was assessed by western blot using rabbit Phospho-Histone H2A.X (Ser139) Antibody (Cell Signaling Technology #2577) at dilution 1:1000.

Preparation of lentiviral vectors for CRIPSR/Cas9 RLIG1 KO and Scrambled modification in HEK293T cell line

HEK293T cells were transfected at 20% confluency in a 100 mm plate with a mixture of two helper plasmids pCMV–VSV–G (4.5 µg) and pCMV–dR82dvpr (4.5 µg) (obtained from the Keckesova lab, IOCB Prague), and 6 µg plasmid containing either two gRNA sequences targeting RLIG1 locus (VectorBuilder https://en.vectorbuilder.com/vector/VB240207-1012mxf.html) or Scrambled guide RNAs as negative control (VectorBuilder https://en.vectorbuilder.com/vector/VB010000-9355sqw.html) using the Lipofectamine 3000 Reagent (ThermoFisher #L3000001). Three days after transfection, 12 mL of cell–free supernatant medium containing the lentiviral particles was harvested, filtered through 0.45 µm filter, and stored at -80 °C until further use.

Generation of RLIG1 knock-out Expi293F cell line

For transduction, Expi293F cells were incubated in adherent state in DMEM-F12 (DMEM/F-12 medium. At the confluency of 20%, 1 mL of harvested lentiviral particles was dropped onto the plate. After 72 hours, the supernatant was removed and substituted by fresh DMEM medium containing 1 µg/mL puromycin for selection. After the selection and reaching

~10 million of cells, the cells were sorted as single-cells into three 96-well plate containing 100 µL preconditioned DMEM-F12 medium. Twelve single-clone colonies for each condition (RLIG1 KO and SCR) were screened by western blot for the knock-out of RLIG1 using mouse C12orf29 (D-9) antibody (Santa Cruz Biotechnology Inc. sc-390730) at 1:200 dilution. From the screen, three clones negative for the RLIG1 signal on the western blot and three clones with normal signal of RLIG1 for Scrambled control were further expanded and used for characterization of the genomic mutation.

To assess the genetic change responsible for the knock-out, the genomic loci spanning ~150 bp upstream and downstream of the gRNA target sites were PCR amplified and Sanger-sequenced. The genomic DNA was isolated from 500.000 cells using Monarch spin genomic DNA purification kit (NEB # T3010S) and 100 ng were subjected to 30 PCR cycles using DreamTaq DNA polymerase (ThermoFisher # EP1701) and primer pairs spanning the region of interest (Table S5) according to supplier instructions. The resulting amplicons were Sanger-sequenced using the Fwd amplification primer. The sequencing results were examined and the homogenous clone having clean and exact sequencing track leading to frameshift in RLIG1 coding-sequence (clone 2B7) was selected. Scrambled clone checked for normal genomic sequence was used as negative control.

Intracellular metabolites extraction (free Ap_2N)

All solvents used for extraction and purification were at least HPLC grade (Fisher), Acetonitrile and MeOH were LC-MS grade (Fisher Optima).

Extraction of polar metabolites was performed based on published method (1) with small modifications. Briefly, PBS washed Expi293F cells (0.5-1 million) were taken from -70 °C and immediately mixed in low DNA binding reaction tube (1.5 mL, Sarstedt #72.706.700) with 500 µL of pre-cooled (7 °C) extraction solution containing 1% ammonia in H_2O:ACN:MeOH (1:1:2) and ^{15}N-Ap_2A and ^{15}N-Ap_2G (both 1 nM) standards. Right after, the solution was mixed at 2000 rpm for 10 min at 10 °C with thermo-shaker (Biosan). Mixture was then centrifuged (Eppendorf centrifuge) at 18 000 × g, 4 °C for 30 min. 200 µL of decanted sample was then concentrated in vacuo (SpeedVac, Thermo), dissolved in 100 µL of 10% MeOH in 50 mM ammonium acetate (Invitrogen #AM9071), mixed at 2000 rpm for 10 min at 10 °C and centrifuged at 18 000 × g, 4 °C for 30 min. 90 µL of sample was then transferred to HPLC vial (polypropylene) and analysed by HILIC LC-MS.

RNA isolation

RNA isolation was performed using RNAzol (Sigma-Aldrich #R4533) according to the manufacturer's instructions. The cell lysis was performed using 1 mL of RNAzol per 10^7 cells. Precipitation of DNA, proteins and polysaccharide was achieved by adding 0.4 mL of RNase-free water per mL of RNAzol. The samples were vortexed and incubated for 15 min on ice, following centrifugation for 45 min at 6000 × g and 4 °C. For the precipitation of long RNA, the supernatant was transferred to a new tube and 0.4 mL of 75% EtOH per 1 mL of the supernatant was added. The samples were vortexed and left 10 min on ice followed by centrifugation for 16 min at 6000 × g and 4 °C. The resulting pellet is considered the long RNA fraction. To achieve short RNA isolation, another precipitation was done by transferring the supernatant remaining after long RNA pellet into another tube and adding 0.8 mL of pure isopropanol per 1 mL of the supernatant. The pellet was washed twice with 70% isopropanol. Finally, the pellet was resuspended in 100 µL of molecular biology grade water. RNA was stored at -70 °C for further use.

Digestion of short RNA

Isolated RNA was washed 4 times with 500 µL of molecular biology grade water using 10 kDa cut-off filters (Amicon, Merck) to reduce the amount of non-covalently bound molecules. Short RNA (120 µg) was digested in reaction containing 60 U of Nuclease P1 (NEB #M0660S), 10 µL of 500 mM ammonium acetate (pH 5.5) and molecular biology grade water in 100 µL total volume. Reaction was incubated for 1 h at 37 °C. Right after 3 U of Shrimp Alkaline Phosphatase (rSAP, NEB #M0371L), 15 µL of 500 mM ammonium acetate (pH 9.1), 3 µL of ^{15}N-Ap_2A (2 nM) and ^{15}N-Ap_2G standards (8 nM) and molecular biology grade water in 200 µL total reaction volume. Reaction was again incubated for 1 h at 37 °C. After the digestion, the samples were purified by solid phase extraction (SPE) on Oasis WAX 1cc columns (Waters #186006343) using Otto positive pressure manifold instrument (Waters). In this step the phosphate containing RNA caps should be retained while most nucleosides removed. Briefly, columns were equilibrated with 1 mL of MeOH:H_2O:formic acid (73:25:2) and 1 mL of H_2O. Sample after digestion was mixed with 800 µL of 50 mM ammonium acetate (pH 4.5) and loaded on the SPE column. Then the column was washed with 1 mL H_2O and 1 mL of 50% MeOH and caps were eluted with 2× 200 µL of ACN:H_2O:10% ammonia solution (73:25:2). Samples were concentrated in vacuo and re-dissolved in 15 µL of 50 mM ammonium acetate. Control samples were going through same protocol without the addition of Nuclease P1 enzyme.

Digestion of pull-down RNA

RNA obtained from pull-down (1-2 µg) was concentrated to approximately 10 µL on 10 kDa cut-off filters Vivacon 500 (Sartorius #VN01H02), mixed with 1.25 µL 500 mM ammonium acetate (pH 5.5), 30 U NuP1 and incubated for 1 h at 37°C. Right after 2 µL of 500 mM ammonium acetate (pH 9.1), 0.1 U rSAP, 2 µL of ^{15}N-Ap_2A and ^{15}N-Ap_2G (both 1 nM) mix, molecular biology grade water (up to 20 µL final volume) was added to the reaction and incubated for 1 h at 37°C. Reaction was transferred to HPLC vial and analysed by HILIC LC-MS method.

HILIC LC-MS

HPLC system (Acquity Premier, Waters) equipped with an Acquity Premier BEH Amide VanGuard FIT column (1.7 µm, 2.1 mm × 150 mm, Waters) was used. The column temperature was 30°C. Mobile phase A contained 15 mM ammonium acetate in a 90:10 v/v mixture of acetonitrile (Optima, Fisher) with ultrapure water (18.2 MΩ.cm, Purelab Chorus system, Elga). Mobile phase B contained 15 mM ammonium acetate in ultrapure water. The autosampler was kept at 10°C. The sample injection volume was 5 µL with 35 µL of acetonitrile as a pre-plug (auto-addition tool in autosampler). Mass spectrometer (Xevo TQ Absolute, Waters) equipped with an electrospray ionization source was used with multiple-reaction monitoring method (MRM). Gradient of separation, ESI parameters and MRM transitions are stated in Table S1-S4. The Ap_2A and Ap_2G quantitation was done from 3 biological replicates and expressed as a mean of fmol of Ap_2A and Ap_2G per 100 µg of RNA.

In vitro transcription of 35-mer RNA with T7 RNA polymerase (RNAP)

In vitro transcription was performed using a modification of a previously published method (2) in a 50 µL mixture containing: 33 µM template DNA (Table S5), 1 mM UTP, 1 mM CTP, 1 mM ATP, 0.8 mM GTP and 0.5 µL $\alpha^{32}P$ GTP (activity: 9.25 MBq in 25 µL), 1.6 mM of Np_nNs (NAD, Ap_2A, m^7Gp_3A, m^7Gp_3AmG (CleanCap reagent AG, Trilink #N-7113) or ADPRibose), 5% DMSO, 0.12% triton X-100, 12 mM DTT, 4.8 mM magnesium chloride and 10× reaction buffer

for T7 RNAP (40 mM Tris-HCl, 6 mM $MgCl_2$, 1 mM DTT, 2 mM spermidine, pH 7.9 at 25 °C) and 62.5 units of T7 RNAP. The mixture was incubated for 2 h at 37 °C.

In vitro transcription of U1 RNA with T7 RNA polymerase (RNAP)

The DNA template for human U1 snRNA (Table S5, S6) was prepared by PCR from the plasmid U1–SP65 (kind gift from David Stanek lab, Institute of Molecular Genetics AS CR) with T7-flanked forward primer. The reaction mix consisted of DreamTaq buffer (1x, Thermo Fisher Scientific), DreamTaq DNA polymerase (1.25 U, Thermo Fisher Scientific), forward and reverse primer (0.5 µM, Table S5), 2 mM dNTPS and 40 ng plasmid template. PCR was conducted with the following program: initial denaturation at 95 °C for 2 min, followed by 30 cycles of 95 °C for 30 s, 60 °C for 30 s and 72 °C 1 min, the final extension was performed at 72 °C for 5 min. The PCR product was purified using Monarch® PCR & DNA Cleanup Kit (NEB). The T7 in vitro transcription was performed as described above for 35-mer except for using 300 ng U1-PCR template per 25 µL IVT reaction.

DNAse I treatment

The resulting IVT mixture was digested by DNAse I (NEB) at 37 °C for 1 hour, according to manufacturers' instructions. The enzyme was heat deactivated at 75 °C for 10 min followed by purification on RNA Micro Bio-Spin™ P-30 (Bio-Rad #7326250) or Zymo RNA Clean & Concentrator-5 Kit (Zymo #R1014) for further use.

RNA 5′-polyphosphatase treatment

The products from the previous reaction were treated with 20 units of 5′-polyphosphatase (Epicentre RP8092H) for 1 h at 37 °C. All samples were purified on RNA Micro Bio-Spin™ P-30 (Bio-Rad #7326250 or Zymo Clean RNA Clean & Concentrator Kit (Zymo #R1014) for further use.

Terminator™ 5′-phosphate-dependent exonuclease treatment

The RNA was treated with 1 unit of Terminator™ 5′-phosphate-dependent exonuclease (Epicentre #TER51020) in reaction buffer A (supplied with the enzyme) at 30 °C for 1 hour. All samples were purified on Micro Bio-Spin™ P-30, or Zymo Clean RNA Clean & Concentrator-5 Kit and concentration was measured using Nanodrop One (ThermoFisher) or Qubit (ThermoFisher).

DXO and NUDT12 expression and purification

Recombinant mouse DXO was expressed and purified as described before (3). The plasmid for the expression of recombinant mouse NUDT12 was transformed into *E. coli* BL21 (DE3) cells and one liter of culture was grown at 37 °C in LB medium containing 0.05 mg/ml Kanamycin until the OD_{600} reached 0.5. The culture was adjusted to 0.5 mM isopropyl β-D-thiogalactoside (IPTG) and the incubation continued at 20 °C for 20 h. The cells were harvested by centrifugation at 4000 × g for 15 min and the pellets were stored at −70 °C. All subsequent procedures were performed at 4° C. The pellet was resuspended in 50 mL of lysis buffer (50 mM sodium phosphate buffer pH 8, 150 mM NaCl and 10% glycerol) containing one tablet of proteases inhibitor SIGMAFAST™ (Merck) and incubated with lysozyme 1 mg/ml

for 1 h at 4 °C. The cell lysis was completed by sonication, and the lysate was centrifuged at 20 000 × g for 45 min at 4 °C. The soluble fraction was load onto ÄKTA Pure equipped with HisTrap™ FF Crude column, previously equilibrated with HisTrap buffer 1 (50 mM sodium phosphate pH 8, 200 mM NaCl, 20 mM imidazole and 10% glycerol). Elution of the protein was achieved using a linear gradient of imidazole from 20 to 500 mM. The protein was further purified by size-exclusion chromatography (SEC) using HiLoad 16/600 S75 column and SEC buffer (50 mM Tris-HCl pH 8, 150 mM NaCl, 5 mM DTT). The purified protein was concentrated using Amicon Ultra filters with 3 kDa cut-off and concentration was determined using DC™ Protein Assay Kit II with BSA as a standard.

RLIG1 expression and purification

Coding sequence for human RLIG1 was amplified from HEK293T cDNA using RLIG1 Fwd and RLIG1 Rev cloning primers (Table S5), cleaved by restriction enzymes AseI and XhoI (both from NEB), and cloned into pET28a plasmid as a construct with N-terminal 6×His-tag. The plasmid was then transformed into *E. coli* BL21 (DE3) cells and one liter of culture was grown at 37 °C in LB medium containing 0.05 mg/ml Kanamycin until the OD_{600} reached 0.5. The culture was adjusted to 1 mM IPTG and the incubation continued at 18 °C for 20h. The cells were harvested by centrifugation at 5000 × g for 20 min and the pellets were stored at −80 °C. All subsequent procedures were performed at 4 °C. The pellet was resuspended in 30 mL of lysis buffer (50 mM sodium phosphate buffer pH 8, 300 mM NaCl, 20 mM imidazole, 3 mM β-mercaptoethanol, 1 mM phenylmethylsulphonyl fluoride, and 5% glycerol) supplemented with proteases inhibitor cocktail (Pierce™, ThermoFisher Scientific), the cells were lysed by sonication, and centrifuged at 7120 × g for 20 min at 4 °C. The soluble extract was load onto Äkta Pure equipped with HisTrap™ FF Crude column, previously equilibrated with HisTrap buffer 2 (50 mM sodium phosphate buffer pH 8, 300 mM NaCl, 20 mM imidazole, 3 mM β-mercaptoethanol, and 5% glycerol). After washing out the unbound proteins, the RLIG1 was eluted using a linear gradient of imidazole from 20 to 500 mM. The partially purified RLIG1 was concentrated using Amicon Ultra filters with 10 kDa cut-off and concentration was determined using DC™ Protein Assay Kit II with BSA as a standard. 4.8 mg of RLIG1 were incubated with 25 U of bovine plasma thrombin (Merck) for 4.5 h at 22 °C in order to cleave the N-terminal 6×His-tag. The reaction mixture was 5 times diluted with HiTrap Q buffer (50 mM TrisHCl pH 8 and 5% glycerol) and further purified by anion exchange chromatogrphy on ÄKTA Pure using HiTrap Q HP column (Cytiva). Bound protein was eluted using a linear gradient of NaCl from 0 to 1 M, concentrated using Amicon Ultra filters with 10 kDa cut-off and glycerol concentration was adjusted to 30%. RLIG1 concentration was determined using DC™ Protein Assay Kit II with BSA as a standard.

NUDT12 and DXO decapping activity on RNA

The decapping assay was performed at 37 °C in 10 µL reaction. The reaction mixture consisted of 100 ng of the pure $\alpha^{32}P$ GTP radiolabeled RNA (*in vitro* transcribed, DNAse I and treated, and purified on RNA mini–Quick Spin Columns), 1× decapping buffer (10 mM TrisHCl pH 7.5, 100 mM KCl, 2 mM $MgCl_2$, 2 mM $MnCl_2$, and 2 mM DTT), and 100 nM of the recombinant DXO or 1 µM NUDT12. Water was used as a negative control. The mixtures were incubated at 37 °C for 0, 10, 30, 60, 120, and 240 min and the reaction was stopped by adding 10 µL of 2× RNA loading dye (NEB #B0363S). 18 µL of the mixture were separated on 12.5% PAGE (600 V, 3.5 h), the gels were imaged on Typhoon FLA 9500 system and analyzed using ImageJ software (4).

RNA capping by RLIG1

10 ng of 5'-monophosphate α^{32}P GTP radiolabeled RNA (*in vitro* transcribed, DNAse I and 5′-polyphosphatase treated, and purified with Zymo Clean RNA Clean & Concentrator-5) starting with A (pA-RNA) of G (pG-RNA) were incubated with 0.5 or 1 µM RLIG1 in a mixture containing 50 mM Tris acetate (pH 7), 5 mM $MgCl_2$, 1 mM DTT, 0.2 mM ATP, and 4 U/µL Murine RNase inhibitor (NEB # M0314S). Reaction mixture at final volume of 65 µL was incubated at 37 °C and at 0, 1, 2, 3, 4, and 5 h 10 µL aliquots were taken and the reaction was stopped by adding 10 µL of 2× RNA loading dye. Samples were analyzed on 12.5% PAGE as described above.

The same reaction for both, pA-RNA and pG-RNA, with 0.5 µM RLIG1 was repeated using non-radiolabeled RNA in order to confirm Ap$_2$N cap formation. After 5-h incubation at 37 °C RNA was digested by NuP1 and rSAP and analyzed by LC-MS as described above. Samples without addition of RLIG1 or NuP1 were used as negative controls.

Western Blot

For Western Blot analysis, control and menadione-treated Expi293F cells were harvested by centrifugation at 1200 RPM for 5 minutes and proteins were extracted using RIPA Lysis and Extraction Buffer (Thermo Fisher Scientific #89900) containing 1× protease inhibitors (EDTA-free Protease Inhibitor Cocktail, Sigma Aldrich #04693116001). The protein concentration of the lysate was then measured using DC™ Protein Assay Kit II (Bio-Rad #5000112) on Tecan (Schoeller). Proteins were then loaded on 10-well protein gels (Bio-Rad, Mini-PROTEAN TGX) and separated at 120V (270 µg for RLIG1, DXO and NUDT12, and 54 µg for GAPDH). The transfer of the proteins onto PVDF membranes (Thermo Fisher Scientific) was performed at 30V for 2 h. All primary antibodies were incubated with the membrane overnight at 4 °C. For RLIG1 we used 1:200 dilution of mouse C12orf29 (D-9) monoclonal antibody (Santa Cruz Biotechnology, Inc,#sc-390730); for NUDT12 we used 1:500 dilution of rabbit Anti-NUDT12 antibody (Abcam #ab197310); for DXO we used 1:500 dilution of rabbit DOM3Z Polyclonal antibody (Proteintech Europe #11015-2-AP); and for GAPDH we used 1:5000 dilution of rabbit GAPDH monoclonal antibody (14C10) (Cell Signaling Technology #2118). Secondary antibody was added at a dilution of 1:10000 for 1 hour at room temperature (Peroxidase conjugated AffiniPure Goat Anti Rabbit IgG, Jackson Immuno research #111-053-003). Blots were then developed using SuperSignal™ West Femto Maximum Sensitivity Substrate (Thermo Fisher Scientific #34096) and visualized using Azure Biosystems (model c600).

Proteomic sample preparation

Approximately 1 million pelleted cells were lysed in 250 µl freshly prepared, preheated (95 °C) 4% deoxycholate, 100 mM TEAB buffer pH 8.5. The samples were ultrasonicated using a probe (Bandelin Sonoplus) for 30 s at 70% intensity (1s ON, 1s OFF cycles) and heated at 95 °C for 5 min and then diluted to total volume of 500 ul using 100 mM TEAB buffer. Protein content was assessed using Bicinchoninic acid (BCA) protein assay kit (Pierce, Thermo Scientific) according to manufacturer's instructions. Then equivalent of 10 µg protein content from each sample was reduced using 10 mM tris(2-carboxyethyl)phosphine (TCEP) and alkylated with 25 mM chloroacetamide at 95 °C for 5 min.

The reduced and alkylated protein samples were subjected to modified R2-P1 protocol using automation with KingFisher Flex (5) scaled down to 10 µg protein input. Briefly, 2 µl of bead suspension (mixture of 1:1 SpeedBeads™ magnetic carboxylate modified particles, GE Life

Sciences, cat. num. 45152105050250 and 45152105050250) was added to each sample and twice 0.25 µg LysC/trypsin (total 1:20 enzyme:protein ratio, Thermo Scientific) for overnight digestion.

Proteomic LC-MS/MS method

Digested peptides were separated and analyzed by LC-MS/MS. LC separation was performed on Vanquish Neo HPLC system (Thermo Scientific). Samples were loaded onto the trap column (C18 PepMap100, 5 µm particle size, 300 µm × 5 mm, Thermo Scientific) under combined control (max flow 100 µl/min or pressure of 800 bar) in Trap and Elute mode. Loading buffer was composed of water, 2% acetonitrile and 0.1% formic acid. Mobile phase A was composed of water and 0.1% formic acid, mobile phase B was composed of acetonitrile and 0.1% formic acid. Peptides were eluted with mobile phase B gradient from 2% to 35% B for 32 min. Nano reversed phase column (Aurora Ultimate TS, 25 cm × 75 µm ID, 1.7 µm particle size, Ion Opticks) was used for LC-MS analysis operated at 0.4 µl/min under separation conditions.

Separated peptide mixture was analyzed online by Orbitrap Astral Zoom MS instrument (Thermo Scientific) by data independent approach. Eluting peptide cations were converted to gas-phase ions by electrospray ionization in positive mode. Spray voltage was set to 1600 V and ion transfer tube temperature to 280 °C. MS1 scans of peptide precursors were analyzed in Orbitrap in range 380-980 m/z at 120k resolution and with following settings: RF Lens 40%, maximum injection time 3 ms, normalized AGC target 500%. DIA scans were performed in Astral with 2 Da isolation window. Normalized AGC target was set to 500% and maximum injection time 3 ms with pre-accumulation switched on. MS2 scan mass range was 150-2000 m/z. HCD collision energy was set to 25%.

Proteomic LC-MS data processing

The acquired dataset was analyzed using Spectronaut version 20.2.250922.92449 (6). The analysis workflow employed was directDIA+(Deep) with normalization. The analysis was performed using a human proteome database (one protein per gene), which was downloaded on 12th May 2025 (uniprot.org) along with common contaminants. Search parameters were as follows: trypsin enzyme specificity, with Carbamidomethyl (C) as a fixed modification, and Acetyl (protein N-term) and Oxidation (M) as variable modifications. The thresholds for Peptide Spectrum Match (PSM) and protein False Discovery Rate (FDR) were set to 0.01.

Proteomic LC-MS Statistical data analysis

Differential protein expression analysis was performed using the DEP2 package (v0.5.28.2) in R. Protein identifications were filtered based on missing value thresholds (thr parameter was set to 0, missnum = 0), i.e. only proteins quantified in all samples were considered. For statistical testing, a linear model with empirical Bayes moderation was applied using the limma framework to calculate moderated t-statistics and corresponding p-values. To correct for multiple hypothesis testing, adjusted p-values (q-values) were calculated using Storey's method, estimating the false discovery rate (FDR). Proteins with a q-value < 0.05 and fold change <-2, >2 were considered significantly differentially expressed.

RT-qPCR for NUDT12, DXO and RLIG1 enzyme expression in Expi293F cells

The cDNA preparation was performed using reverse transcription from 500 ng of long RNA, control or menadione-treated, using LunaScript® RT SuperMix Kit (NEB #E3010) in 20 µL reaction volume according to manufacturer's instructions. The cDNA was then diluted by water to a volume of 50 µL and used for qPCR. The experiments were performed in technical triplicates on the LightCycler-® 480 Instrument II (Roche) using the Luna® Universal qPCR Master Mix (NEB #M3003) with 2 µL cDNA per well. The program was as follows: Initial denaturation 1 min at 95 °C, 45 cycles of 95 °C for 15 seconds followed by annealing/extension at 55 °C for 20 seconds. The Cq were called using the Abs Quant/2nd Derivative Max method in the LightCycler -® 480 software. The sequences of the primers used for qPCR are given in Table S5.

T4 RNA ligase 2 truncated K227Q reaction with 35-mer capped RNA and U1 capped RNA

1 µM of in vitro transcribed RNA (with 5'-triphosphate, monophosphate or capped with NAD, Ap_2A, m^7Gp_3A, or ADPRibose) was incubated with 1 µL (200 U) of T4 RNA ligase 2 truncated K227Q (NEB #M0351), 4 µL of 50% PEG8000, and 3 µM of RNA adaptor (Table S5) in 10× T4 RNA Ligase Reaction Buffer provided with the enzyme. The ligation reaction was performed over-night at 16 °C in 20 µL followed by purification on RNA Micro Bio-Spin™ P-30. Sample without T4 RNA ligase 2 was used as a negative control. Then 10 µL of the purified samples were mixed with 2× RNA loading dye and analyzed by 12% PAGE (600 V, 3.5 h). Typhoon FLA 9500 was used as a visualization imaging system.

Ap_2N-RNA Seq protocol

1. NUDT12 Treatment

As starting material, 5 µg of the short RNA fraction (or 1 µg in vitro synthetized U1 RNA capped with pppA, NAD, Ap_2A, m^7Gp_3A, or ADPRibose) was used. The RNA was incubated at 37 °C for 1 hour in 1× decapping buffer (10 mM TrisHCl pH 8.5, 100 mM KCl, 2 mM DTT, 2 mM $MgCl_2$ and 2 mM $MnCl_2$) with the addition of NUDT12 (final concentration 5 µM, Negative control with Ap_2N moiety cleaved off) or without NUDT12 (Ap_2N samples) in a total volume of 10 µL. The samples were then purified using Zymo Clean and Concentrator 5 (Zymo Research #D4014) and eluted in 6 µL water.

2. mRNA Decapping enzyme (MDE) treatment

Subsequently, all samples were treated with mRNA decapping enzyme (MDE, NEB #M0608) in 10 µL reactions at 37 °C for 1 hour under conditions provided by the manufacturer. After the reaction, samples were purified using Zymo RNA Clean & Concentrator and eluted in 6 µL water.

3. *Terminator^{TM} 5'-Phosphate-Dependent Exonuclease treatment*

Next, the samples were treated in a total volume of 10 µL at 30 °C for 1.5 hours with Terminator Exonuclease (Lucigen #TER51020) using Buffer A provided by the supplier. After the reaction, samples were purified using Zymo RNA Clean & Concentrator and eluted in 6 µL water.

4. E. coli Poly(A) Polymerase treatment and blinding the 3' end

To extend the RNA by a polyA tail for subsequent reverse transcription, samples were treated for 1h at 37 °C with *E. coli* Poly(A) Polymerase (NEB #M0276) using a 3:7 ratio of ATP and

Cordycepin (Merck #C9137-1MG). Cordycepin was used to blind the 3'OH group and avoid self-ligation in the next step. After the polyadenylation, samples were purified using Zymo RNA Clean & Concentrator and eluted in 10 µL water.

5. Ligation of the 5' adaptor by the T4 RNA ligase 2 truncated K227Q

The last step of the protocol is the ligation of the RNA to the 5' adaptor (Table S5) using T4 RNA ligase 2 truncated K227Q treatment. The full volume of the RNA from the previous step (polyadenylation) was combined with 1 µL (200 U) of T4 RNA ligase 2 truncated K227Q (NEB, M0351), 4 µL of 50% PEG8000, 3 µL of 20 nM (final 3 nM) of 5' Ap_2N seq adaptor (Table S5) and 2 µL T4 RNA Ligase Reaction Buffer in a total volume of 20 µL. For the U1 *in vitro* synthetized sample the adaptor concentration was 1000× higher (3 µM final). The ligation reaction was performed over-night at 16 °C followed by purification by Zymo RNA Clean & Concentrator and eluted in 10 µL water.

6. Reverse transcription of ligated RNA

cDNA was prepared using SuperScrip^{TM}IV First-Strand Synthesis System (ThermoFisher Scientific #18091050) using 1 µL of 50 µM 3' RACE Adaptor primer (Table S5) and 10 µL of the purified RNA after the ligation step in a total volume of 20 µL. The cDNA was then used for qPCR, Sanger sequencing or RNA sequencing.

7. Quality control of the protocol by Ap_2A-U1, m^7Gp_3A-U1, ppp-U1

To assess the enrichment between Ap_2A (+/- NUDT12 treatment) and other caps, 10 µL of the cDNA prepared from the U1 capped with pppA, Ap_2A and m^7Gp_3A was diluted with water to 50 µL. Next 2 µL per well were used for qPCR with U1 Fwd/Rev primers (Table S5) using the Luna® Universal qPCR Master Mix (NEB #M3003) with initial denaturation 1 min at 95 °C, 45 cycles of 95 °C for 15 seconds followed by annealing/extension at 55 °C for 20 seconds. The Cq were called using the Abs Quant/2nd Derivative Max method in the LightCycler® 480 software.

8. Final PCR for Illumina RNA sequencing

For the enrichment of the final product, 10 µL of the cDNA were used for PCR reaction using Phusion® High-Fidelity DNA Polymerase (NEB #M0530). The sequences of the primers used for PCR are given in Table S5. The thermal cycling conditions were composed of initial denaturation step at 95 °C for 30 seconds, 30 cycles of 95 °C for 30 seconds, 63 °C for 30 seconds, followed by final extension 72 °C for 1 minute. All samples were purified using Agencourt® RNACleanTM XP (Beckman Coulter #A66514). Samples were analyzed on Agilent TapeStation and the concentration was measured using Qubit and Nanodrop.

9. Illumina sequencing

The Ap_2N-RNA Seq samples after the PCR step were performed commercially at Novogene (Cambridge, UK), using standard dsDNA 2x150 bp paired-end sequencing at the NovaSeq platform providing ~ 40 Mr per sample. RNA-seq including both, the library preparations and Illumina sequencing were performed commercially at Novogene (Cambridge, UK). The mRNA libraries were prepared following standard Illumina protocol with poly(A)-selection, libraries from small RNA fraction sizing 20 - 150 nt were processed according to the miRNA protocol while skipping size-selection. The libraries were sequenced for 2x150 bp paired-end reads at the NovaSeq 6000 platform providing ~20 Mr per sample in case of mRNA and ~50 Mr per sample in case of small RNA.

10. Bioinformatic analysis of the Ap_2N datasets

The overlapping paired-end reads were merged using fastp (0.20.1) (7) and only reads containing single copy of both adaptors (the 5' Ap_2N-RNA-Seq and the reverse polyT-adaptor, Table S5) in appropriate orientation were kept. The adaptors and homopolymeric stretches and bases of low quality were trimmed using cutadapt (v3.5 with Python 3.10.12) (8). For mapping and counting the reads, a modified pipeline of Hoffmann A et al, 2018 (9) was used (Figure S13). First, the human genome assembly GRCh38.p14 was scanned for tRNA using tRNAscan-SE (version 2.0.9), the detected tRNAs were masked from the genome and used to generate a pre-tRNA reference. The reads were mapped to the artificial genome containing the masked genome supplemented with the pre-tRNA sequences added as extra chromosomes using segemehl-0.3.4 (10) (--evalue 500 --differences 3 --maxinterval 1000 --accuracy 80). Afterwards the reads mapping to the artificial genome and pre-tRNAs were filtered out and the remaining reads were mapped to clusters (clustering based on 100% identity) of mature tRNAs. Only the primary-mapping reads (samtools v1.13 -F2308) (11) were quantified using featureCounts (v2.0.3) (12). The counts from the masked-genome and cluster-remapped reads were merged, normalized to library size and quantified as RPM in Ap_2N (-NUDT12, n=3 for control cells as well as menadione-treated cells) over Negative Control (+NUDT12, n=2 for control cells, due to failed clustering replicate number 2, n=3 for menadione-treated cells) samples (Dataset S3). A set of criteria to increase the specificity of hit identification was applied: i) raw read count >2 for at least 2 replicates for each condition; ii) the standard deviation of the RPM mean across at least two of the three replicated for each condition lower than the mean; iii) the average RPM across conditions >20; iv) the Gencode biotype categories were removed: *"misc_RNA", vault_RNA", “scRNA”, "processed pseudogene", "unprocessed_pseudogene", “Pseudo_tRNA", "scaRNA", "TEC"* (Figure 4A). To visualize the mapped reads on the references, Integrated Genome Viewer (v2.17.2) was used (13).To identify the exact position of the ligated 5' Ap_2N-RNA adaptor on the selected gene hits, the raw reads mapping the tRNA cluster of interest were extracted from the raw sequencing files, and the first nucleotide adjacent to the forward adaptor sequence (allowing 0-20 3' terminal cytidine repeat) was registered for statistics and plotting purposes. Next, after trimming the forward and reverse adaptor sequences, the reads were re-aligned to the tRNA cluster of interest using Burrows-Wheeler aligner (14) (version 0.7.19-r1273) to obtain the position of the first mapping nucleotide.

Bioinformatic analysis of short and long RNA-seq

The quality of raw data was inspected with the FastQC tool followed by qualitative trimming and sequencing adapter removal using the Trimmomatic v0.26 algorithm. The small RNA reads were then processed with an additional and more stringent adapter removal step using the Cutadapt. Trimmed data were mapped on genomic reference hg38 with the STAR aligner v2.7.10 (15) and read abundances were estimated using the featureCounts tool from the R Subread package. Raw expression data and normalized according to the TPM (Transcripts Per Million) method and differential gene expression analyses were performed using the DESeq2 v1.38.3 and edgeR v3.40.2 packages in R.

Pull-down of RNA hits from Ap_2N-RNA Seq

Approximately 190 µg of sRNA isolated from Expi293F were mixed with 1 nmol 3'-biotinylated DNA probe for desired RNA (Table S5) in the annealing buffer (10 mM TrisHCl, pH 7.6, 0.9 M tetramethylammonium chloride, 100 mM EDTA) and incubated for 10 min at 85 °C and 300 RPM. Then the mixture was let slowly cool down to 25 °C (for approx. 90 min) to allow proper

annealing of the probes to the RNA of interest. 20 µl of Streptavidin Sepharose™ High Performance (Cytiva) resin were transferred into Mobicol filter column, washed twice with 100 µl of PBS and once with 100 µl of annealing buffer. The resin was resuspended using annealed mixture, incubated for 10 min at 25 °C and 300 RPM, and the supernatant containing unbound fraction was collected by centrifugation for 30 s at 1000 × g. The resin was washed six times with 100 µl of 10 mM TrisHCl (pH 7.6). Finally, the resin was resuspended in 100 µl of RNase-free water and captured RNA was eluted by incubation for 10 min at 75 °C and 700 RPM followed by centrifugation for 1 min at 2000 × g. The elution step was repeated one more time to increase the RNA yield. Collected elute was then incubated with 1U of DNaseI in 1X DNaseI buffer (NEB) for 30 min at 37 °C to digest co-eluted DNA probes. The reaction mixture was purified with Zymo RNA Clean & Concentrator-5 kit and 100 ng of RNA were resolved by PAGE to verify purity of pull-down samples. Remaining RNA was digested and analysed by LC-MS as described before.

Verification of $tRNA^{Ala\text{-}TGC}$ and $tRNA^{Leu\text{-}AAG}$ ligation by Sanger sequencing

As a positive control, the capped A23-A72 RNA fragment was *in vitro* synthetized from a DNA template (Table S5) with Ap_2A cap in the reaction mixture. This positive control RNA and short RNA sample from Expi293F cells were processed by the Ap_2N-RNA Seq protocol (+ and – NUDT12 conditions, see above) until the truncated T4 RNA ligase step. The RNA ligated to the adaptor was purified using Zymo Clean and Concentrator-5. The RNA was reverse-transcribed using the Ala-TGC reverse primer (Table S5) and amplified using 40 cycles of PCR with Luna® Universal One-Step RT-qPCR Kit (NEB # E3005). The resulting mixture was separated on a 3% agarose gel electrophoresis, and the bands of the expected sizes (Fig S14 C) were extracted from the gel, cloned into pJET1.2 using the CloneJET PCR Cloning Kit (ThermoFisher # K1231) and Sanger-sequenced (Fig S14 D) with T7 Fwd primer.

For the Sanger sequencing using the SuperScript IV reverse transcriptase, a different 5' RNA adaptor and corresponding Fwd PCR amplification primer (Table S5) to exclude possible PCR amplification artefacts. The sRNA samples were treated as in the Ap2N-sequencing protocol (see above) up to the T4 RNA ligase step. The RNA ligated to the adaptor was purified using Zymo Clean and Concentrator-5. The reverse transcription was primed either by Ala-TGC or Leu-AAG reverse primers using SuperScript IV (ThermoFisher Scientific #18091050) and amplified using 40 cycles of PCR with Phusion™ High-Fidelity DNA Polymerase (NEB #M0530). The resulting mixture was separated on a 2.5% agarose gel electrophoresis, and the bands of the expected sizes were extracted from the gel, cloned into pJET1.2 using the CloneJET PCR Cloning Kit (ThermoFisher # K1231) and Sanger-sequenced with T7 Fwd primer.

ONT Direct RNA sequencing of pull down RNA

500 ng of pull down RNA were deacylated by incubating in 100 mM Tris.Cl pH 9 at 55 °C for 30 minutes. Afterwards, 3M sodium acetate (pH 5.5) was added to a final concentration of 300 mM to neutralize the solution for 5 minutes on ice. The deacylated RNA was purified using Zymo Clean & Concentrator 5. Subsequently, the RNA was polyadenylated using *E. coli* polyA polymerase (NEB) for 30 minutes at 37 °C with final concentration of 100 µM ATP during the polyadenylation step. The polyA RNA was purified using Zymo Clean & Concentrator 5 with two additional washing steps to remove any traces of ATP in the reaction.

The polyadenylated RNA was subjected to ONT direct RNA sequencing on Minion Mk1D device using the RNA004 kit according to manufacturer's instructions (DRS_9195_v4 _revJ_24Nov2025).

ONT Direct RNA sequencing analysis.

The mapping of tRNAs was performed as described in Hoffman et al 2018 (9) and Lucas et al 2023 (16). In short, the human genome GRCh38.p14 genome assembly was used and both nuclear and mitochondrial tRNAs were predicted using tRNAscan-SE 2.0.9. The corresponding pre-tRNA sequences were masked in the genome. From the predicted tRNA sequences, mature tRNA sequences were generated by removing introns, adding CCA to the 3'end and the resulting identical mature tRNA sequences were clustered. The resulting 472 clusters were added as extra chromosomes to the entire genome masked for tRNA sequences and annotated as individual transcripts in the accompanying .gtf file used for counting.

The ONT direct RNA raw data were base-called using the MinKnow Dorado High-accuracy RNA model 130bps and filtered for quality score >=4 and minimal read length 30 nt. To align the filtered reads, the U was converted into T in the basecalled sequences in .fastq files, and Burrows-Wheeler aligner (14) (version 0.7.19-r1273) was used, using the parameters recommended for tRNA ONT RNA004 chemistry (-W13 -k6 -x ont2d -T20) from Lucas et al 2023 (16). Unmapped reads, non-primary and supplementary alignments were removed (samtools -F2308) and the resulting reads were counted using featureCounts fractional counting to account for highly similar tRNA sequences. The resulting counts were summarized at the isodecoder and isoacceptor level.

sRNA purification on PAGE

sRNA (up to 130 µg per lane) was resolved on 10% denaturing PAGE at 600V for 6h. UV-shadowing on fluorescence silica TLC plates was used to temporary visualize RNA bands in gel. Gel portions containing main fractions (A- tRF, B- tRNAs type I, C- tRNAs type II, D, E-U1 and F) were cut as indicated in Fig. S18, and RNA was extracted using "crush and soak" method. Specifically, gel cuts were crushed in 15-ml Falcon tubes and extracted overnight by continuous flipping into 0.3 M sodium acetate buffer (pH 5.2) at 4°C. Buffer containing extracted RNA was separated from gel debris on 0.2-µm centrifugation filters and mixed with isopropanol to final concentration of 70% (V/V), and RNA was precipitated overnight at -20°C. Precipitate was pelleted by centrifugation at 6000 × g and 0°C for 90 min, and pellets were washed once with ice-cold 70% isopropanol (V/V). After short drying on ice, the pellets were dissoved in 80 µl of RNase-free water and digested for subsequent LC-MS analysis.

Digestion of gel excised RNA

RNA excised from the gel (7 µg each A-F band) was digested with the same protocol as for short RNA. After the reaction a small portion (~ 200 ng) was taken aside, further diluted with water, transferred to HPLC vial and analysed by RP C18 LC-MS method for internal modification analysis. The remaining digested RNA was purified with SPE and analyzed by HILIC method as previously described.

RP C18 LC-MS analysis of internal RNA modifications

Acquity Premier HSST3 VanGuard FIT column (1.7 µm, 2.1 mm × 100 mm, Waters) was used for this analysis at 40°C. LC-MS system including mobile phases remains the same as for HILIC method, just the gradient is starting from water (Mobile phase B). Shortly: Flow rate 350 µL/min, 0-1 min 100% B, 10 min 40% B, 10.1 min 20% B, 12 min 20% B, 12.1 min 100% B, 17 min 100% B. The sample injection volume was 2 µL (around 3 ng of digested RNA in injection). ESI parameters were the same and MRM transitions of m^{1}G and m^{2}G were 298>166 and m^{5}U 259>127. The m^{1}G, m^{2}G and m^{5}U relative quantification is shown as area under curve (AUC) per 1000 canonical nucleotides.

Chemical synthesis

General remarks for the synthetic part

^{1}H and ^{31}P NMR spectra were acquired on a Bruker AVANCE IIIHD 500 (^{1}H at 500.0 MHz, ^{13}C at 125.7 MHz, ^{31}P at 202.4 MHz). Coupling constants (*J*) are reported in Hz with the following splitting abbreviations: s = singlet, d= doublet, t = triplet, q = quartet, m = multiplet. NMR spectra were measured in D_2O. Reactions were monitored by thin layer chromatography (TLC) on TLC Silica gel 60 F254 (Merck) using IPAV (isopropyl alcohol/NH_4OH/H_2O, ratio 11/7/2) as mobile phase. The water used in synthetic part was of HPLC quality. All materials were purchased from commercial suppliers and used without further purification unless otherwise stated. Purification of nucleoside diphosphates was performed using HPLC Alliance e2695 system equipped with PDA and QDa detectors (Waters) with a 4.6×50 mm XBridge BEH C18 column (Waters). High resolution mass spectra were measured on Xevo G2-XS QTof mass spectrometer (Waters).

Ap$_2$G synthesis and characterization

1. Synthesis of adenosine 5'-monophosphoimidazolide

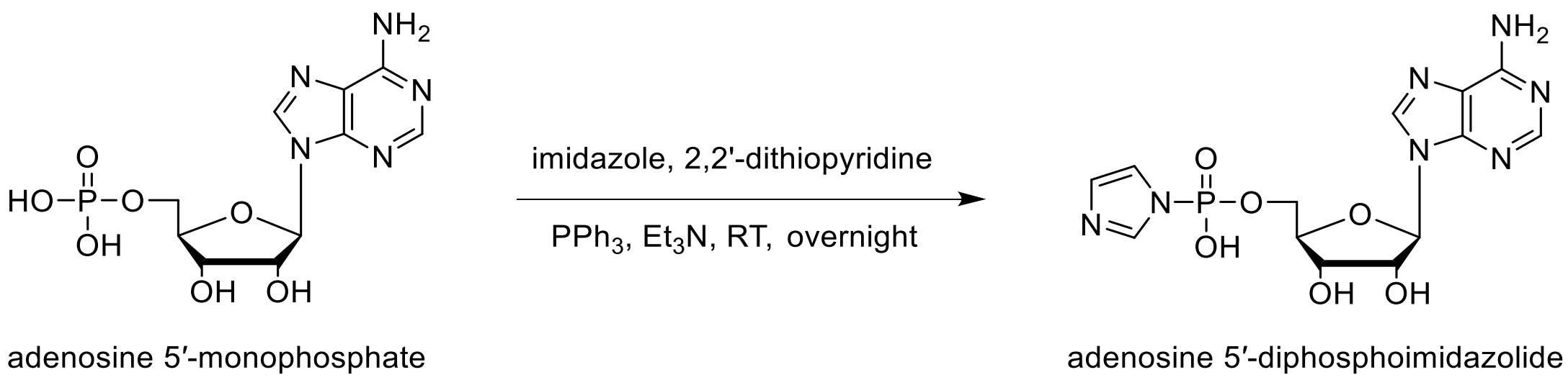

Scheme 1: Synthesis of adenosine 5'-phosphoimidazolide.

Adenosine 5′-monophosphate (1.0 g, 2.88 mmol), imidazole (1.56 g, 23.0 mmol), and 2,2′-dithiopyridine (1.9 g, 8.7 mmol) were suspended in dimethylformamide (DMF; 25 mL). Then, triethylamine (800 µL, 5.8 mmol) and triphenylphosphine (2.26 g, 8.7 mmol) were added. Resulting suspension was stirred at RT for 24 h. The resulting clear solution was poured into a flask containing anhydrous sodium perchlorate (1 eq.) dissolved in dry and cold acetone (8 mL per 1.0 mL of DMF). After cooling at 4 °C for 30 min, liquids were separated from solids by centrifugation. Resulting solid pellet was washed four times with dry and cold acetone and was centrifuged each time. Finally, solid was washed with diethyl ether and centrifuged. Product was isolated as an off-white solid in 70% yield (800 mg, 2.0 mmol). Solid was dried overnight under vacuum and used in the next step without further purification.

^{1}H NMR (401 MHz, DMSO-d6) δ 8.39 (s, 1H), 8.13 (s, 1H), 7.64 (s, 1H), 7.29 (bs, 2H), 7.09 (s, 1H), 6.85 (s, 1H), 5.89 (d, *J* = 6.1 Hz, 1H), 5.52 (bs, 1H), 5.35 (bs, 1H), 4.58 (t, *J* = 5.6 Hz, 1H), 4.03 (dd, *J* = 5.0, 3.0 Hz, 1H), 3.94 (q, *J* = 3.9 Hz, 1H), 3.73 (ddd, *J* = 16.4, 6.7, 4.5 Hz, 2H).

2. Synthesis of P[1]-(5'-adenosyl)-P[2]-(5'-guanosyl) diphosphate (Ap_2G)

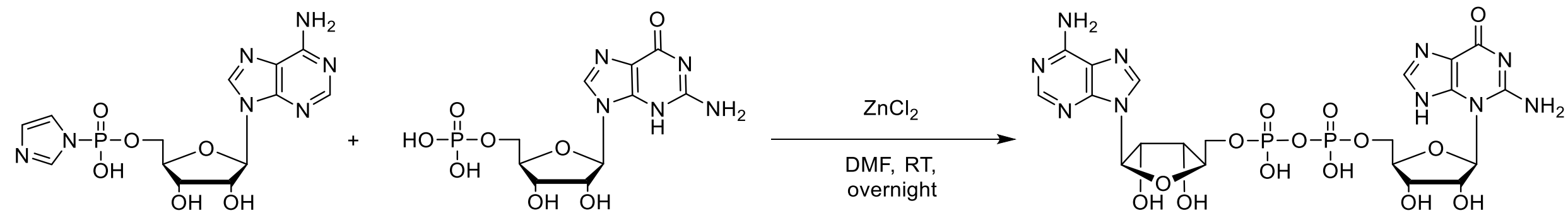

adenosine 5′-diphosphoimidazolide + guanosine 5′-monophosphate → Ap_2G 25% yield

Scheme 2: Synthesis of P[1]-(5'-adenosyl)-P[2]-(5'-guanosyl) diphosphate (Ap_2G).

The guanosine 5′-monophosphate (76 mg, 0.189 mmol), adenosine 5′-phosphoimidazolide (50 mg, 0.125 mmol), and dry $ZnCl_2$ (3 eq.) were suspended in dry DMF (1.0 mL). The resulting suspension was stirred at RT overnight. The reaction was stopped by the addition of H_2O. Products were isolated using preparative HPLC (A—triethylammonium acetate 0.1 M, pH 7, B—acetonitrile). Co-distillations with water followed by several freeze-drying from water gave off-white solid product in 25% yield (0.22 mg, 0.032 mmol).

^{1}H NMR (500 MHz, MeOD+DMSO-d6) δ 8.44 (s, 1H), 8.09 (s, 1H), 7.93 (s, 1H), 5.96 (d, *J* = 5.6 Hz, 1H), 5.71 (d, *J* = 6.0 Hz, 1H), 4.66 (t, *J* = 4.0 Hz, 1H), 4.59 (t, *J* = 4.0 Hz, 1H), 4.37-4.34 (m, 3H), 4.51 – 4.04 (m, 5H), 2.99 (q, *J* = 7.3 Hz, 16H), 1.14 (t, *J* = 7.3 Hz, 24H). ^{13}C NMR (126 MHz, MeOD+DMSO-d6) δ 177.71, 158.77, 156.93, 154.88, 153.69, 152.86, 150.72, 140.68, 138.02, 119.79, 117.62, 88.65, 88.36, 85.11, 75.65, 74.53, 71.83, 71.60, 66.05, 47.07, 9.21. ^{31}P NMR (202.4 MHz, MeOD+DMSO-d6, external ref. H_3PO_4, 0.0 ppm) *δ* −11.56 ppm. MS (ESI^-) *m*/*z* (%): 345(2.5), 691 (100), 692 (20). HRMS (ESI) *m*/*z*: [M^-] ($C_{20}H_{25}O_{14}N_{10}P_2$) calc.: 691.10324, found: 691.10370.

<u>Ap_2G (3'-5')synthesis and characterization</u>

Synthesis of guanosine 5'-monophosphoimidazolide

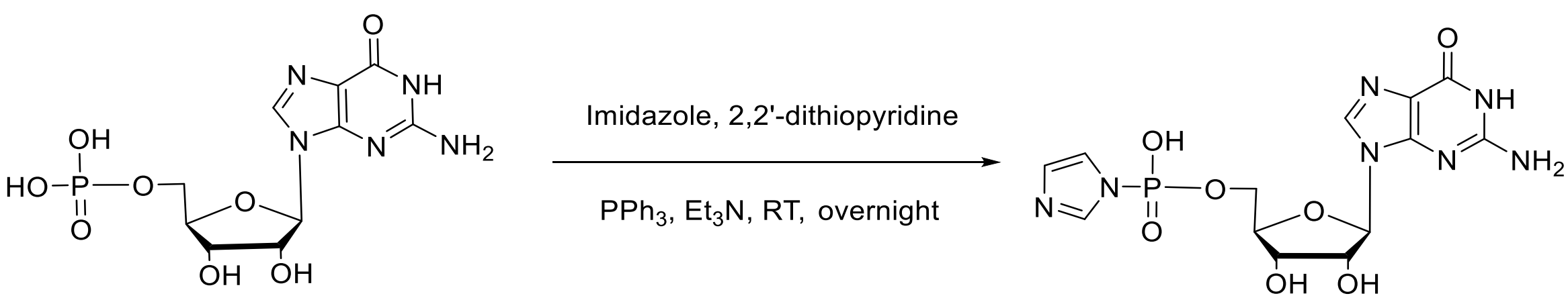

guanosine 5'-monophosphate (**GMP**) → guanosine 5'-monophosphoimidazolide (**ImpG**)

Scheme 3: Synthesis of guanosine-5′-monophosphoimidazolide.

Guanosine 5'-monophosphate (0.274 g, 0.754 mmol), imidazole (0.427 g, 6.27 mmol), and 2,2'-dithiopyridine (0.520 g, 2.36 mmol) were suspended in dimethylformamide

(DMF; 6 mL). Then, trimethylamine (220 µL, 1.58 mmol) and triphenylphosphine (0.62 g, 2.36 mmol) were added. Resulting suspension was stirred at RT for 24 h. The resulting clear solution was poured into a flask containing anhydrous sodium perchlorate (1 eq.) dissolved in dry and cold acetone (8 mL per 1.0 mL of DMF). After cooling at 4 °C for 30 min, liquid phase was separated from solid by centrifugation. The resulting pellet was washed four times with dry, cold acetone and once with diethyl ether, with centrifugation after each wash. The product was isolated as an off-white solid and dried overnight under vacuum. ^{1}H NMR and mass spectrometric analysis indicated that the isolated material consisted of a mixture of guanosine 5'-phosphoimidazolide (ImpG) and residual guanosine 5'-monophosphate (GMP). ESI-MS showed peaks at m/z 412.1 (ImpG) and 362.1 (GMP). The enriched mixture was used directly in the subsequent step without further purification.

Synthesis of P^{1}-(3'-adenosyl)-P^{2}-(5'-guanosyl) diphosphate (Ap$_2$G 3'-5')

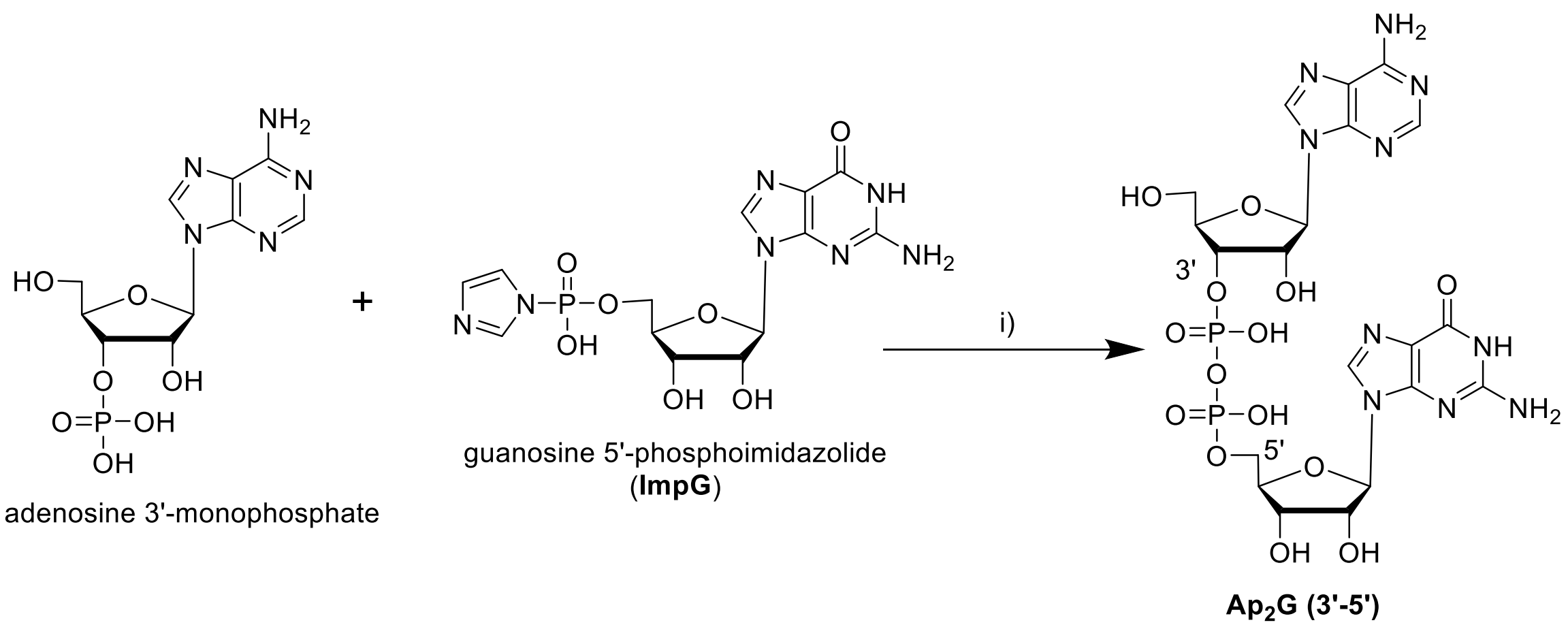

Scheme 4: Synthetic scheme of P^{1}-(5'-adenosyl)-P^{2}-(5'-guanosyl) diphosphate (Ap$_2$G 3'-5' bridge). Conditions i) $MgCl_2$, 50 °C 1.5h.

Guanosine 5'-phosphoimidazolide (2 mg) in 400 µL of 50 mM $MgCl_2$ buffer solution was added to adenosine-3'-monophosphate (3.6 mg). The reaction mixture was incubated for 1.5 h at 50 °C. The desired product **Ap$_2$G** (**3'-5'**) was isolated using HPLC (A—triethylammonium acetate 20 mM, B—triethylammonium acetate 20 mM in 50% acetonitrile pH 7.5). The final product **Ap$_2$G** (**3'-5'**) was freeze-dried from water giving off-white solid product in 4% yield. In order to confirm the chemical structure of product, it was analysed by ^{1}H and ^{31}P NMR and on Xevo G2-XS QTof mass spectrometer (Waters).

Ap$_2$G (3'-5') — ^{1}H NMR (500 MHz, D_2O) δ 8.14 (s, 1H), 8.04 (s, 1H), 7.89 (s, 1H), 5.87 (d, *J* = 5.6 Hz, 1H), 5.70 (d, *J* = 5.1 Hz, 1H), 4.45 (t, *J* = 5.1 Hz, 1H), 4.37 – 4.27 (m, 2H), 4.26 – 4.10 (m, 4H), 3.81 – 3.66 (m, 3H), 3.10 (q, *J* = 7.3 Hz, 34H), 1.81 (s, 8H), 1.18 (t, *J* = 7.3 Hz, 53H). ^{31}P NMR (202 MHz, D_2O) δ -10.19 (d, *J* = 21.5 Hz), -11.24 (d, *J* = 26.5 Hz). HRMS [M-H]$^{-}$ *m*/*z* 691.1032.

Synthesis of isotopically labeled P1-(5'-15N-adenosyl)-P2-(5'-guanosyl) diphosphate (15N-Ap2G) and P1-(5'-15N-adenosyl)-P2-(5'-15N-adenosyl) diphosphate (15N-Ap2A)

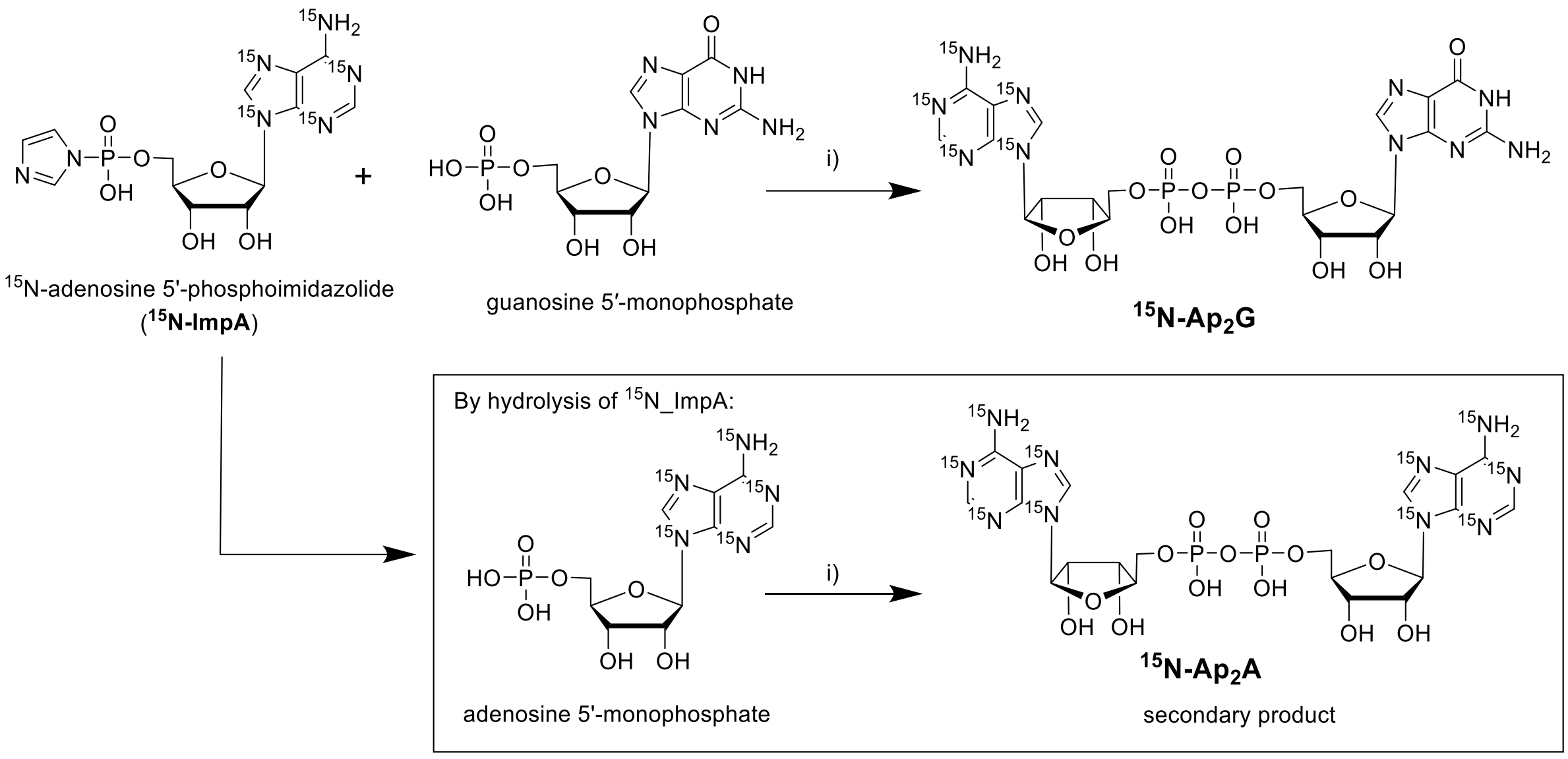

Scheme 5: Synthetic scheme of P1-(5'-15N-adenosyl)-P2-(5'-guanosyl) diphosphate (15N-Ap2G) and P1-(5'-15N-adenosyl)-P2-(5'-15N-adenosyl) diphosphate (15N-Ap2A). Conditions i) $MgCl_2$, 50 °C 3h.

Isotopic labeled ^{15}N-adenosine 5'-phosphoimidazolide (1.5 mg) in 300 µL of 50 mM $MgCl_2$ buffer was added to the desired nucleoside-5'-monophosphate (2.4 mg). The reaction mixture was incubated for 3 h at 50 °C. Ap_2A was formed as a secondary product in the reaction, resulting from partial hydrolysis of ^{15}N-adenosine 5'-phosphoimidazolide to ^{15}N-adenosine 5'-monophosphate, which subsequently reacted with the remaining unhydrolyzed ^{15}N-adenosine 5′-phosphoimidazolide in the mixture. **^{15}N-Ap$_2$A** and **^{15}N-Ap$_2$G** products were isolated using HPLC (A—triethylammonium acetate 20 mM, B—triethylammonium acetate 20 mM in 50% acetonitrile pH 7.5). **^{15}N-Ap$_2$G** needed to be separated three times using HPLC till the compound was sufficiently pure. The final products were freeze-dried from water giving off-white solid product in 9% **^{15}N-Ap$_2$A** and in 7% **^{15}N-Ap$_2$G**. In order to confirm the chemical structure of products, they were analysed by ^1H and ^{31}P NMR and on Xevo G2-XS QTof mass spectrometer (Waters).

^{15}N-Ap$_2$A —^1H NMR (500 MHz, D_2O) δ 8.06 (s, 2H), 7.94 (t, J = 14.6 Hz, 2H), 5.85 (d, J = 5.0 Hz, 2H), 4.50 – 4.07 (m, 13H), 3.11 (d, J = 7.3 Hz, 6H), 1.18 (t, J = 7.4 Hz, 15H). ^{31}P NMR (202 MHz, D2O) δ -10.52. HRMS $[M+H]^+$ m/z 687.0959.

^{15}N-Ap$_2$G — ^1H NMR (500 MHz, D_2O) δ 8.14 (s, 1H), 8.02 (t, J = 14.4 Hz, 1H), 7.77 (s, 1H), 5.89 (d, J = 4.7 Hz, 1H), 5.64 (d, J = 5.3 Hz, 1H), 4.48 (d, J = 5.5 Hz, 2H), 4.33 (d, J = 11.7 Hz, 2H), 4.28 – 4.04 (m, 7H). ^{31}P NMR (202 MHz, D_2O) δ -10.53. HRMS $[M+H]^+$ m/z 698.1013.

Table S1: Gradient of separation for LC-MS analysis of short RNA digest.

Time (min)	Flow (mL/min)	Mobile phase A (%)	Mobile phase B (%)	Curve
0	0.17	85	15	Initial
0.5	0.17	85	15	6
0.6	0.17	70	30	6
6	0.17	70	30	6
6.1	0.17	60	40	6
10.1	0.17	60	40	6
10.2	0.3	50	50	6
14	0.3	50	50	6
14.1	0.3	85	15	6
15	0.4	85	15	6
19.9	0.4	85	15	6
20	0.05	85	15	6

Curve 6 = linear shape of gradient curve

Table S2: Gradient of separation for LC-MS analysis of pull-down RNA digest and free molecules.

Time (min)	Flow (mL/min)	Mobile phase A (%)	Mobile phase B (%)	Curve
0	0.4	90	10	Initial
8	0.4	75	25	6
14	0.4	60	40	6
16	0.4	50	50	6
16.1	0.4	90	10	6
24.9	0.4	90	10	6
25	0.05	90	10	6

Curve 6 = linear shape of gradient curve

Table S3: Mass spectrometry settings.

Parameters	
Ion Mode	Negative
Capillary Voltage (kV)	0.7
Sampling Cone (V)	20
Source Temperature (°C)	140
Desolvation Temperature (°C)	500
Cone Gas (L/h)	150
Desolvation Gas (L/h)	900

Table S4: MRM transitions.

Analyte	Parent	Fragment 1	Fragment 2
Ap_2A	677.1	136.0	250.0
^{15}N-Ap_2A	687.1	141.0	255.0
Ap_2G	693.1	136.0	428.0
^{15}N-Ap_2G	698.1	141.0	433.0

Table SS5: Names and sequences of oligonucleotides and primers used for IVT, cloning and Ap$_2$A-RNA sequencing.

Name	Sequence (5' -> 3')	Comment
**Templates for in vitro synthesis**		
T7 promoter A-starting 35-mer Fwd	<u>CAGTAATACGACTCACTATT</u>**A**GGGAAGCGGGCATGCGGCCAGCCATAGCCGATCA	
T7 promoter A-starting 35-mer Rev	TGATCGGCTATGGCTGGCCGCATGCCCGCTTCCCTAATAGTGAGTCGTATTACTG	
T7 promoter G-starting 35-mer Fwd	<u>CAGTAATACGACTCACTATA</u>**G**GGGAAGCGGGCATGCGGCCAGCCATAGCCGATCA	
T7 promoter G-starting 35-mer Rev	TGATCGGCTATGGCTGGCCGCATGCCCGCTTCCCCTATAGTGAGTCGTATTACTG	
T7-U1-AGG Fwd	<u>TAATACGACTCACTATT</u>**A**GGCTTACCTGGCAGGGGAG	
U1-Rev	CAGGGGAAAGCGCGAACGCAG	
Ala-TGC full length T7 template Fwd	<u>CAGTAATACGACTCACTATT</u>**A**GGGGTGTAGCTCAGTGGTAGAGAGCATGCTTTGCATGTATGAGGTCCCGGGTTCGATCCCCGGCACCTCCA	
Ala-TGC full length T7 template Rev	TGGAGGTGCCGGGGATCGAACCCGGGACCTCATACATGCAAAGCATGCTCTCTACCACTGAGCTACACCCCTAATAGTGAGTCGTATTACTG	
Ala-TGC A23-end T7 template Fwd	<u>CAGTAATACGACTCACTATT</u>**A**GAGCATGCTTTGCATGTATGAGGTCCCGGGTTCGATCCCCGGCACCTCCA	
Ala-TGC A23-end T7 template Rev	TGGAGGTGCCGGGGATCGAACCCGGGACCTCATACATGCAAAGCATGCTCTAATAGTGAGTCGTATTACTG	
**Primers used for molecular cloning**		
RLIG1 Fwd (Ase I)	AGGTATTAATATGAAGCGCTTGGGCTC	
RLIG1 Rev (Xho I)	AGTCTCGAGTTATACATCAAATATTATATCTTTGAGTC	
**Primers for verification of RLIG1 KO clone**		
Fwd gRNA1	GTAGCTGTTCCCATTTTGAGTCTG	
Rev gRNA1	CTAGAAGAGATATCATGATACCAGG	
Fwd gRNA2	TACATCTTCTTATACCACATGGAGC	
Rev gRNA2	TCTTTACTGCAAATAAGAATTGCTGC	
**Adaptors for Ap$_2$N-RNA seq method**		
Ap$_2$N Seq RNA 5' Adaptor	CAAAGCACUUGCACGUCAGCCCCCCCCCCCCCCCCCCC	RNA
3' RACE Adaptor	GCGAGCACAGAATTAATACGACTCACTATAGGT12VN	
5' Adaptor Fwd	CAAAGCACTTGCACGTCAG	For PCR amplification
3' Adaptor Rev	GCGAGCACAGAATTAATACGACT	For PCR amplification

**Sequences used for PCR amplification and Sanger sequencing with SuperScript IV**		
Ap_2N Seq RNA 5' Adaptor Sanger	ACACUCUUUCCCUACACGACGCUCUUCCGAUCUAUGC	RNA
Sanger 5' adaptor Fwd	GACGCTCTTCCGATCTATGC	
Ala-TGC (cl391) Rev	TGGAGGTGCCGGGGATCGAA	
Leu-AAG (cl339) Rev	TGGCAGTGGTGGGATTCGAAC	
**Sequences of oligonucleotides used for RT-qPCR**		
U1 Fwd	CTTACCTGGCAGGGGAGA	
U1 Rev	CTACCACAAATTATGCAGTCGAG	
GAPDH Fwd	GAGTCAACGGATTTGGTCGT	
GAPDH Rev	TTGATTTTGGAGGGATCTCG	
RLIG1 Fwd	GCCATACCTTTGGGCTCGACTA	
RLIG1 Rev	GCTGGTTTGAAGTCCTCCTCAAC	
DXO Fwd	CTTCTGCTCTGTGCTACGCA	
DXO Rev	TGGCCAGGGCTGTGCATC	
NUDT12 Fwd	CTGGTGAAGTCCCGAGAGAG	
NUDT12 Rev	TTGAGCTACAACCCCAGCTT	
ACTB Fwd	CACCAACTGGGACGACAT	
ACTB Rev	ACAGCCTGGATAGCAACG	
tRNA-Ala-TGC Rev	TGGAGGTGCCGGGGATCGAA	
**Antisense probes for RNA pull-down**		
tRNA-Ala-AGC (cluster346)	TGGAGGTGCTGGGGATTGAAC-3'biotin	
tRNA-Leu-AAG (cluster339)	TGGCAGCGGTGGGAT**T**CGAAC-3'biotin<br>TGGCAGCGGTGGGAT**A**CGAAC-3'biotin	
RNU1-1 snRNA	CGCGAACGCAGTCCCCCACTACCAC-3'biotin	

Table S 6: List of reference sequences used in this study (FASTA formated).

```
>Human_U1_IVT_AGG
AGGCTTACCTGGCAGGGGAGATACCATGATCACGAAGGTGGTTTTCCCAGGGCGAGGCTTATCCATTGCACTC
CGGATGTGCTGACCCCTGCGATTTCCCCAAATGTGGGAAACTCGACTGCATAATTTGTGGTAGTGGGGGACTG
CGTTCGCGCTTTCCCCTG
```

The list of tRNA clusters used for tRNA mapping is available in Dataset_S3.xlsx

Table S7: The abundance of tRNA^{Ala} isodecoders in HEK293T cells and brain tissue (17). The most enriched tRNA^{Ala} hits identified by Ap$_2$N-RNA Seq are marked bold.

From Torres et al, PNAS 2019 - Supplementary Table 2							
**tRNA genes**	**log2NormGroup Means.Brain**	**log2NormGroup Means.HEK293T**	**Cluster**	**NormGroup Means.HEK293T**	**% TGC HEK293**	**NormGroup Means.Brain**	**% TGC Brain**
tRNA-Ala-TGC-3	11.70718	14.00219	cluster36	16408.86	66.86	3343.59	46.43
tRNA-Ala-TGC-4-1	11.34031	12.26669	cluster233	4927.66	20.08	2592.83	36.01
**tRNA-Ala-TGC-2-1**	10.26418	10.85951	**cluster259**	**1857.97**	**7.57**	1229.78	17.08
**tRNA-Ala-TGC-5-1**	0.931265	9.985142	**cluster436**	**1013.51**	**4.13**	1.91	0.03
**tRNA-Ala-TGC-1-1**	1.533507	8.119844	**cluster391**	**278.17**	**1.13**	2.89	0.04
					< 0.5 % each of the remaining		
**tRNA genes**	**log2NormGroup Means.Brain**	**log2NormGroup Means.HEK293T**	**Cluster**	**NormGroup Means.HEK293T**	**% AGC HEK293**	**NormGroup Means.Brain**	**% AGC Brain**
tRNA-Ala-AGC-11-1	11.3565	1.32E+01	cluster145	9515.1	27.7	2622.1	12.7
tRNA-Ala-AGC-8	13.63139	13.17749	cluster290	9264.5	27.0	12689.9	61.6
tRNA-Ala-AGC-4-1	11.30618	12.64124	cluster106	6388.4	18.6	2532.2	12.3
**tRNA-Ala-AGC-2**	9.401509	11.10732	**cluster218**	**2206.2**	**6.4**	676.3	3.3
tRNA-Ala-AGC-12	3.310782	1.03E+01	cluster27	1226.4	3.6	9.9	0.0
tRNA-Ala-AGC-13	4.260386	1.02E+01	cluster260	1204.6	3.5	19.2	0.1
tRNA-Ala-AGC-15-1	9.711845	10.20793	cluster333	1182.7	3.4	838.6	4.1
tRNA-Ala-AGC-3-1	9.432143	10.196	cluster350	1173.0	3.4	690.8	3.4
tRNA-Ala-AGC-1-1	2.334859	8.61E+00	cluster49	392.0	1.1	5.0	0.0
**tRNA-Ala-AGC-5-1**	8.882811	8.434779	**cluster346**	**346.0**	**1.0**	472.1	2.3
tRNA-Ala-AGC-chr6-96	2.751309	8.17E+00	cluster127	287.3	0.8	6.7	0.0
tRNA-Ala-AGC-9	3.988496	8.02E+00	cluster15	258.8	0.8	15.9	0.1
tRNA-Ala-AGC-10	2.402269	8.00E+00	cluster370	256.8	0.7	5.3	0.0
tRNA-Ala-AGC-14	2.084645	7.80E+00	cluster152	222.8	0.6	4.2	0.0
					< 0.5 % each of the remaining		

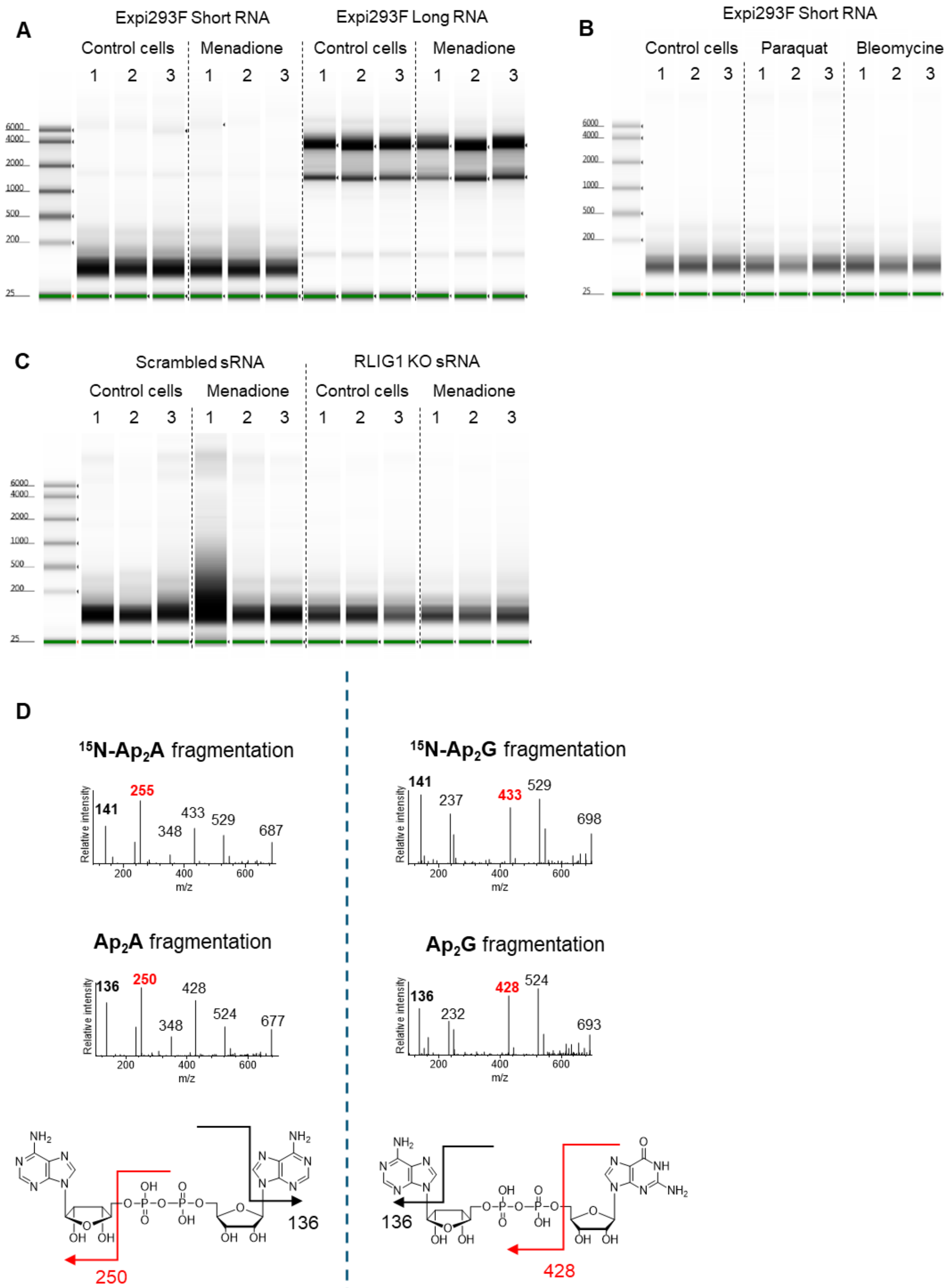

Figure S1: Measurement of Ap$_2$A- and Ap$_2$G-capped RNA. (A-C) Quality assessment of isolated and fractioned RNA from **A)** Expi293F control and menadione-treated cells short and long RNA. **B)** Short RNA isolated from control, paraquat- and bleomycine-treated Expi293F cells. **C)** short RNA isolated from Scrambled control and RLIG1 knock-out Expi293T cells under control condition and treated with menadione. **D)** Collision-induced dissociation (CID) spectra of ^{15}N-Ap$_2$A (m/z 687.1), ^{15}N Ap$_2$G (m/z 698.1), Ap$_2$A (m/z 677.1) and Ap$_2$G (m/z 693.1) standards and a graphical presentation of the two most abundant fragment ions at 26 V collision energy. These fragment ions were selected as the quantifier and qualifier ions for the MRM transitions used throughout the manuscript.

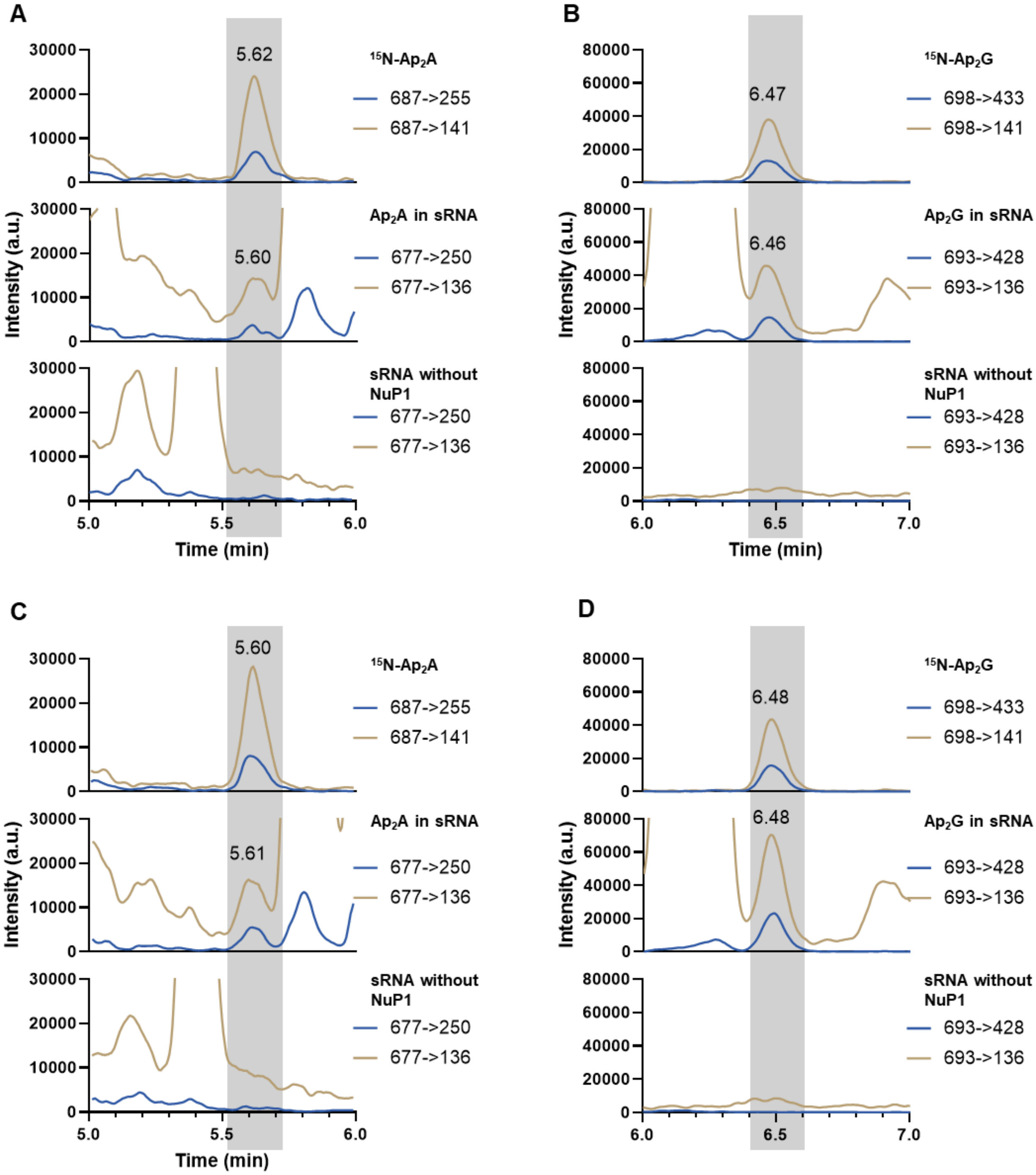

Figure S2: Examples of chromatograms containing all MRM transitions followed for Ap_2A and Ap_2G detection and quantification in sRNA. A) Ap_2A in control cells. **B)** Ap_2G in control cells. **C)** Ap_2A in Menadione-treated Expi293F cells. **D)** Ap_2G in Menadione-treated Expi293F cells

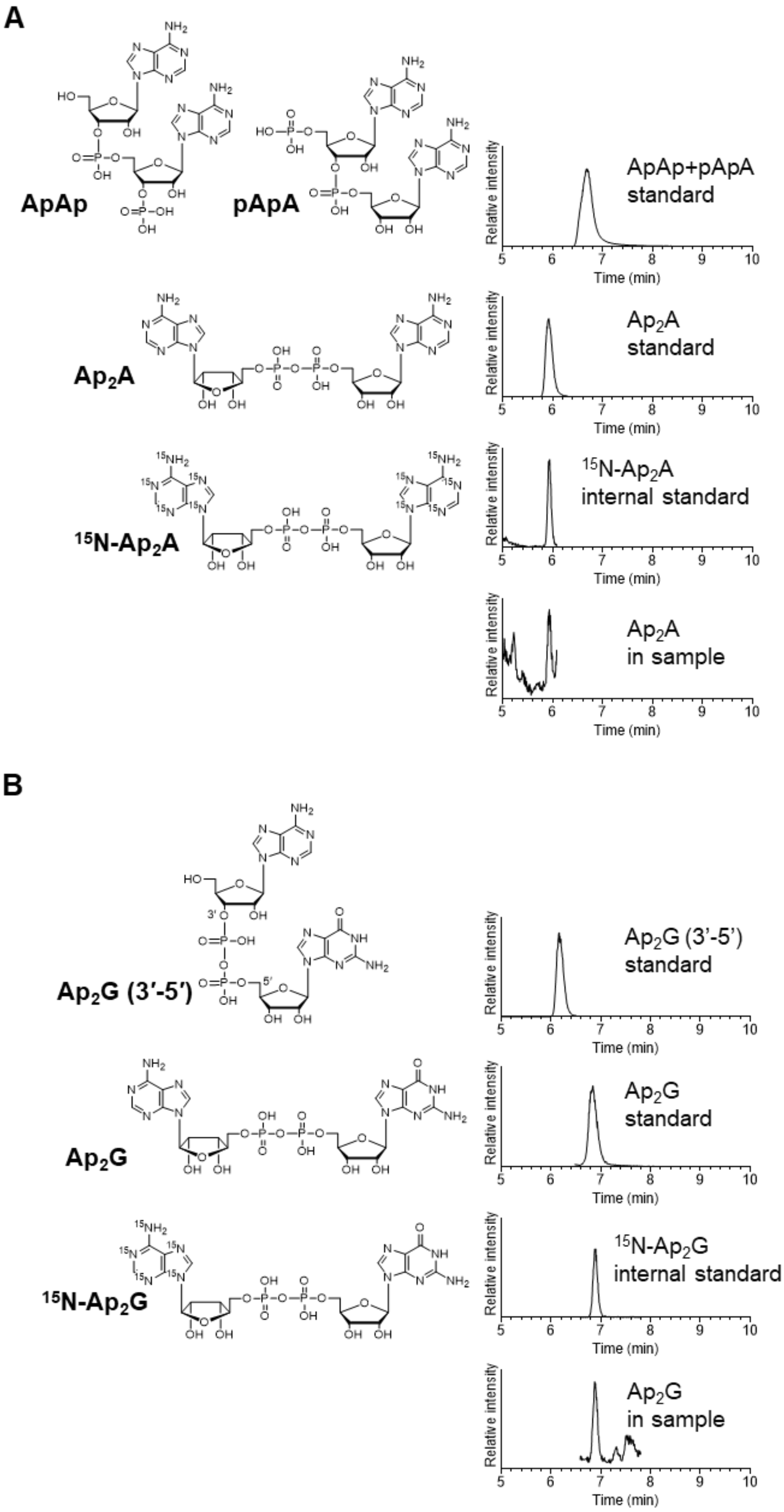

Figure S3: Chemical structures and LC-MS analysis of potential molecules formed after Nuclease P1 digestion of RNA. **A)** Structures (left) and MRM chromatograms (right) of transitions 687->141 (^{15}N-Ap$_2$A), 677->136 (ApAp, pApA and Ap$_2$A. **B)** MRM chromatograms of transitions 698->141 (^{15}N-Ap$_2$G), 693->136 (Ap$_2$G (3′ – 5′) and Ap$_2$G (5′ – 5′)).

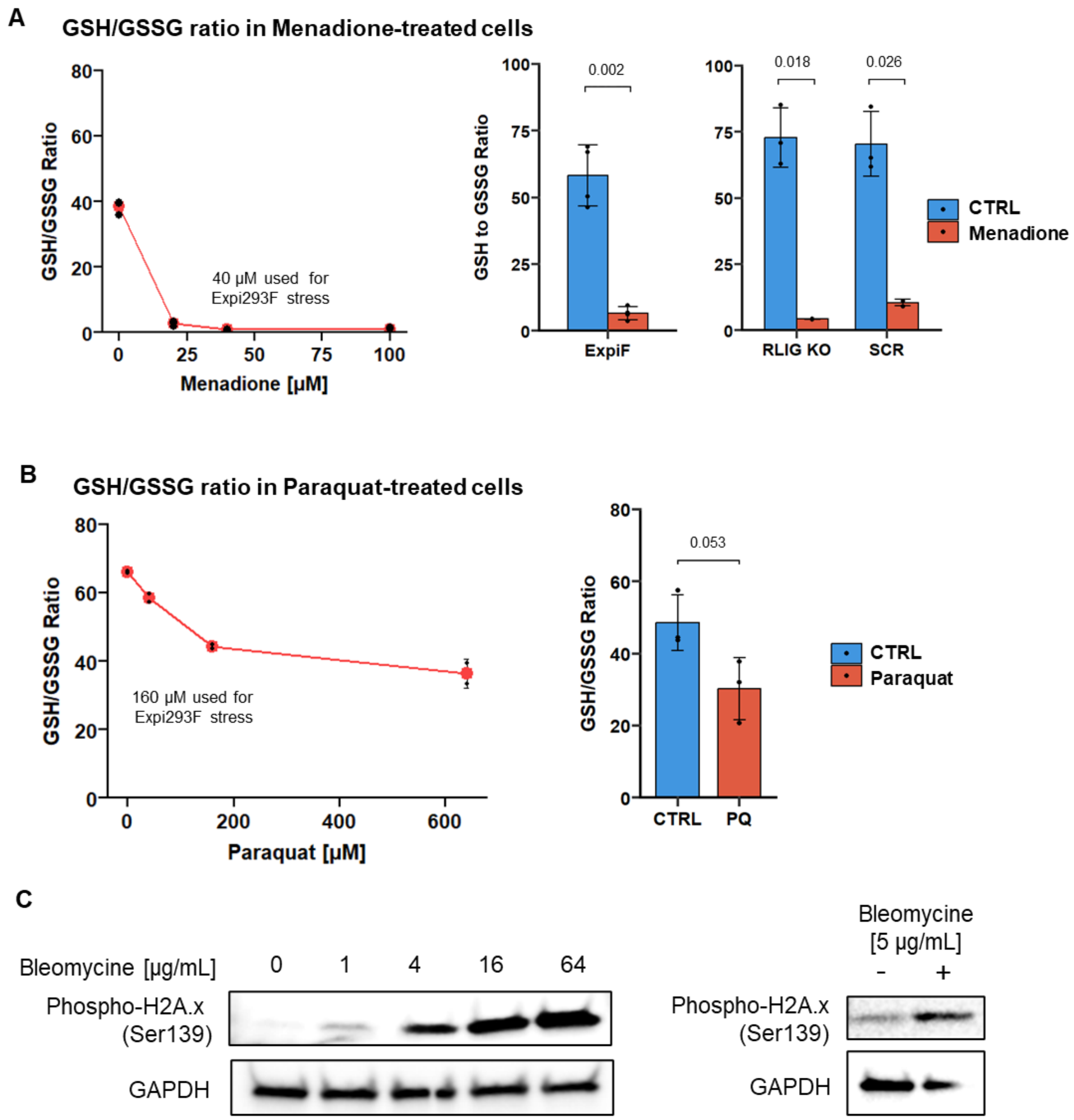

Figure S4: The characterization of cellular stress treatments. A) The GSH/GSSG ratio in Menadione-treated cells measured after 3.5 hours of stress. From the initial screen of Menadione concentration (left panel), 40 μM menadione concentration was selected for all subsequent experiments. Representative GSH/GSSG ratio measurement from triplicate biological experiments in Expi293F, RLIG1 KO and Scrambled (SCR) cell lines used for LC-MS analysis. **B)** The GSH/GSSG ratio in Paraquat-treated cells after 3.5 hours of stress. From the initial screen of suitable Paraquat concentration (left panel), 160 μM Paraquat was selected for all subsequent experiments. The representative GSH/GSSG measurement of a biological triplicate experiment used for LC-MS analysis. **C)** The DNA-damage caused by Bleomycine stress was assessed by western blot using the Phospho-H2Ax antibody. Based on the initial screen for suitable Bleomycine concentration (left panel), 5 μg/mL was selected for all subsequent experiments. The representative western blot of cells used for LC-MS analysis The values above the column graphs in A and B represent the p-value of a pairwise t-test.

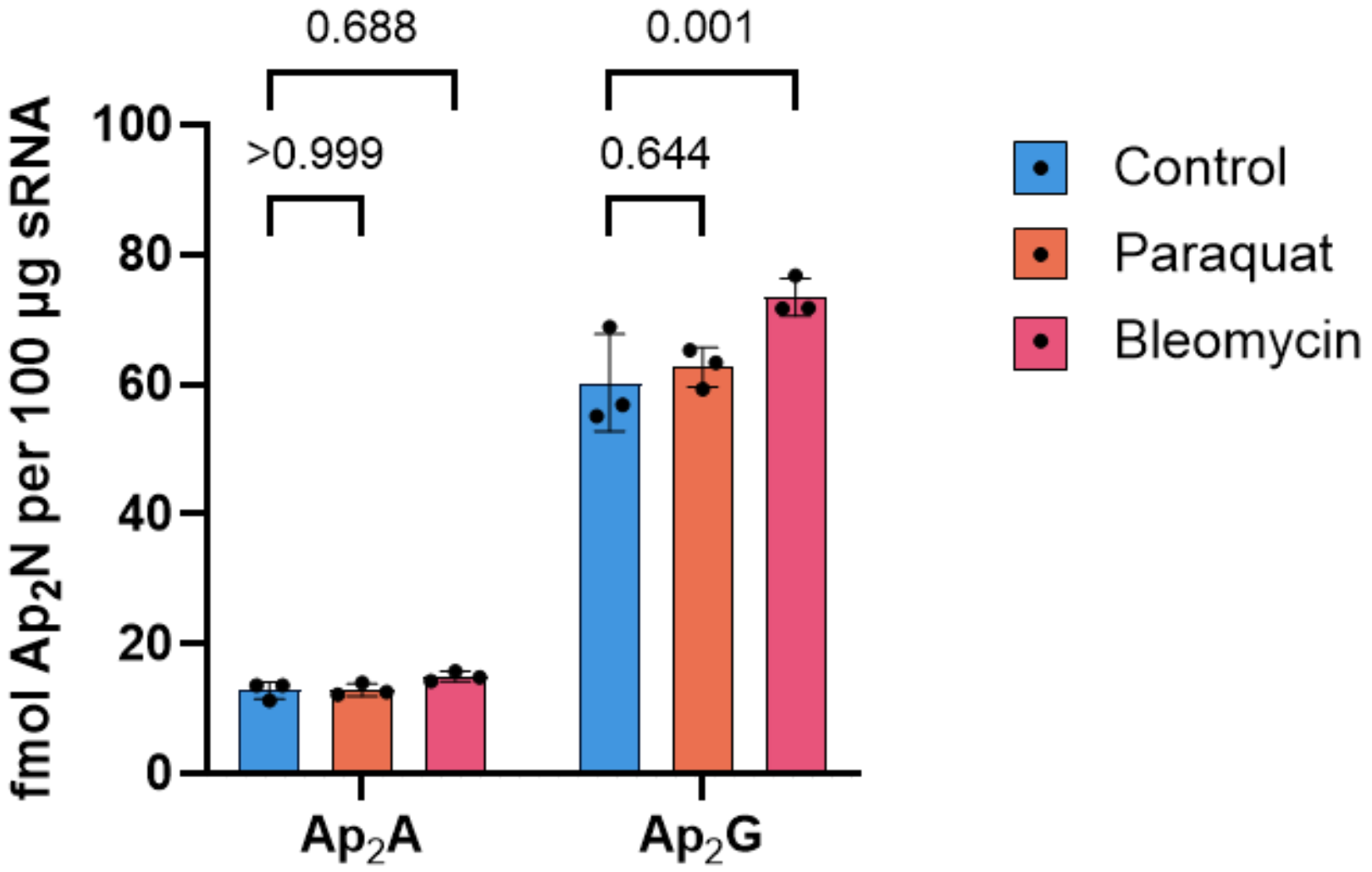

Figure S5: Comparison of changes in Ap_2A- and Ap_2G-RNA amount under Paraquat and Bleomycin stress. LC-MS quantification of Ap_2A-RNA and Ap_2G-RNA caps in sRNA, isolated from control, Paraquat and Bleomycin-treated Expi293F cells. Data are shown as mean ± standard deviation from three biological replicates. Level of significance was calculated using paired-t-test and expressed as p-value.

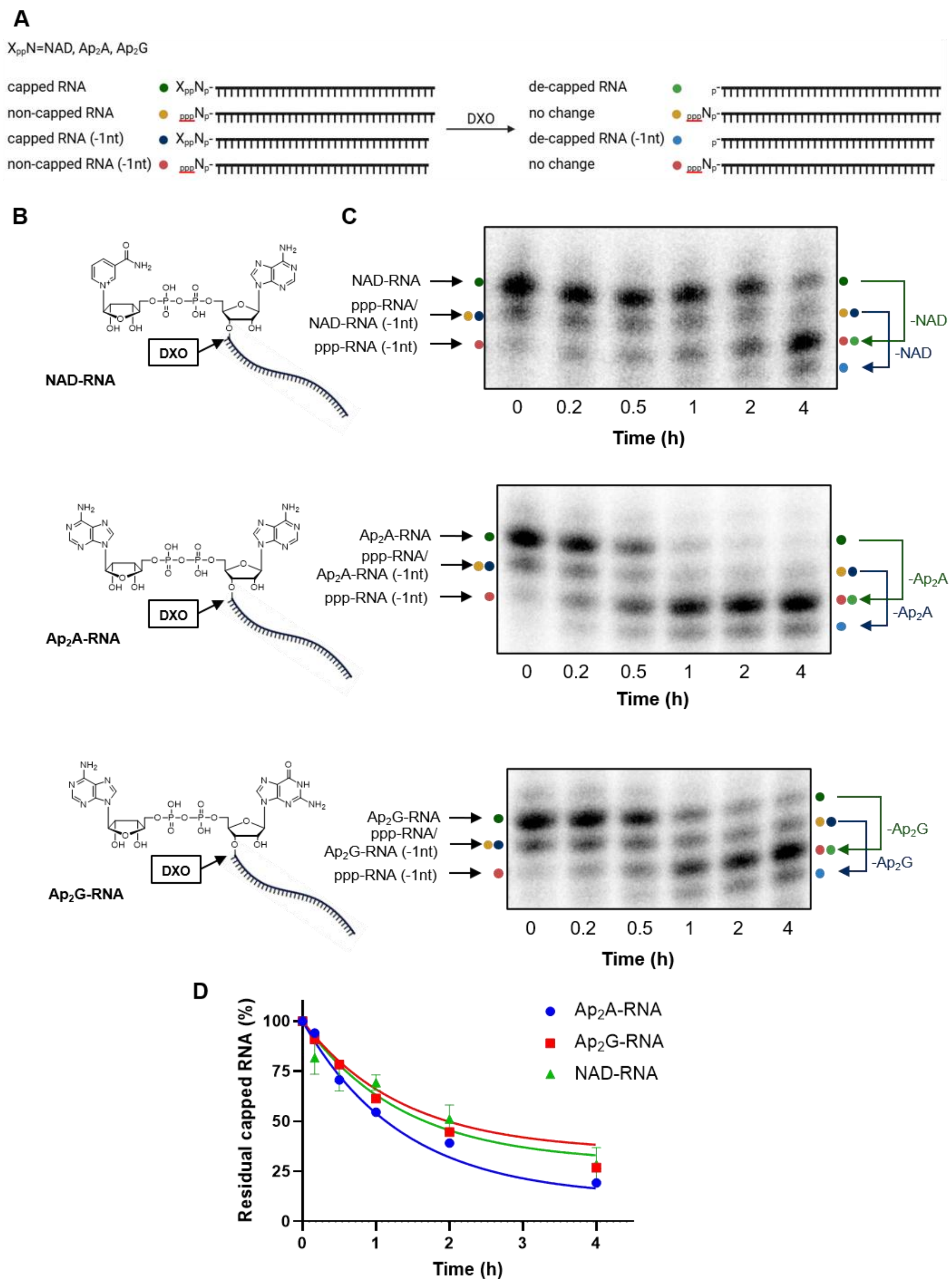

Figure S6: Decapping of NAD-, Ap$_2$A- and Ap$_2$G-RNA by DXO. A) Scheme of various RNA entities (as products of T7 RNA polymerase) present in the reaction mixture and their changes after DXO treatment. Their color labels match with the PAGE labels in panel C in order to clarify bands interpretation. **B)** Chemical structures of NAD-, Ap$_2$A- and Ap$_2$G-RNA. Position of DXO cleavage at 3'-O of the first nucleotide is indicated by arrows. **C)** Examples of PAGE analysis of ^{32}P-radiolabelled NAD-, Ap$_2$A- and Ap$_2$G-RNA after incubation with DXO (100 nM) in time dependent manner (0-4 h of incubation). **D)** Quantification of RNA decapping activity of DXO and NAD-, Ap$_2$A- and Ap$_2$G-RNA as substrates. Data are shown as mean ± standard deviation from experiments performed in triplicates.

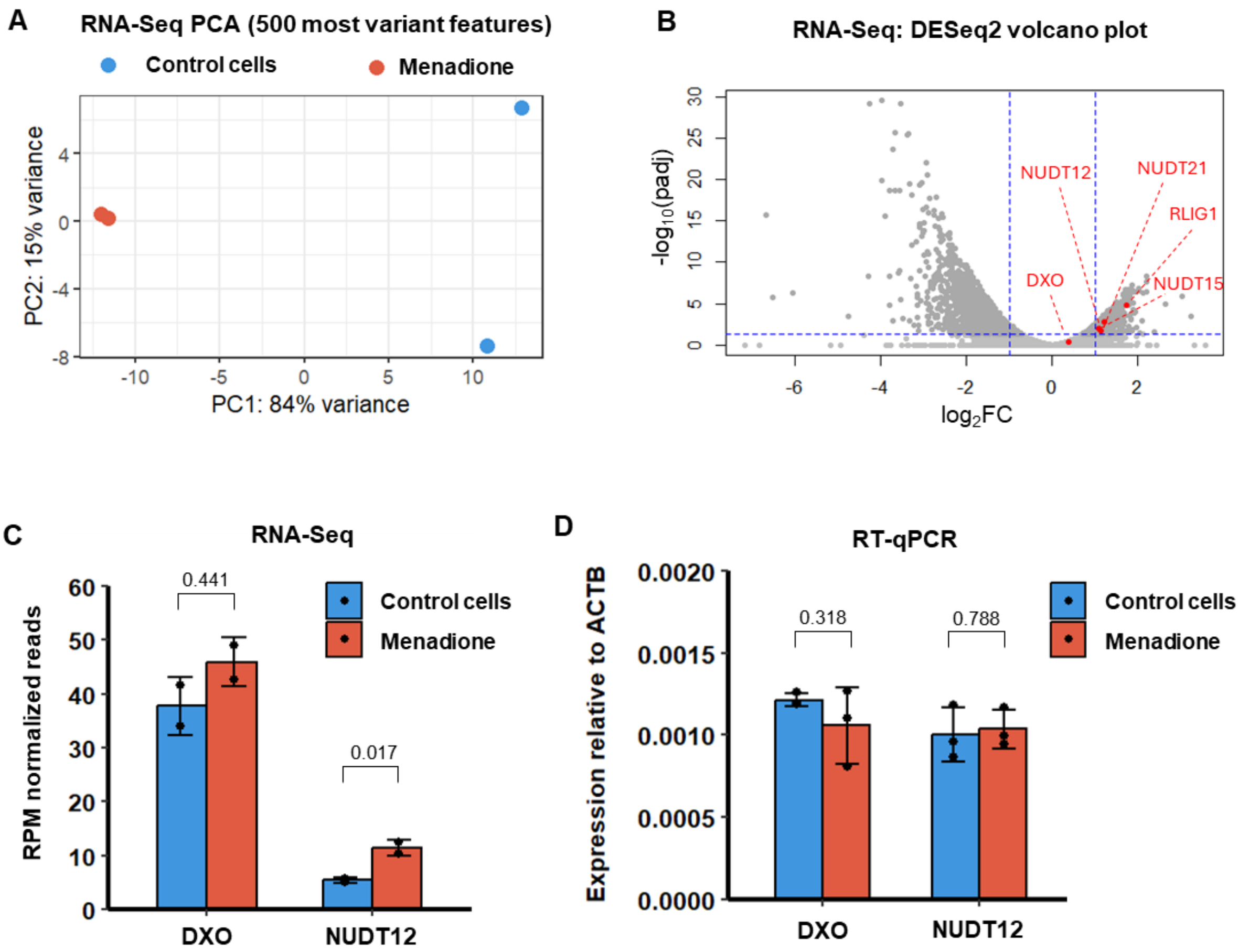

Figure S7: Gene expression analysis control and Menadione-treated cells.
A) Principal component analysis of the duplicate samples. **B)** Volcano plot from DESeq2 analysis with selected genes of interest highlighted in red. See Dataset_S1 for more information. **C)** The gene expression levels based on RNA-Seq of long RNA fraction. The significance bar above the graph shows the p-adujsted value from t-test (n=3). The only significantly enriched Nudix genes were NUDT12, NUDT15 and NUDT21. **D)** The relative expression of selected genes compared to human actin beta measured using RT-qPCR. The error bars represent the standard deviation of the mean and the black dots the individual values.

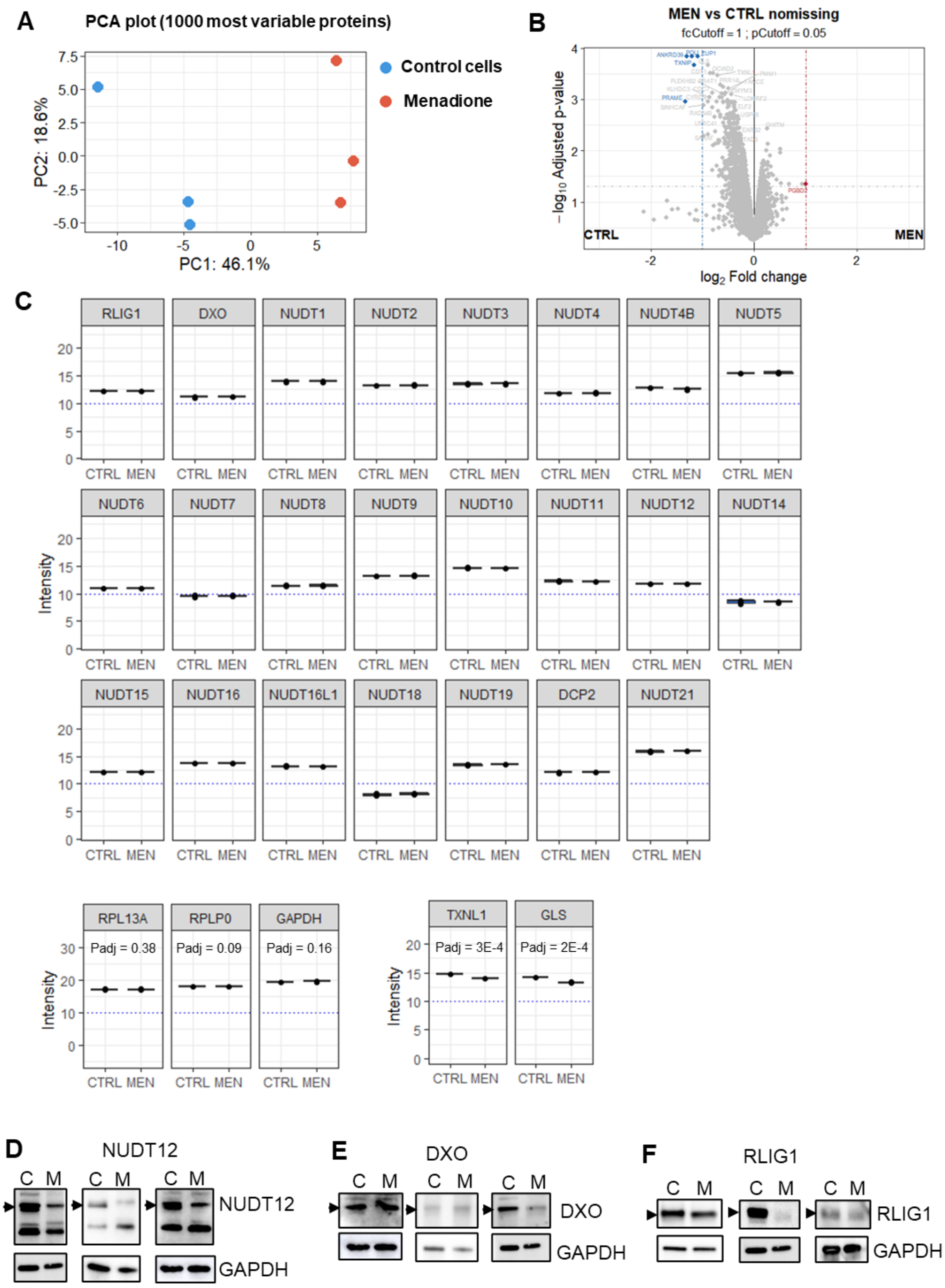

Figure S8: The proteomics analysis of DXO, NUDT enzymes and RLIG1 expression in control cells and Menadione-treated cells. A) The PCA analysis of the biological triplicate samples. Only proteins quantified in all samples were considered. **B)** The volcano plot of identified proteins. Proteins with a q-value < 0.05 and fold change <-2, >2 were considered significantly differentially expressed. **C)** Intensity value comparison of selected proteins in control and stressed cells. None of these proteins was differentially expressed based on the q-value. As negative controls the ubiquitously abundant ribosomal proteins (RPL13A, RPLP0) and GAPDH were used. As positive control indicating stress TXNL 1(18) and GLS(19, 20) proteins were used as dataset-derived validation targets. The data are provided in Dataset S2. **D)** Western blot analysis of NUDT12 levels in control (C) and menadione treated (M) cells. GAPDH protein was used as a loading control. **E)** Western blot analysis of DXO levels in control (C) and menadione treated (M) cells. GAPDH protein was used as a loading control. **F)** Western blot analysis of RLIG1 levels in control (C) and menadione treated (M) cells. GAPDH protein was used as a loading control. Note that GAPDH control was used as normalizer for multiple proteins and therefore the same GAPDH blots are shown in multiple panels.

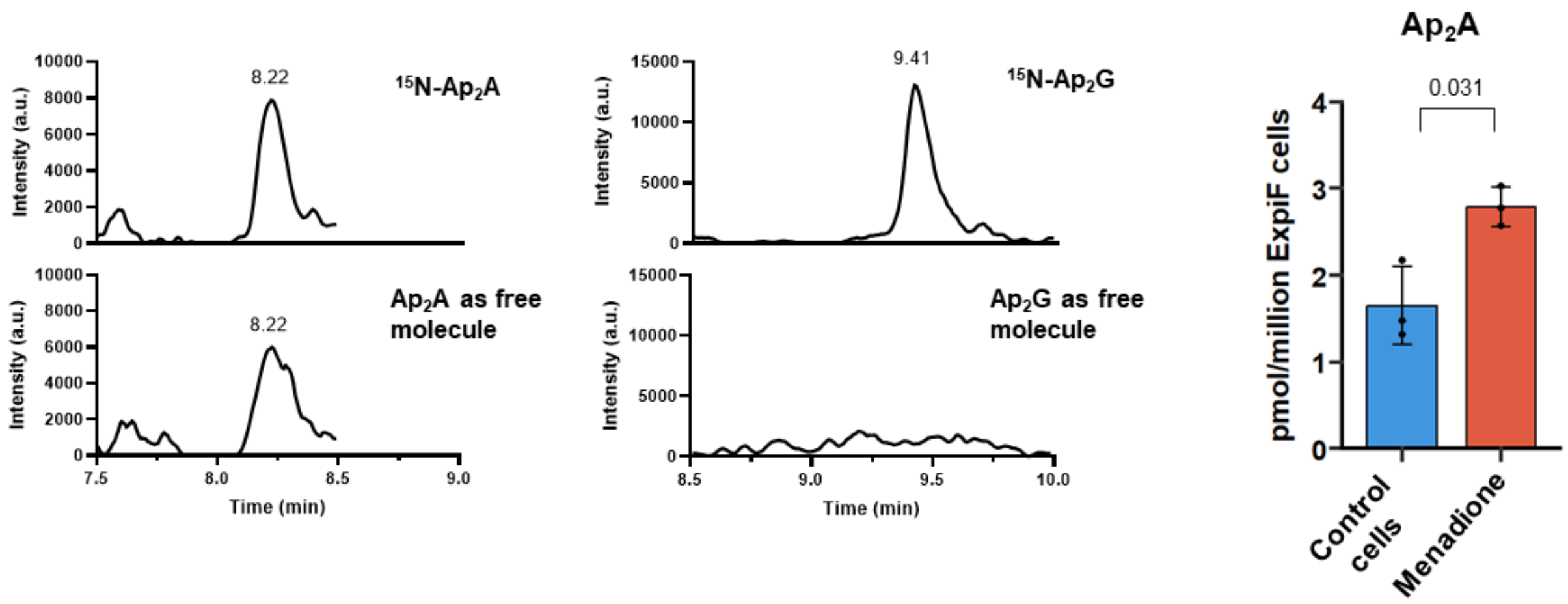

Figure S9: Detection and quantification of free intracellular Ap_2A and Ap_2G.
Presence of Ap_2A and Ap_2G in the fraction of free molecules extracted from Expi293F was analyzed by LC-MS. The chromatograms show MRM transitions 687->255 and 677->250 for ^{15}N-Ap_2A and Ap_2A, respectively (left), and transitions 698->433 and 693->428 for ^{15}N-Ap_2G and Ap_2G, respectively (right). Right-hand panel - quantification of the free Ap_2A metabolite isolated from control (blue) and menadione-treated (red) Expi293F cells. Data are shown as mean ± standard deviation from three biological replicates. Level of significance was calculated using paired-t-test and expressed as p-value.

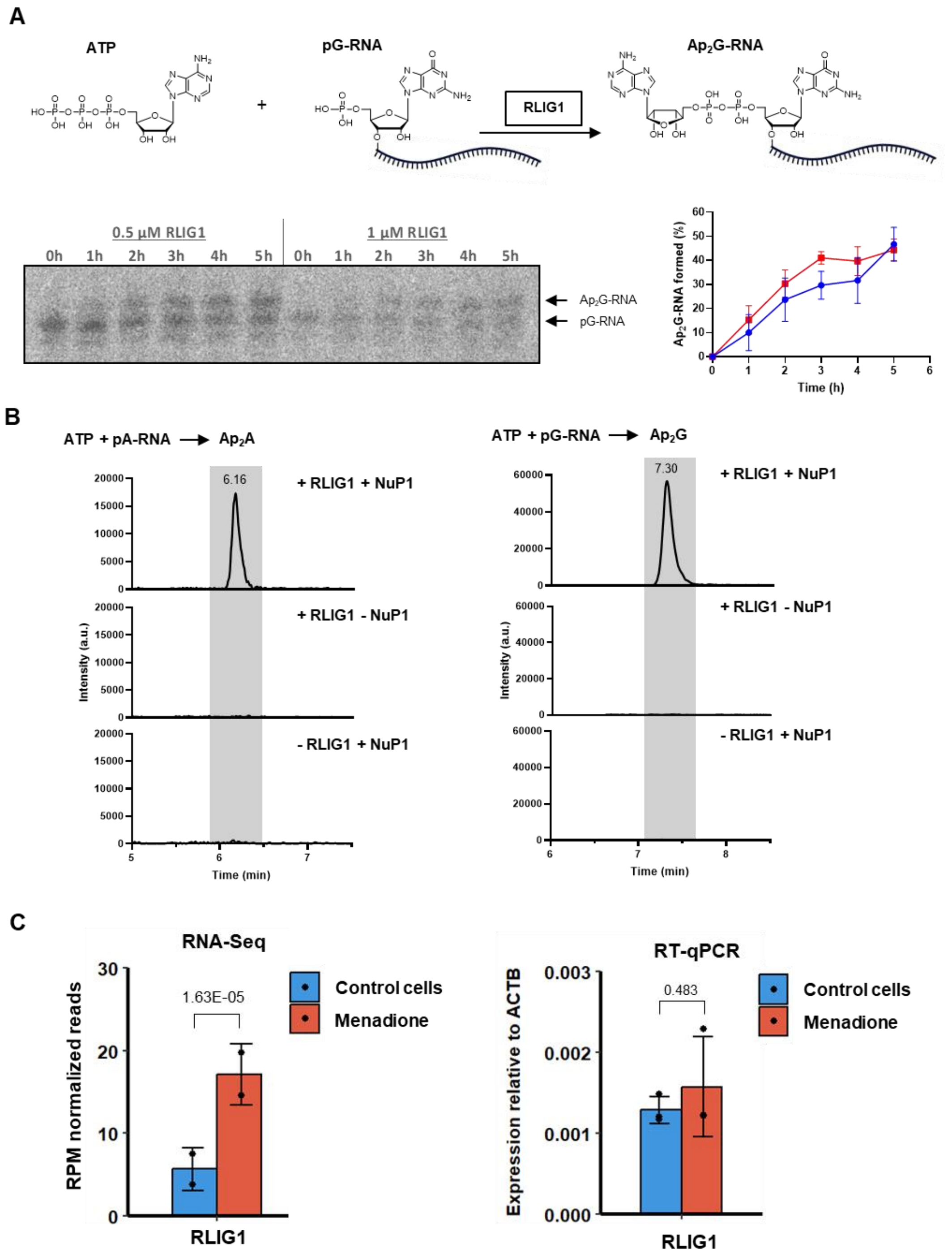

Figure S10: The formation of Ap_2N cap by RLIG1 and the expression of RLIG1 mRNA under menadione stress. A) Ap_2G-RNA formation by RLIG1. Radiolabeled pG-RNA was incubated with 0.5 or 1 µM RLIG1 and ATP for 5h. Ap_2G-RNA formation was monitored and quantified after separation on PAGE. **B)** LC-MS confirmation of Ap_2N formation by RLIG1. pA- and pG-RNA were incubated with or without RLIG1 and Ap_2N-RNA formation was analyzed by LC-MS after digestion with or without NuP1. The chromatograms show MRM transitions 677->250, and 693->428 for Ap_2A and Ap_2G, respectively. **C)** The expression of RLIG1 in control cells and menadione-treated cells in short RNA-seq data. The significance value represents the adjusted p-value of the DESeq2 analysis. Right panel: the expression of RLIG1 detected by RT-qPCR. The significance value represents the p-value of pairwise t-test.

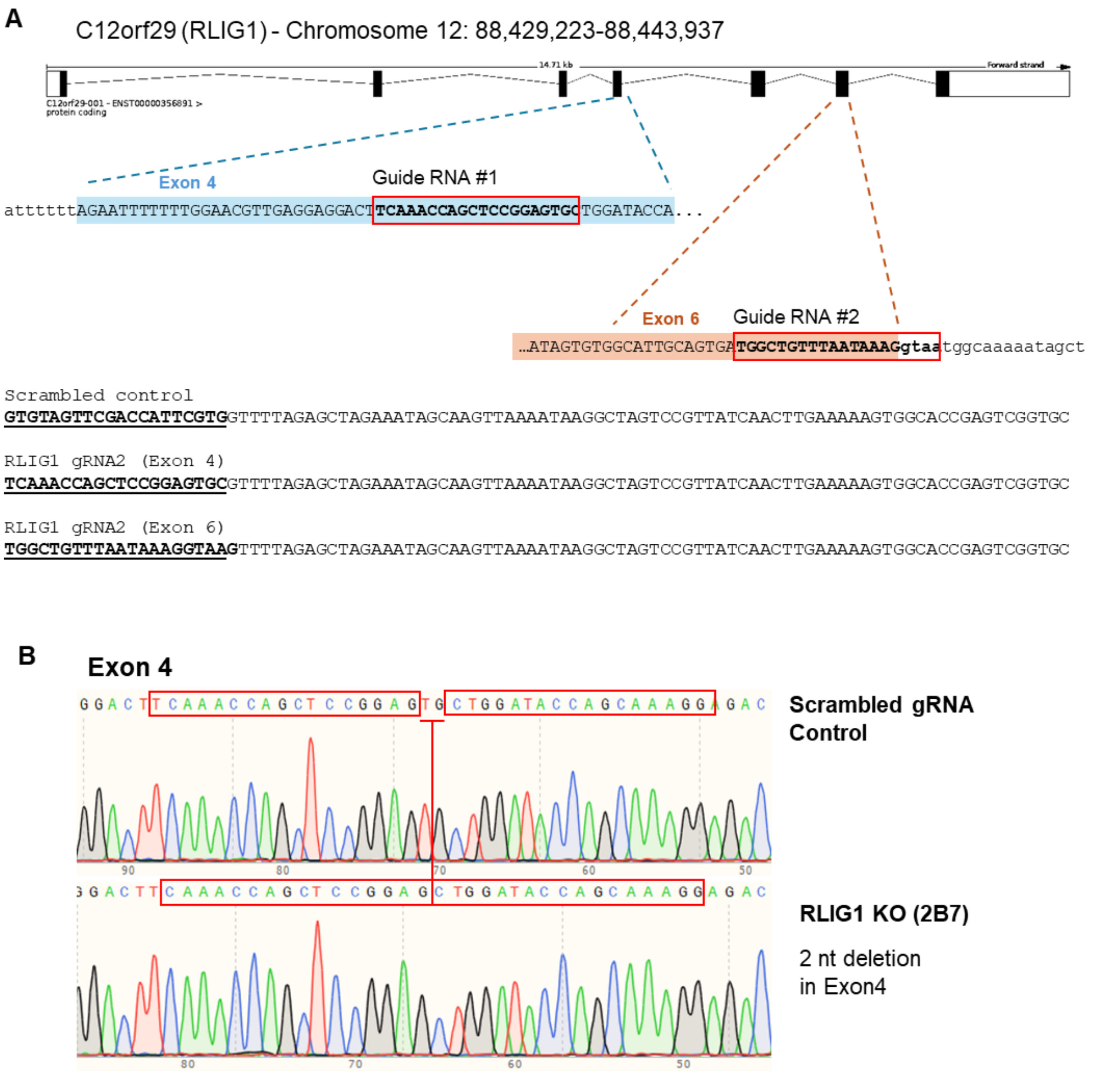

Figure S11: Generation of the RLIG1 knock-out line. A) The design of CRISPR/Cas9 guide RNAs. Two guide RNAs targeting the 4th exon (gRNA #1) and the boundary between Exon4 and adjacent intron (gRNA#2) were used in combination. As a negative control, the scrambled guide RNA was used. **B)** The chromatograms of the Sanger sequencing tracks of the genomic PCR product around the target sequence. The RLIG1 knock-out clone used in this study has 2 nt (TG) deletion within the 4th exon leading to frame shift within the coding sequence.

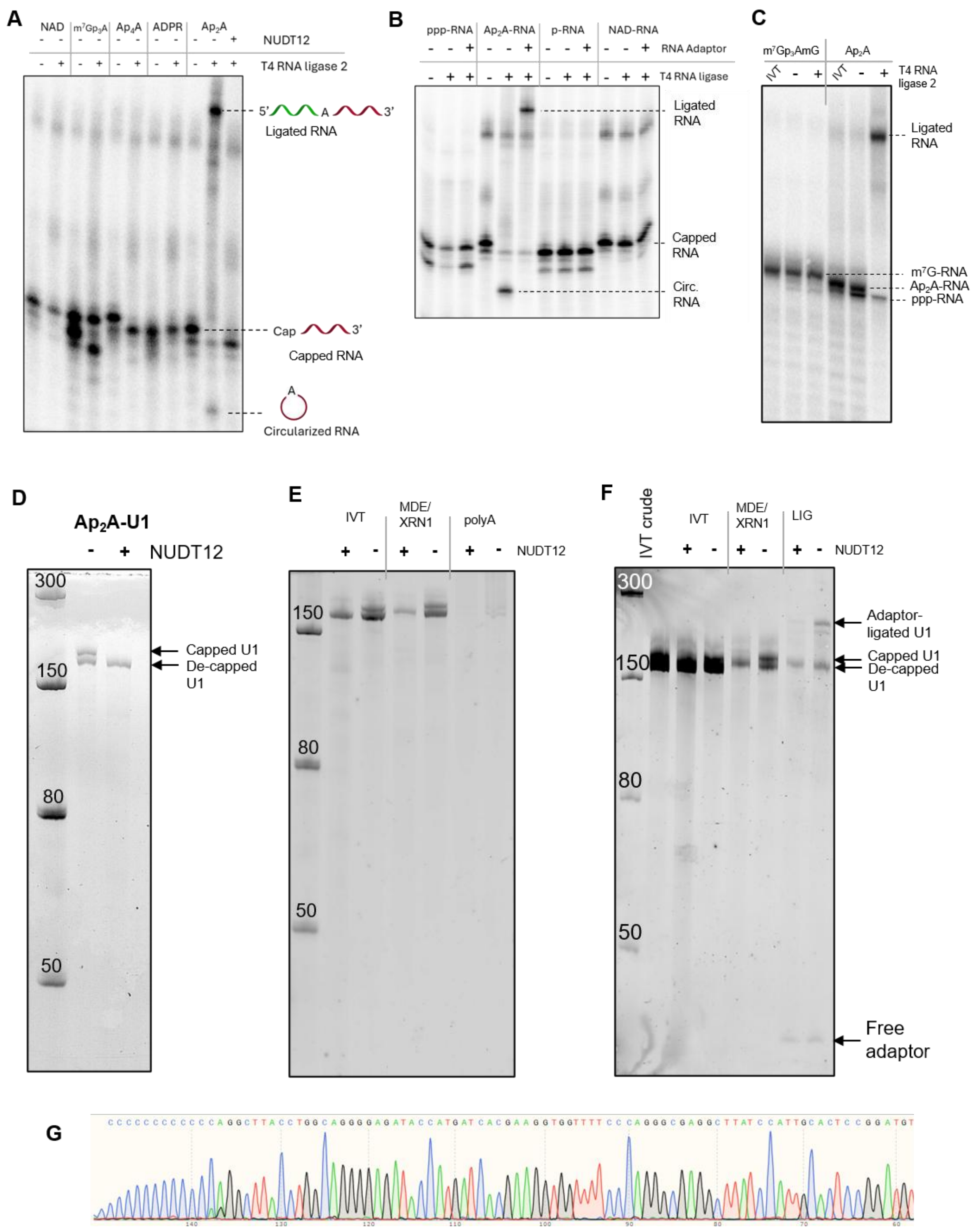

Figure S12: Development of the Ap_2N-RNA seq method. A, B, C) Ligating activity of truncated T4 RNA ligase2 (K227Q) on various RNA caps. The specificity of the truncated T4 RNA ligase 2 towards Ap_2N-capped RNA is demonstrated by the ligation of the RNA adaptor only with Ap_2A-RNA not treated by NUDT12, resulting in a shift of radioactive labelled 35-mer RNA to higher molecular weight. Note that without the presence of the adapter, the 35-mer RNA is self-ligated and forms a fast-migrating product (unexpectedly, in comparison with longer RNA, this circular 35-mer RNA migrates on PAGE gel faster than its linear form). The extra bands arise from known T7 IVT byproducts and do not alter interpretation. **C)** Testing of truncated T4 RNA ligase 2 activity on IVT radioactive labeled 35 mer RNA capped either with m^7Gp_3AmG cap (Trilink) or with Ap_2A. Lanes "-" indicate the reaction mixture without added T4 RNA ligase truncated K227Q. **D)** *In vitro* synthetized Ap_2A-U1 RNA was digested with

NUDT12 (right lane) or incubated in the buffer without the presence of the enzyme (left lane). The products were separated on boronate-containing polyacrylamide gel for better separation of capped and non-capped gel. **E)** *In vitro* synthetized Ap$_2$A-U1 RNA was digested with NUDT12 (+) or incubated in the buffer without the presence of the enzyme (-). Afterwards, the RNA was treated with MDE and XrnI (as in the sequencing protocol) and in the last step, the RNA was polyadenylated by *E. coli* polyA polymerase (as in the sequencing protocol). **F)** *In vitro* synthetized Ap$_2$A-U1 RNA was digested with NUDT12 (+) or incubated in the buffer without the presence of the enzyme (-). Afterwards, the RNA was treated with MDE and XrnI (as in the sequencing protocol) and in the last step, the RNA was ligated to adaptor using the truncated T4 ligase 2 K227Q (as in the sequencing protocol). The products were separated on boronate-containing polyacrylamide gel for better separation of capped and non-capped gel. **G)** The Sanger sequencing chromatogram of the U1-RNA ligated to the 5' adaptor within the model in vitro synthetized U1 sequence, the original A(+1)U(+2)A(+3) start is substituted by A(+1)G(+2)G(+3) to allow robust transcription by T7 RNA polymerase.

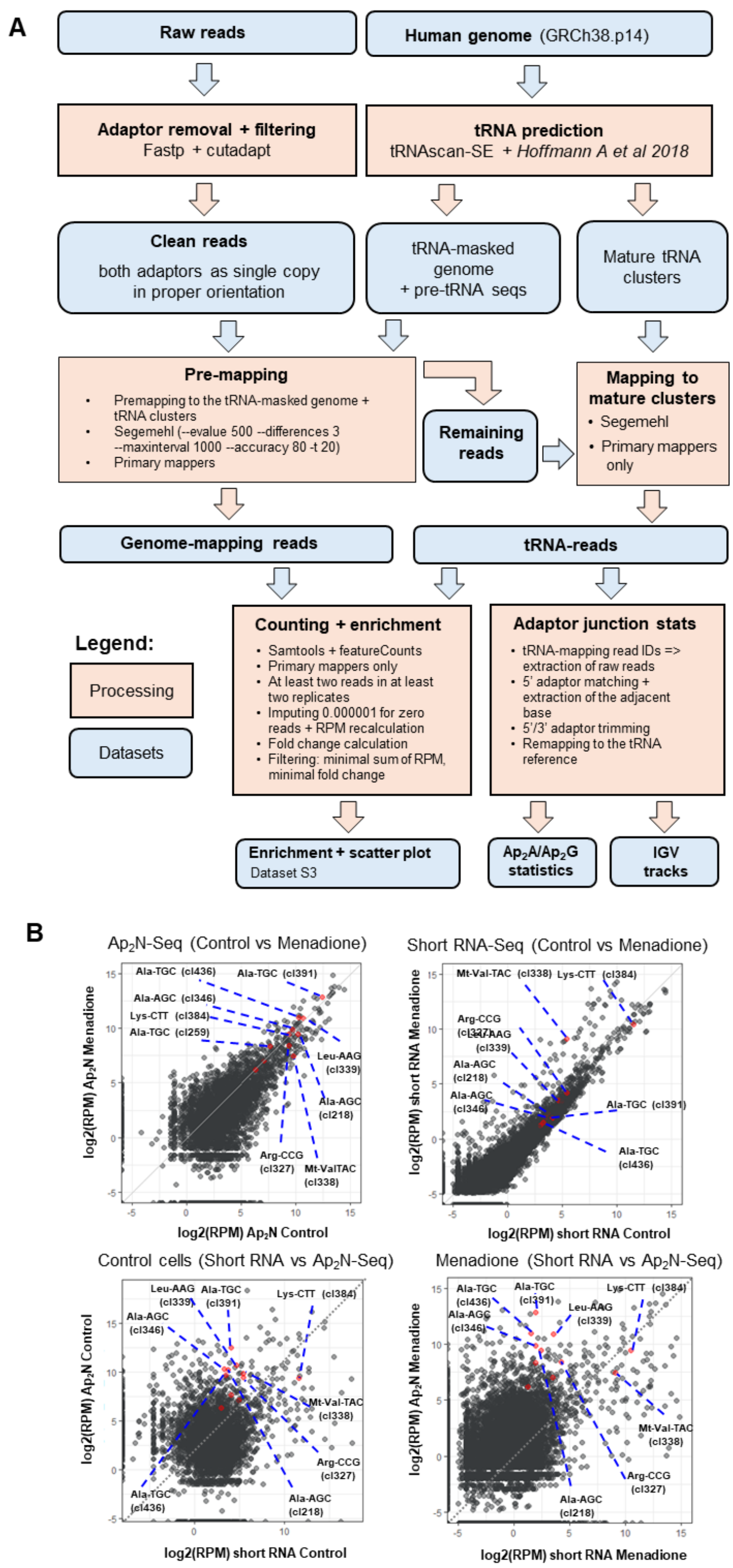

Figure S13: The Ap$_2$N-seq analysis pipeline and the correlations between Ap$_2$N-seq and short RNA-seq experiments. A) The schematic representation of the steps of Ap$_2$N Seq analysis pipeline. The blue rounded rectangles represent the datasets, the orange rectangles represent the processing. **B)** Scatter plots showing the correlations between the average RPM values of each biological replicate at given condition. While the RPM values correlate well between -NUDT12 Ap$_2$N (control cells vs menadione) and Short RNA-Seq (control cells vs menadione) (upper two panels), there is no strong correlation between the Short RNA-Seq and Ap$_2$N RPM values neither in control cells nor in menadione treated cells.

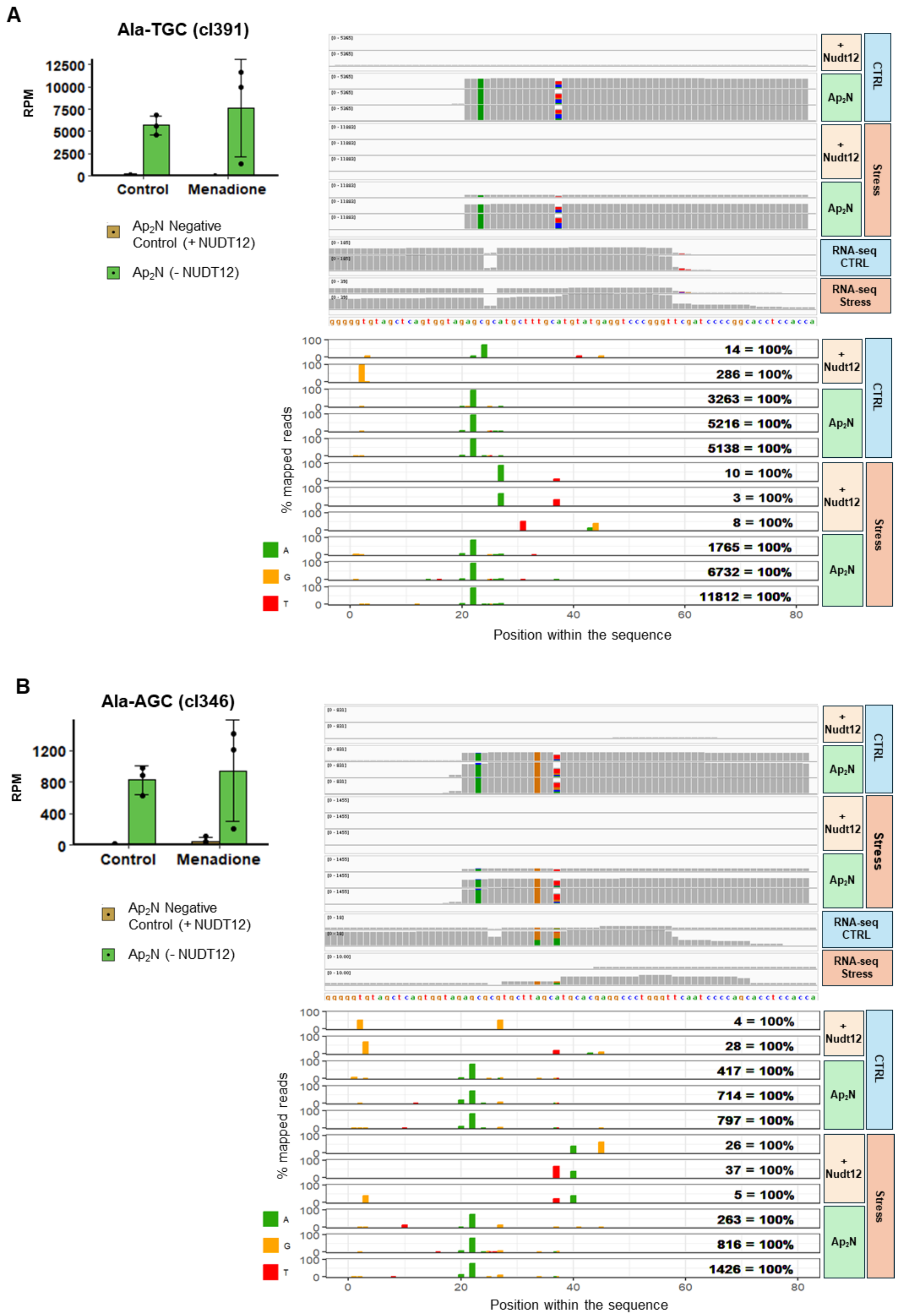
A
Ala-TGC (cl391)
RPM
12500
10000
7500
5000
2500
0
Control
Menadione
Ap2N Negative Control (+NUDT12)
Ap2N (- NUDT12)
+ Nudt12
Ap2N
CTRL
Stress
RNA-seq CTRL
RNA-seq Stress
% mapped reads
A
G
T
14 = 100%
286 = 100%
3263 = 100%
5216 = 100%
5138 = 100%
10 = 100%
3 = 100%
8 = 100%
1765 = 100%
6732 = 100%
11812 = 100%
Position within the sequence
B
Ala-AGC (cl346)
RPM
1200
800
400
0
Control
Menadione
Ap2N Negative Control (+NUDT12)
Ap2N (- NUDT12)
4 = 100%
28 = 100%
417 = 100%
714 = 100%
797 = 100%
26 = 100%
37 = 100%
5 = 100%
263 = 100%
816 = 100%
1426 = 100%
Position within the sequence

C

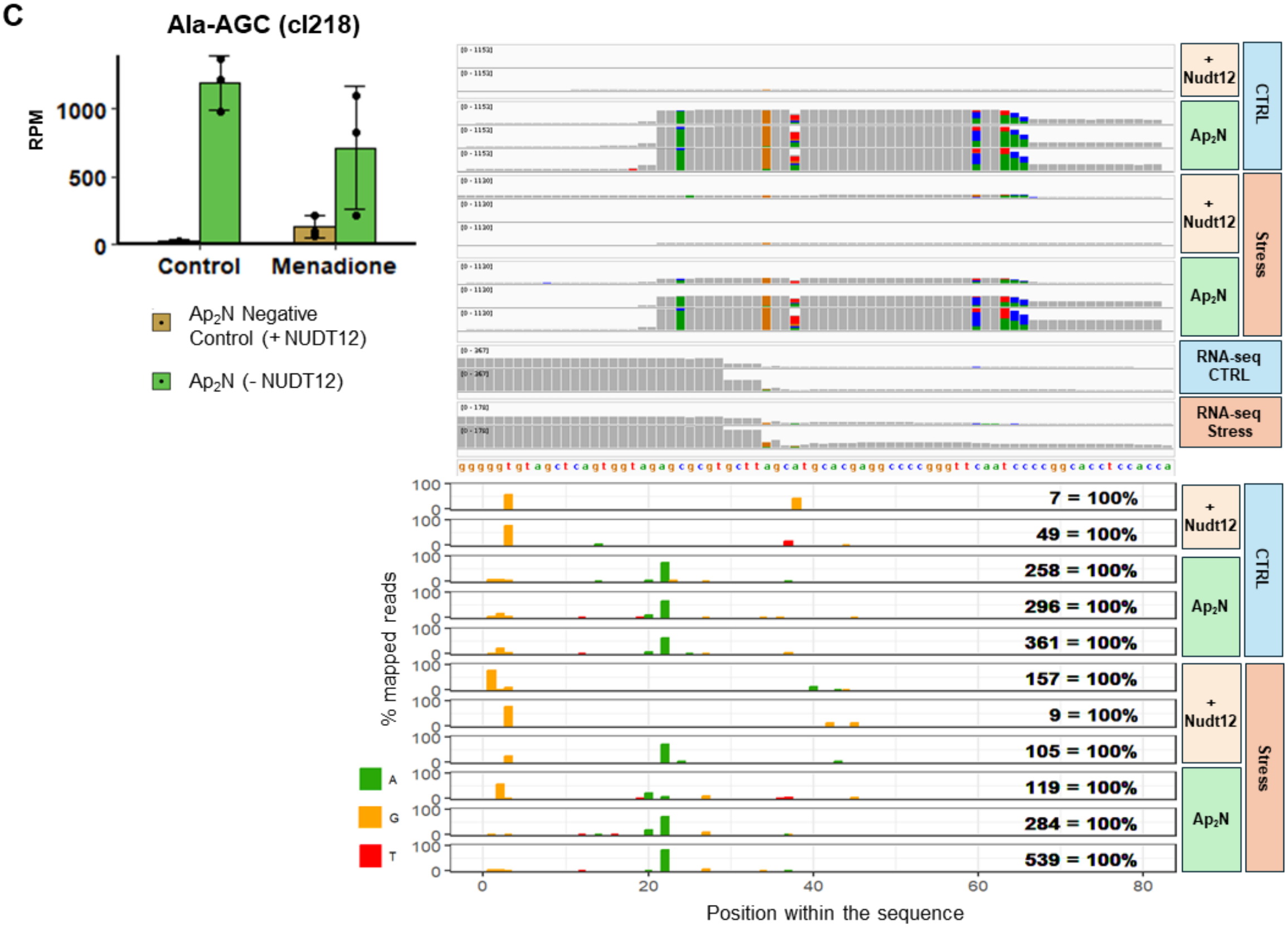
Ala-AGC (cl218)
RPM
1000
500
0
Control
Menadione
Ap2N Negative Control (+ NUDT12)
Ap2N (- NUDT12)
+ Nudt12
Ap2N
CTRL
Stress
RNA-seq CTRL
RNA-seq Stress
% mapped reads
7 = 100%
49 = 100%
258 = 100%
296 = 100%
361 = 100%
157 = 100%
9 = 100%
105 = 100%
119 = 100%
284 = 100%
539 = 100%
A
G
T
Position within the sequence

D

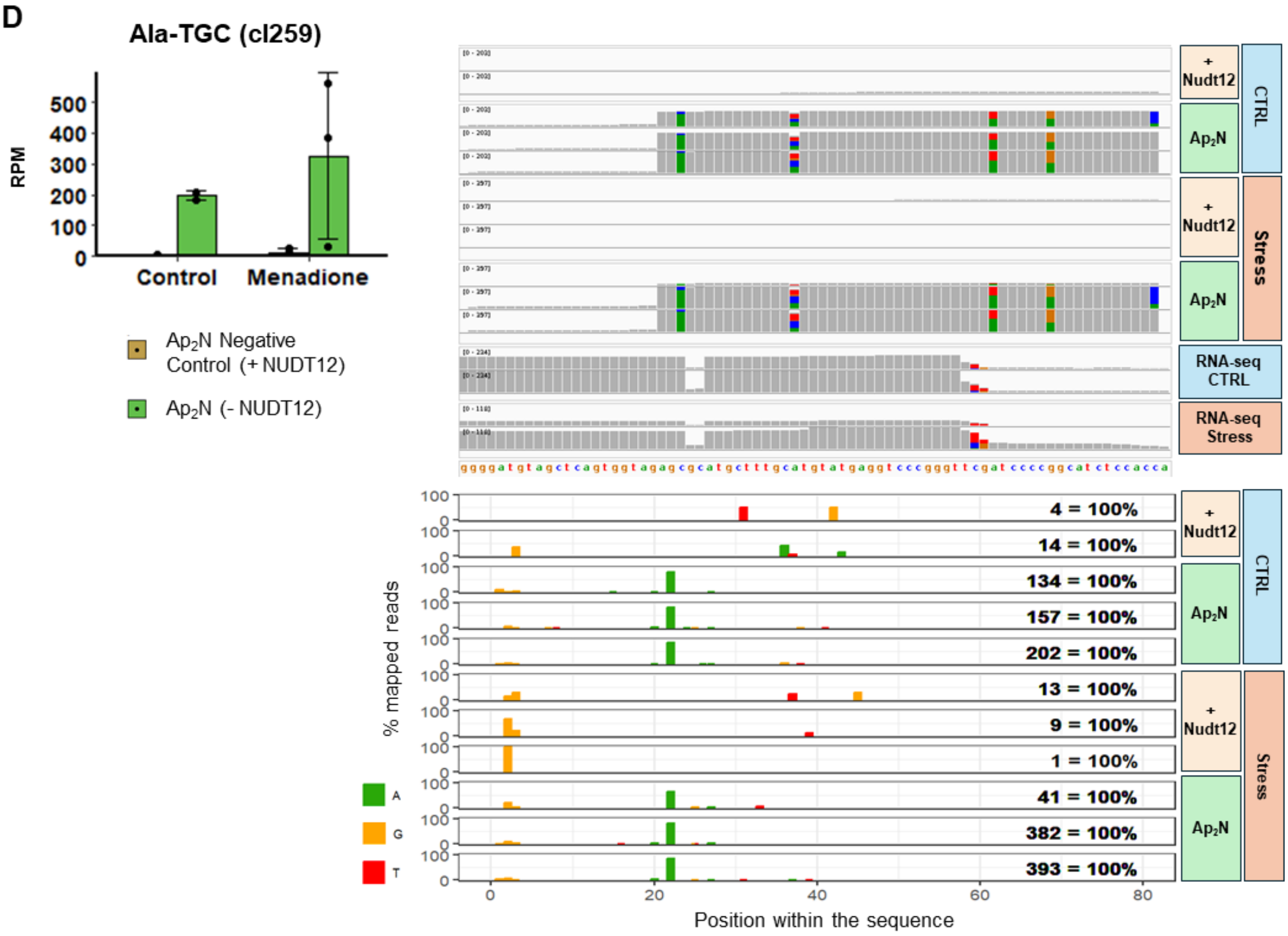
Ala-TGC (cl259)
RPM
500
400
300
200
100
0
Control
Menadione
Ap2N Negative Control (+ NUDT12)
Ap2N (- NUDT12)
+ Nudt12
Ap2N
CTRL
Stress
RNA-seq CTRL
RNA-seq Stress
% mapped reads
4 = 100%
14 = 100%
134 = 100%
157 = 100%
202 = 100%
13 = 100%
9 = 100%
1 = 100%
41 = 100%
382 = 100%
393 = 100%
A
G
T
Position within the sequence

E

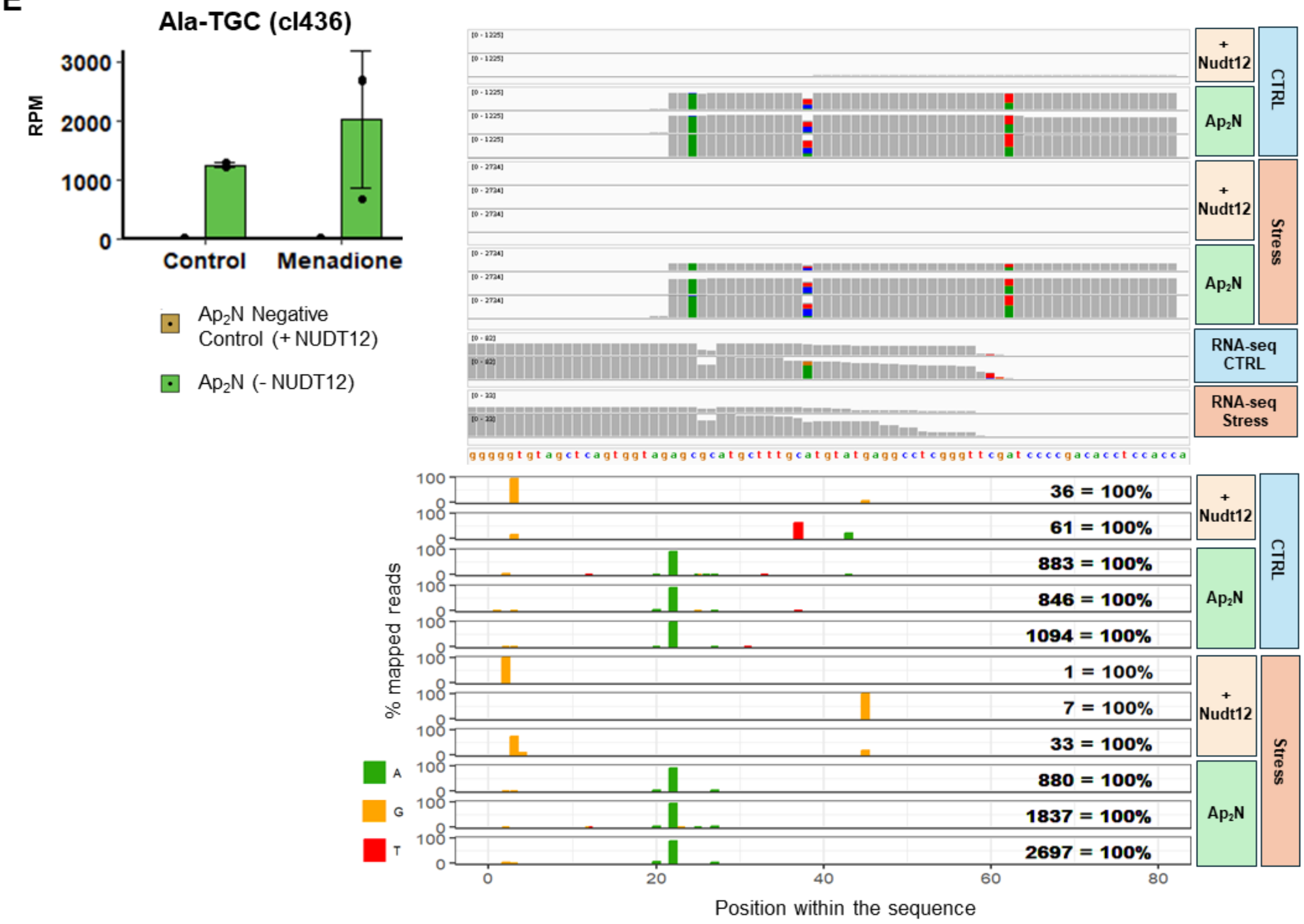
Ala-TGC (cl436)
RPM
3000
2000
1000
0
Control
Menadione
Ap$_2$N Negative Control (+ NUDT12)
Ap$_2$N (- NUDT12)
+ Nudt12
Ap$_2$N
CTRL
Stress
RNA-seq CTRL
RNA-seq Stress
% mapped reads
A
G
T
36 = 100%
61 = 100%
883 = 100%
846 = 100%
1094 = 100%
1 = 100%
7 = 100%
33 = 100%
880 = 100%
1837 = 100%
2697 = 100%
Position within the sequence

F

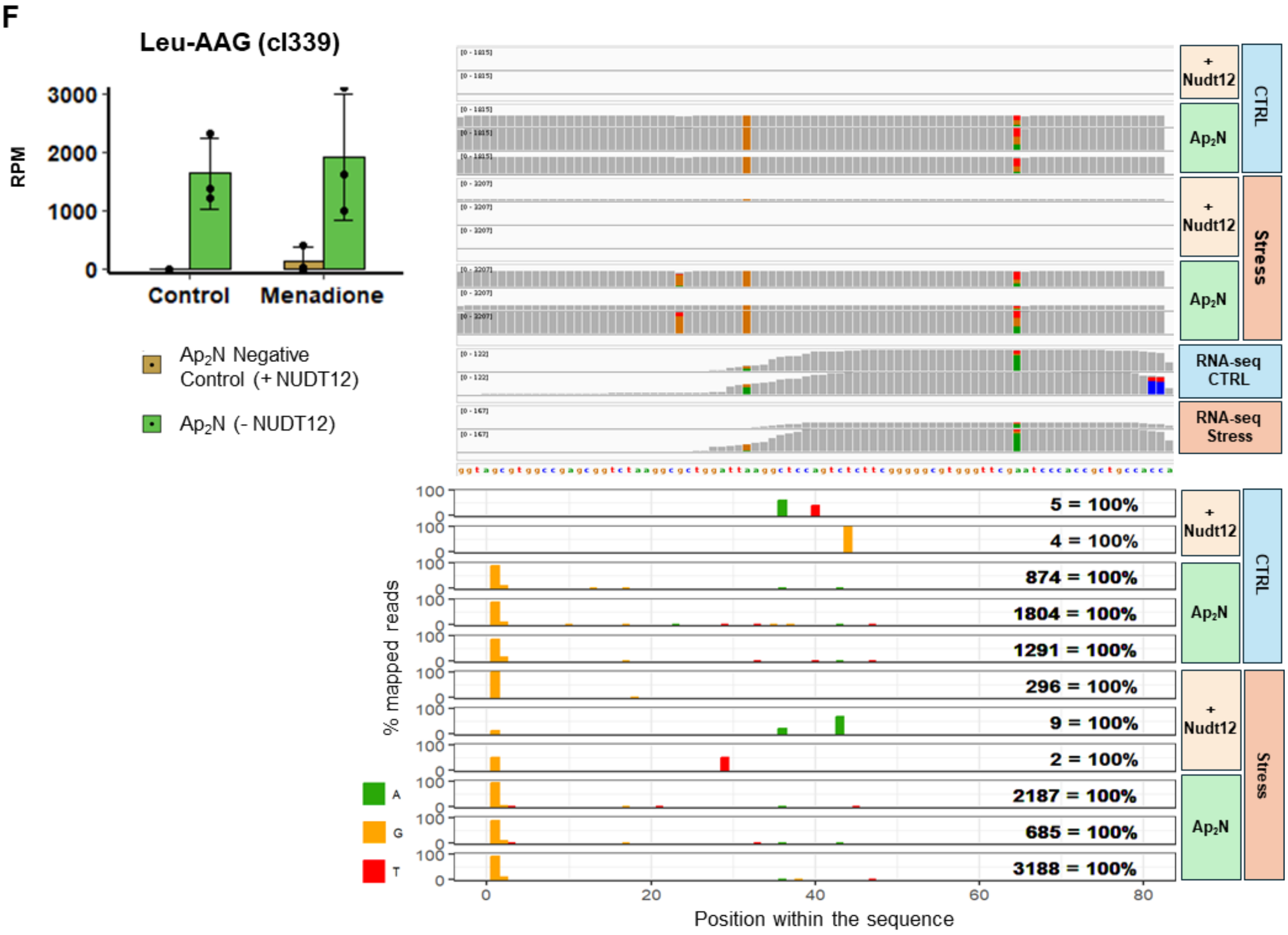
Leu-AAG (cl339)
RPM
3000
2000
1000
0
Control
Menadione
Ap$_2$N Negative Control (+ NUDT12)
Ap$_2$N (- NUDT12)
+ Nudt12
Ap$_2$N
CTRL
Stress
RNA-seq CTRL
RNA-seq Stress
% mapped reads
A
G
T
5 = 100%
4 = 100%
874 = 100%
1804 = 100%
1291 = 100%
296 = 100%
9 = 100%
2 = 100%
2187 = 100%
685 = 100%
3188 = 100%
Position within the sequence

I

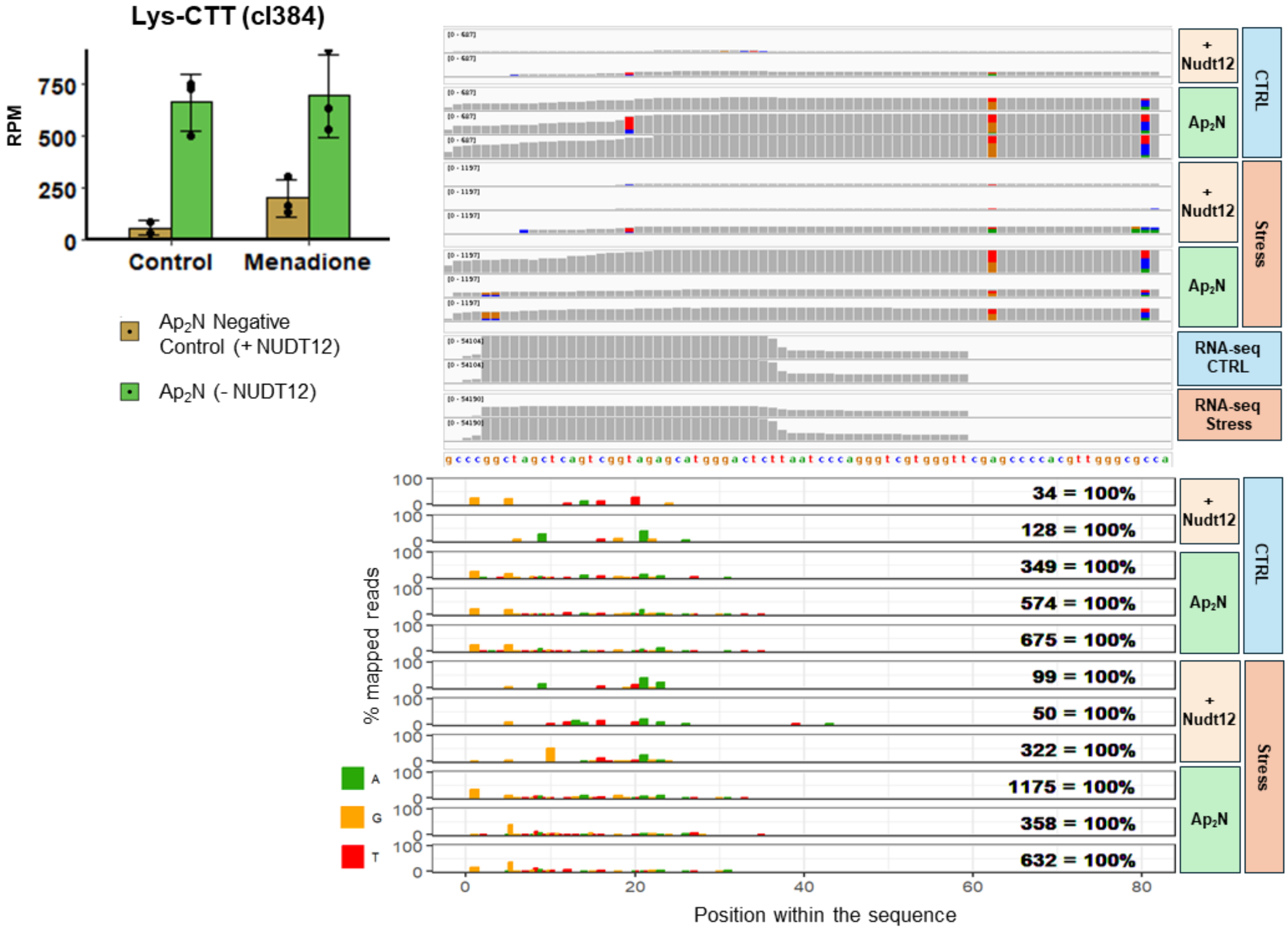
Lys-CTT (cl384)
RPM
750
500
250
0
Control
Menadione
Ap2N Negative Control (+ NUDT12)
Ap2N (- NUDT12)
+ Nudt12
Ap2N
CTRL
Stress
RNA-seq CTRL
RNA-seq Stress
% mapped reads
A
G
T
34 = 100%
128 = 100%
349 = 100%
574 = 100%
675 = 100%
99 = 100%
50 = 100%
322 = 100%
1175 = 100%
358 = 100%
632 = 100%
Position within the sequence

J

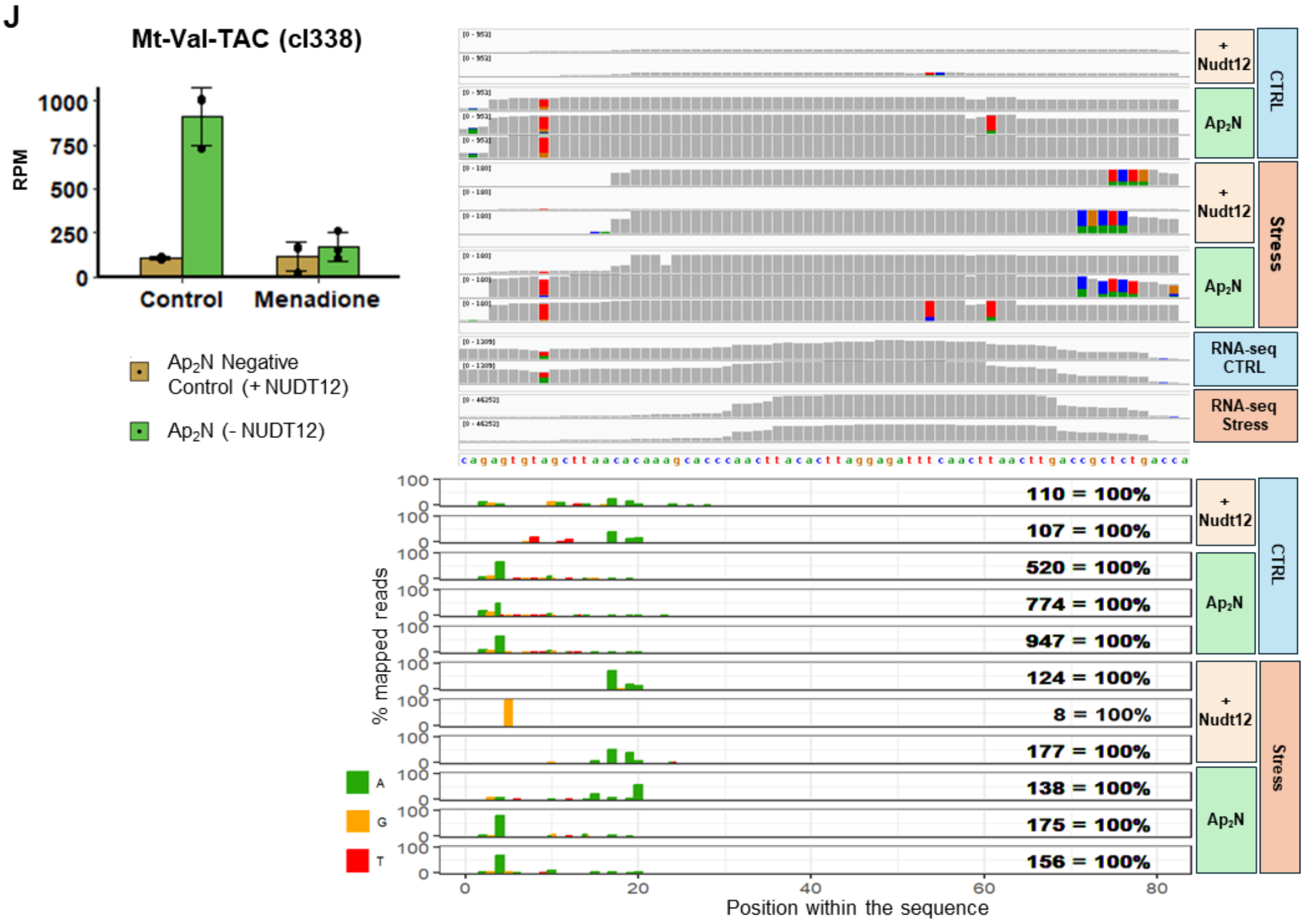
Mt-Val-TAC (cl338)
RPM
1000
750
500
250
0
Control
Menadione
Ap2N Negative Control (+ NUDT12)
Ap2N (- NUDT12)
+ Nudt12
Ap2N
CTRL
Stress
RNA-seq CTRL
RNA-seq Stress
% mapped reads
A
G
T
110 = 100%
107 = 100%
520 = 100%
774 = 100%
947 = 100%
124 = 100%
8 = 100%
177 = 100%
138 = 100%
175 = 100%
156 = 100%
Position within the sequence

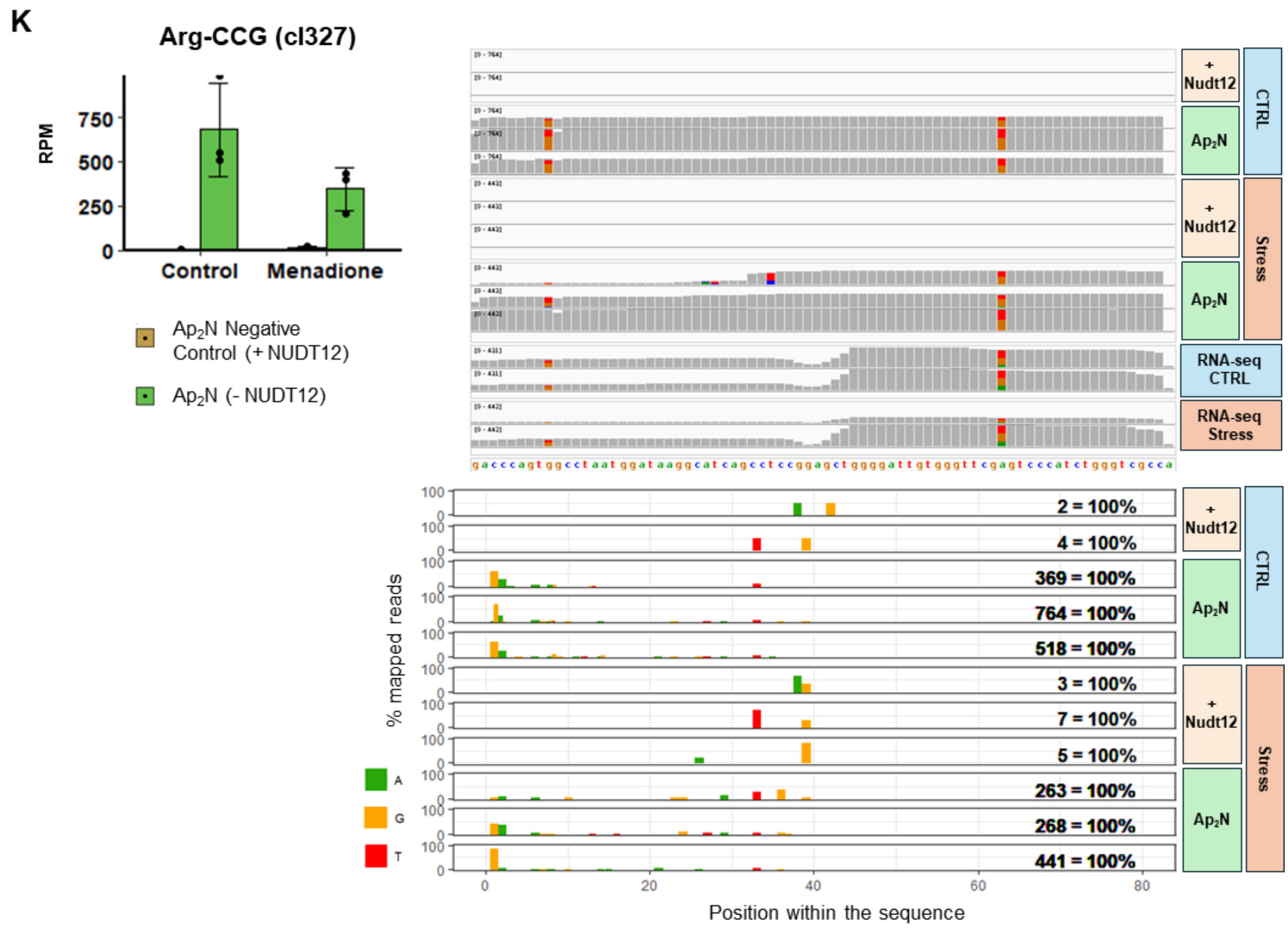

Figure S14: **The detailed results of the Ap$_2$N-RNA Seq for most enriched tRNAs**. The left panel represents the normalized number of reads per million of mapped reads (RPM) in control cells and menadione-treated samples. The green bars present the RPMs for the Ap$_2$N-RNA (without NUDT12 treatment) whereas the orange bars show the RPMs of the hits in negative control samples where Ap$_2$N was removed by incubation with NUDT12. The error bar represents the standard deviation of the mean and the black dots the individual values. The right panel shows the IGV snapshot of the mapped reads in each of the condition indicated by righ-hand sided rectangles (yellow: +NUDT12 represents decapped negative control, green: Ap2N-seq protocol (no Nudt12 treatment), blue: control cells, red: menadione-treated cells). The IGV tracks are group-scaled based on stressed/non-stressed condition. Bottom panel represents the bases along the tRNA sequence that have been detected as the first base downstream of the ligated 5' adaptor (green: A, yellow: G, red: T/U). The experimental conditions are given by the colored rectangles on the right-hand side. The total count of reads with the adaptor is given as 100% in each experimental condition and the tracks are scaled to 0-100%.

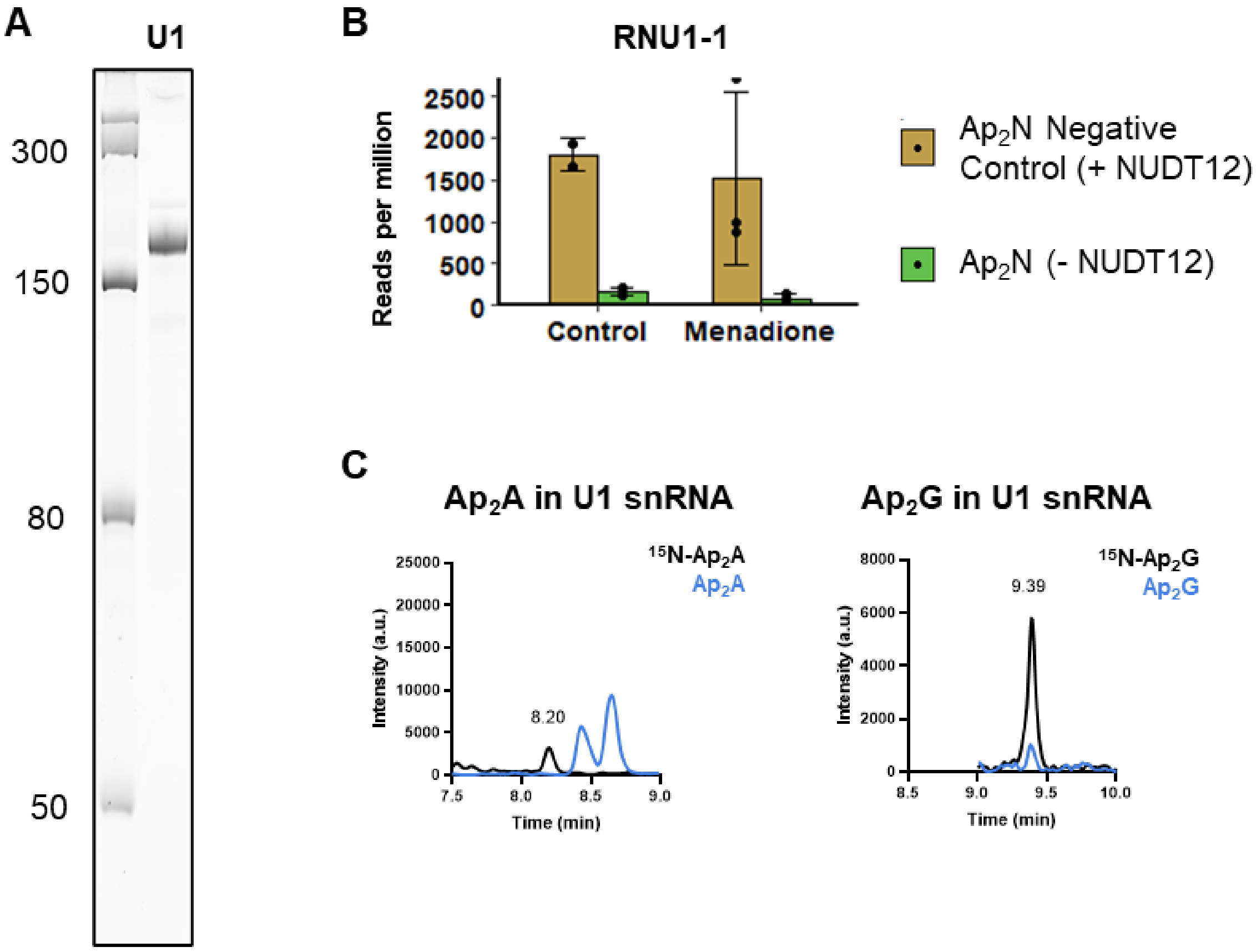

Figure S15: Detection of of Ap_2A- and Ap_2G-RNA in U1 RNA pull-down. A) An 8% PAGE gel of the U1 (RNU-1-1) pull-down RNA (~164 nt). The left lane was loaded with ssRNA ladder and the corresponding nt sizes are indicated. **B)** Normalized number of reads per million of mapped reads (RPM) in control cells and menadione-treated samples. The green bars present the RPMs for the Ap_2N-RNA (without NUDT12 treatment) whereas the orange bars show the RPMs of the hits in negative control samples where Ap_2N was removed by incubation with NUDT12. The error bar represents the standard deviation of the mean and the black dots the individual values. **C)** LC-MS analysis of U1 snRNA pull-down samples. The chromatograms show MRM transitions 687.1->255 and 677.1->250 for ^{15}N-Ap_2A and Ap_2A, respectively (left), and MRM transitions 698->433 and 693->428 for ^{15}N-Ap_2G and Ap_2G, respectively (right).

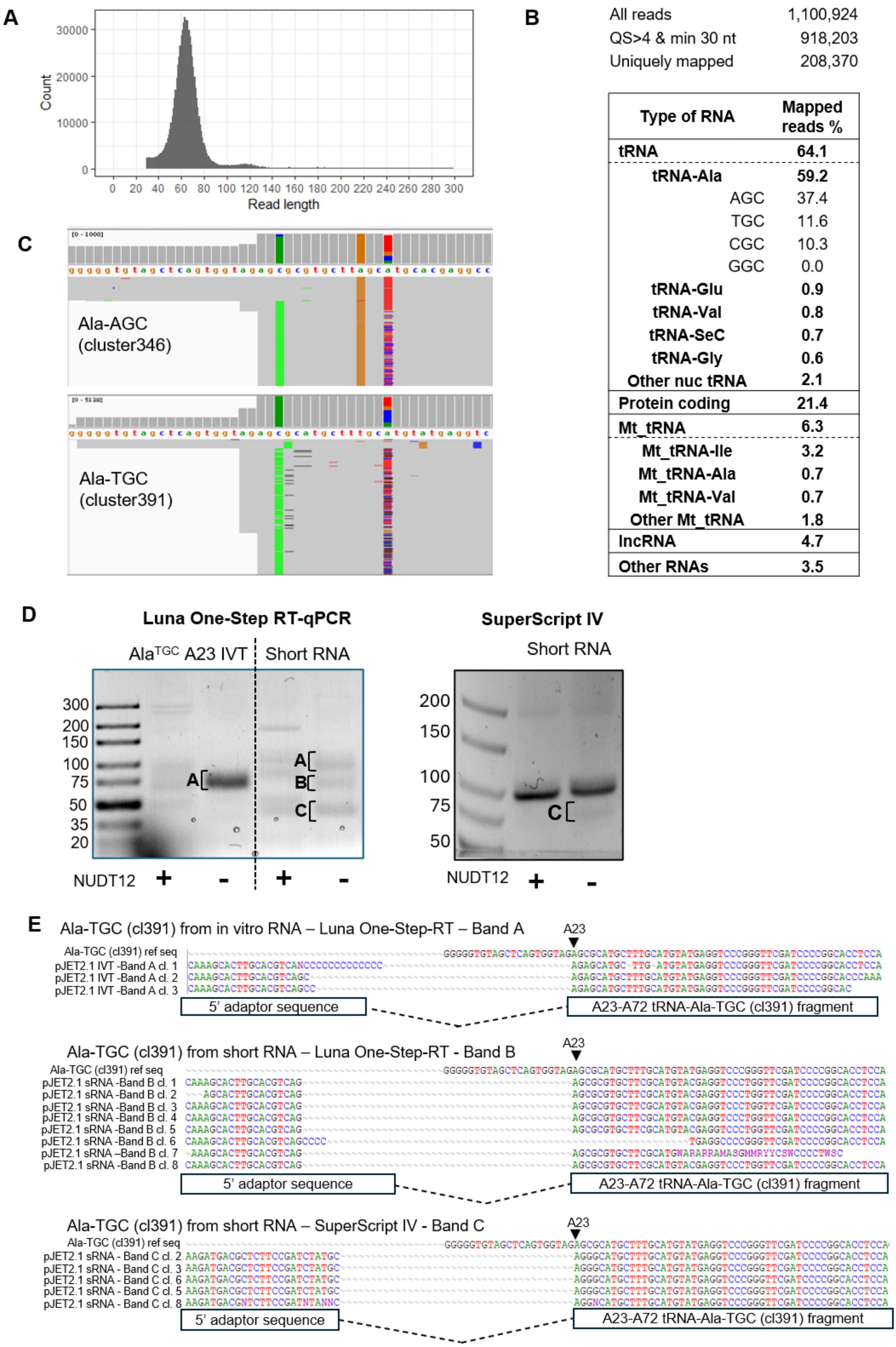

All reads	1,100,924
QS>4 & min 30 nt	918,203
Uniquely mapped	208,370

Type of RNA	Mapped reads %
**tRNA**	**64.1**
**tRNA-Ala**	**59.2**
AGC	37.4
TGC	11.6
CGC	10.3
GGC	0.0
**tRNA-Glu**	**0.9**
**tRNA-Val**	**0.8**
**tRNA-SeC**	**0.7**
**tRNA-Gly**	**0.6**
**Other nuc tRNA**	**2.1**
**Protein coding**	**21.4**
**Mt_tRNA**	**6.3**
**Mt_tRNA-Ile**	**3.2**
**Mt_tRNA-Ala**	**0.7**
**Mt_tRNA-Val**	**0.7**
**Other Mt_tRNA**	**1.8**
**lncRNA**	**4.7**
**Other RNAs**	**3.5**

Figure S16: ONT direct RNA-seq analysis of the deacylated tRNA$^{Ala-AGC}$ pull-down. A) The distribution of the read-lengths in the sequencing data after filtering (QS>4, minimal length 30). **B)** The composition of the pull-down RNA expressed as percentage of the indicated RNA species within the total mapped reads. **C)** Representative views of the IGV tracks of tRNA$^{Ala-AGC}$ and tRNA$^{Ala-TGC}$ from the Ap$_2$N-seq (-NUDT12), showing the minor presence of full-length tRNAs (adaptor attached to the G-base at position 1 or 2). The coverage track is log-scaled for better visibility of the less-abundant full-length reads. Examples of individual reads are shown below the coverage and sequence track, each line representing one read. **D-E)** Confirmation of the tRNA$^{Ala-TGC}$ fragment by orthogonal method: **D)** Agarose gel showing the products of PCR performed with 5' Adaptor Fwd and tRNA$^{Ala-TGC}$ reverse primer on *in vitro* synthetized tRNA$^{Ala-TGC}$ fragment (left side, A23 IVT sample) or short RNA processed by Ap$_2$N-Seq protocol until the adaptor ligation step (included) either with Luna One-Step-RT-qPCR or with SuperScript IV RT. The bands excised for cloning and sequencing are marked by letters A, B and C. **E**) The Sanger-sequenced inserts aligned to the tRNA$^{Ala-TGC}$ reference sequence. The insert sequences are divided into the 5'adaptor part and the immediately adjacent sequence of tRNA$^{Ala-TGC}$ for better clarity. The upper panel shows the result of sequencing the band A in of the in vitro synthetized A23 fragment as positive control (note that for efficient T7 transcription, the initial AGC was changed to AGA in the template).

A

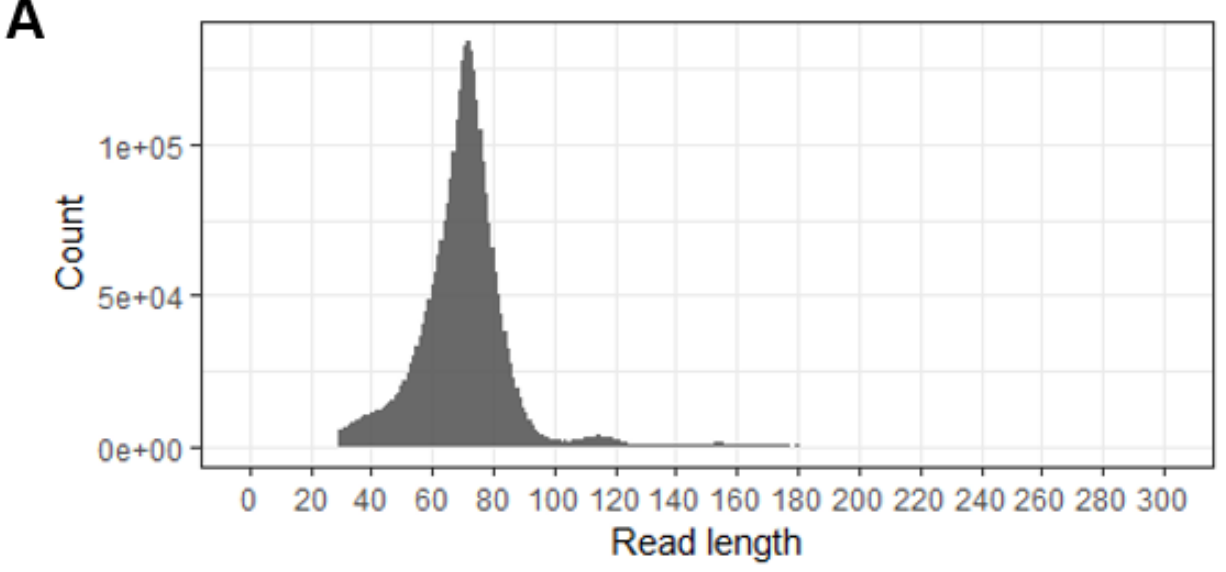

B

All reads	3,434,793
QS>4 & min 30 nt	3,092,335
Uniquely mapped	1,560,682

Type of RNA	Mapped reads %
**Mt_tRNA**	**65.8**
**Mt_tRNA-Val**	**61.0**
**Mt_tRNA-Ser**	**2.1**
**Mt_tRNA-Met**	**1.0**
**Other Mt_tRNA**	**1.7**
**tRNA**	**24.8**
**tRNA-Leu**	20.2
AAG	14.3
TAG	5.7
other tRNA-Leu	0.2
**tRNA-Glu**	**1.1**
**tRNA-Gly**	**1.0**
**Other nuc tRNA**	**2.5**
**Protein coding**	**5.7**
**lncRNA**	**1.5**
**rRNA**	**1.1**
**Other RNAs**	**1.2**

C Leu-AAG(cluster339)

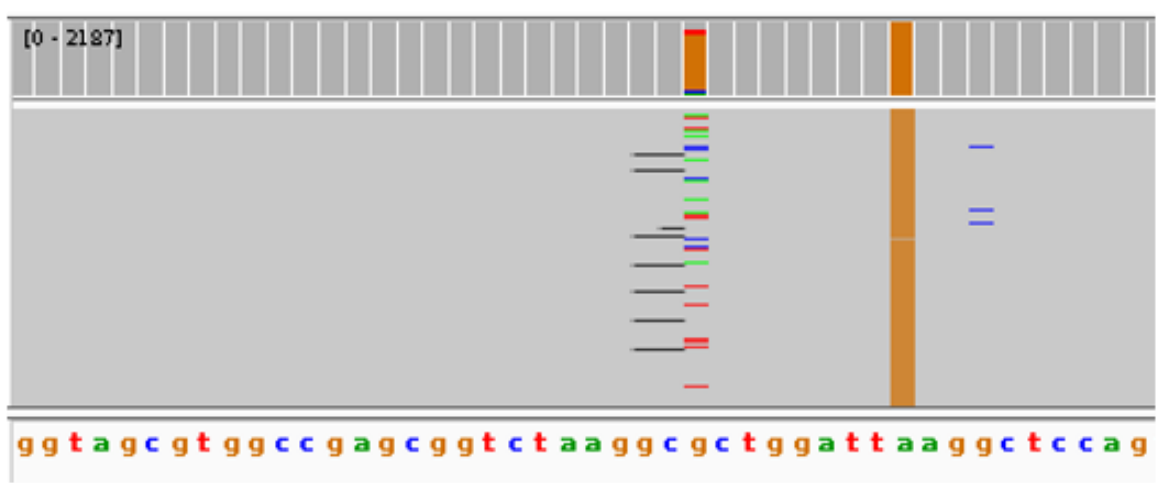

D Mt-Val-TAC (cluster338)

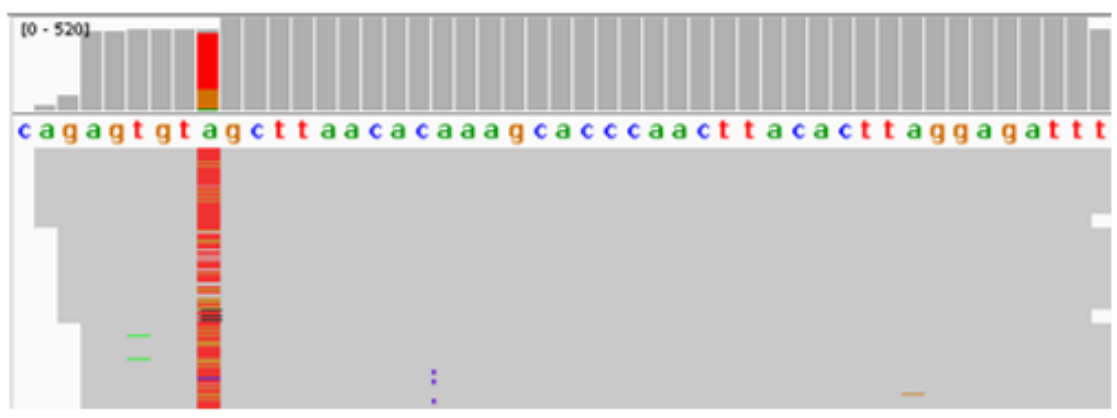

E

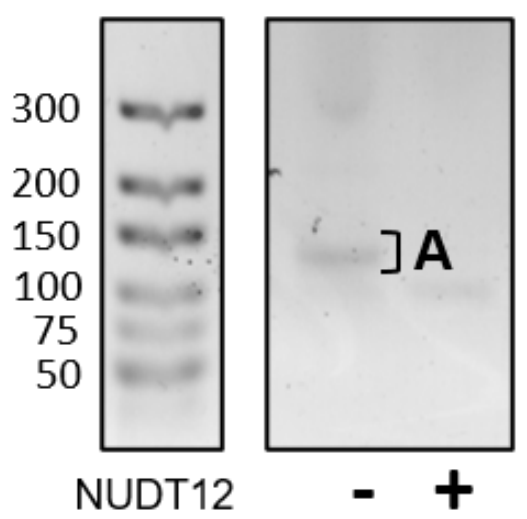

F Leu-AAG 1-1 from short RNA – Band A

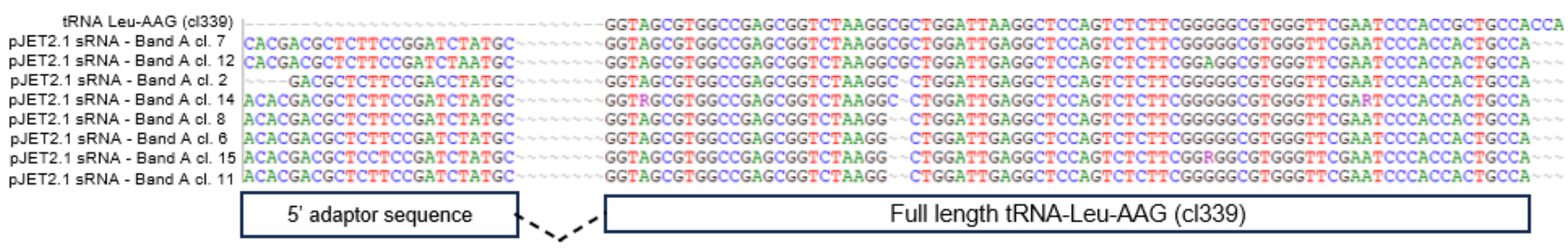

Figure S17: ONT direct RNA-seq analysis of the deacylated tRNA$^{Leu\text{-}AAG}$ pull-down.
A) The distribution of the read-lengths in the sequencing data after filtering (QS>4, minimal length 30). **B)** The composition of the pull-down RNA expressed as percentage of the indicated RNA species within the total mapped reads. **C)** Representative IGV tracks of tRNA$^{Leu\text{-}AAG}$ from the Ap$_2$N-seq (-NUDT12), showing the presence of majority of full-length tRNAs (adaptor attached to the G-base at position 1). Examples of individual reads are shown below the coverage and sequence track, each line representing one read. The tRNA is not shown in its full length. **D)** Representative IGV tracks of major contaminating tRNA$^{Mt\text{-}Val\text{-}TAC}$ from the Ap$_2$N Seq (-NUDT12). Most of the bases immediately 3' to the ligated adaptor are the adenosines mapping to position 4 of the sequence (See Figure S14 J for more details),

contributing to the A in the LC-MS signal detected in $tRNA^{Leu\text{-}AAG}$ pull-down. **E–F)** Orthogonal validation of $tRNA^{Leu\text{-}AAG}$ capture. **E)** Agarose gel showing PCR products generated using a 5′ adaptor forward primer and a $tRNA^{Leu\text{-}AAG}$–specific reverse primer from short RNA processed by the Ap_2N-seq protocol up to and including the adaptor-ligation step. The band excised for cloning and Sanger sequencing is marked A. **F)** Alignment of Sanger-sequenced inserts to the $tRNA^{Leu\text{-}AAG}$ reference sequence. For clarity, sequences are shown as the 5′ adaptor segment followed by the immediately adjacent $tRNA^{Leu\text{-}AAG}$ sequence. The wobble position A34 of $tRNA^{Leu\text{-}AAG}$ is known to be modified to inosine (I34); because inosine is typically read as G during reverse transcription, this accounts for the consistent A→G transition observed at this position in the Sanger reads.

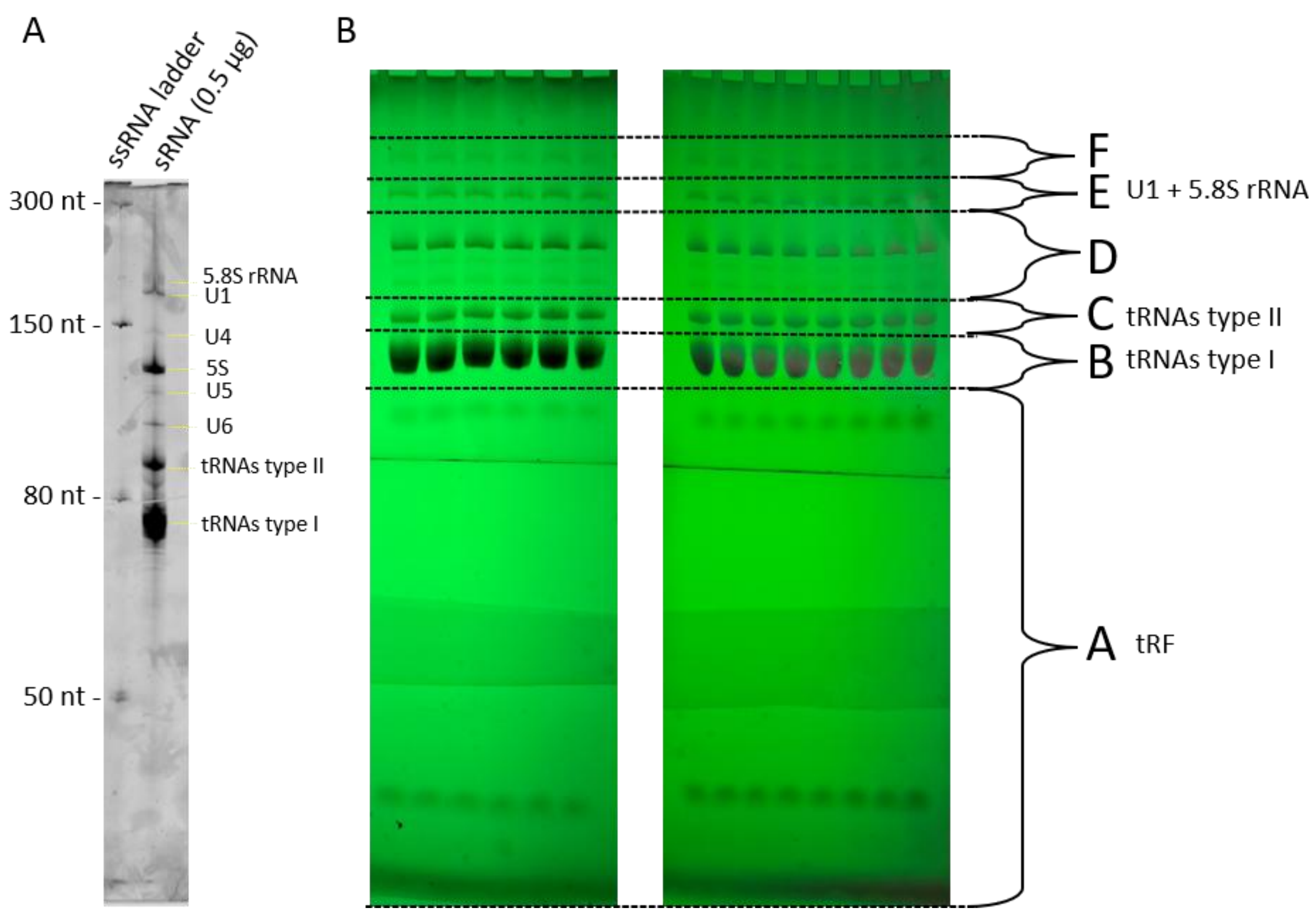

Figure S18: sRNA resolution isolated from Expi293F control cells on 10% denaturing PAGE. A) Illustrative sRNA (0.5 ug) separation on 10% PAGE visualized by SYBR Gold staining showing position of the main bands compared to commercial ssRNA ladder (New England Biolabs). Assignment was done based on ref. (21-23) **B)** sRNA (1.6 mg in total) separation on 10% PAGE visualized by UV-shadowing on fluorescence silica TLC plates. Gel portions that were cut for RNA extraction are indicated.

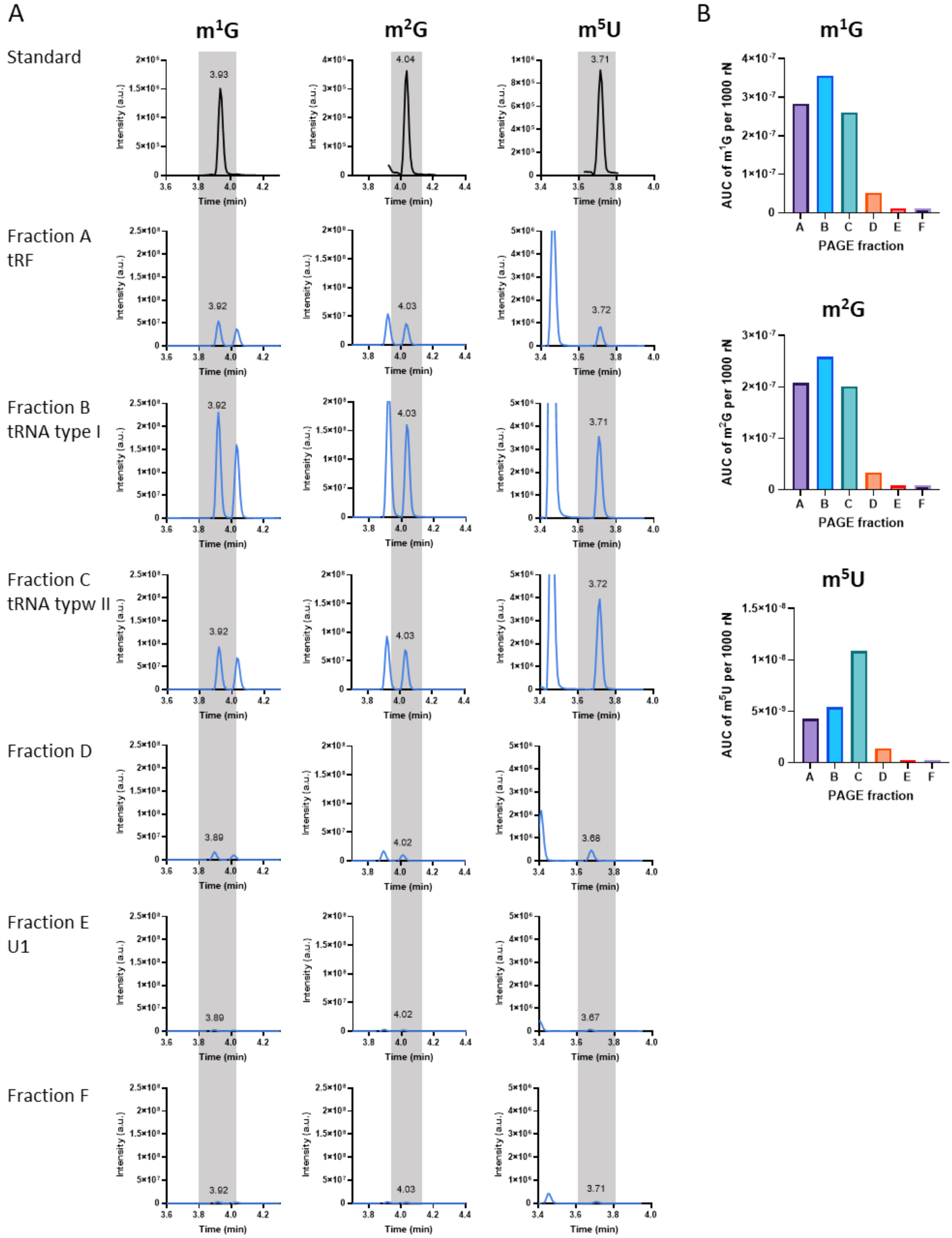

Figure S19: LC–MS analysis of internal RNA modifications in fractions obtained from sRNA fractionation on 10% PAGE. A) MRM chromatograms show m^{1}G (MRM transition 298->150), m^{2}G (MRM transition 298->166) and m^{5}U (MRM transition 259->127) standards (black line) and corresponding modifications (blue line). **B)** Relative quantification of detected modifications in all fractions (1000 rN=1000 canonical nucleosides).

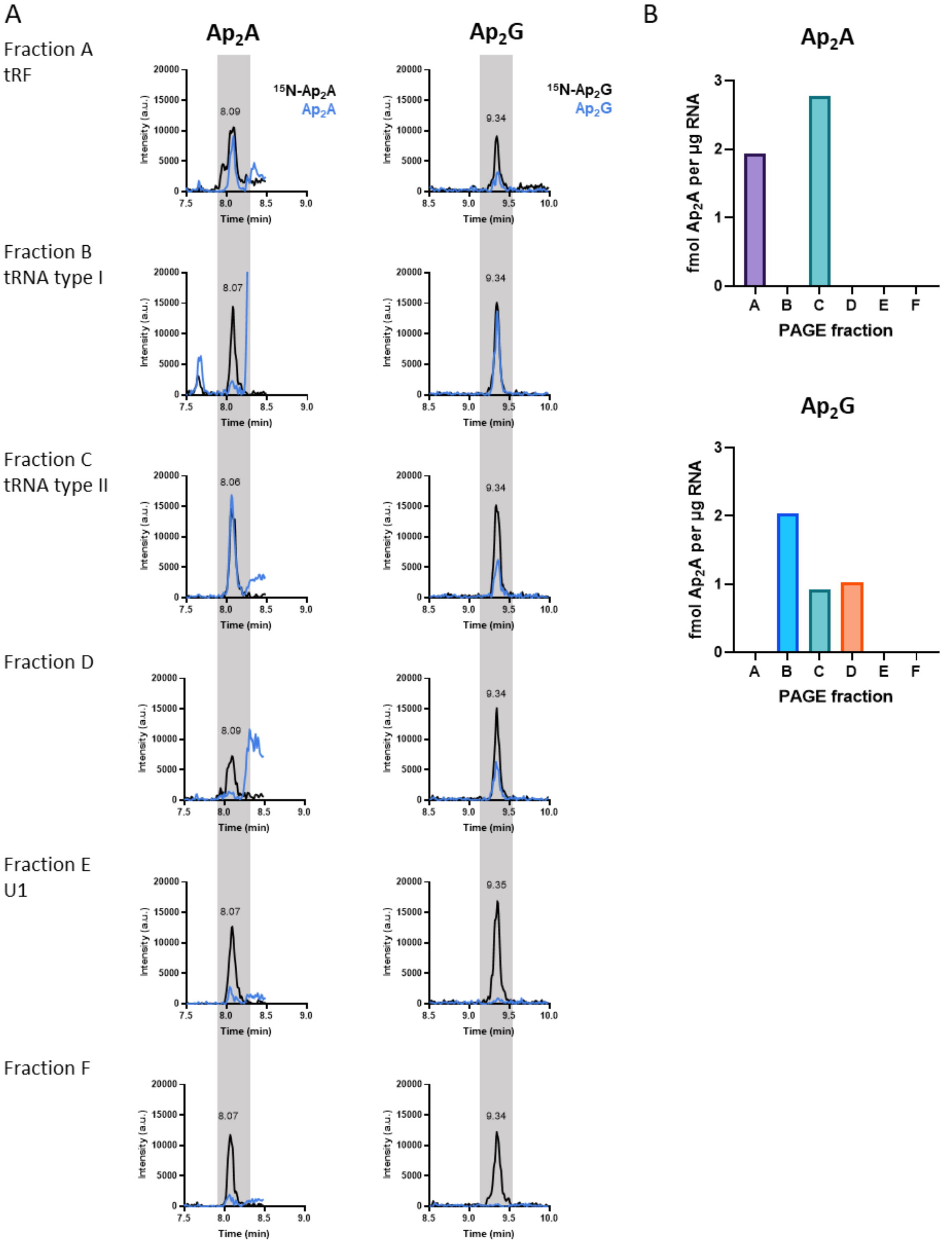

Figure S20: LC–MS analysis of Ap_2A and Ap_2G RNA caps in fractions obtained from sRNA fractionation on 10% PAGE. A) MRM chromatograms show the spiked ^{15}N-Ap_2A (MRM transition 687->255) and ^{15}N-Ap_2G (MRM transition 698->433) standards (black line) and corresponding caps (MRM transitions 677->250 and 693->428 for Ap_2A and Ap_2G, respectively) (blue line). **B)** Quantification of Ap_2A and Ap_2G RNA caps in all fractions.

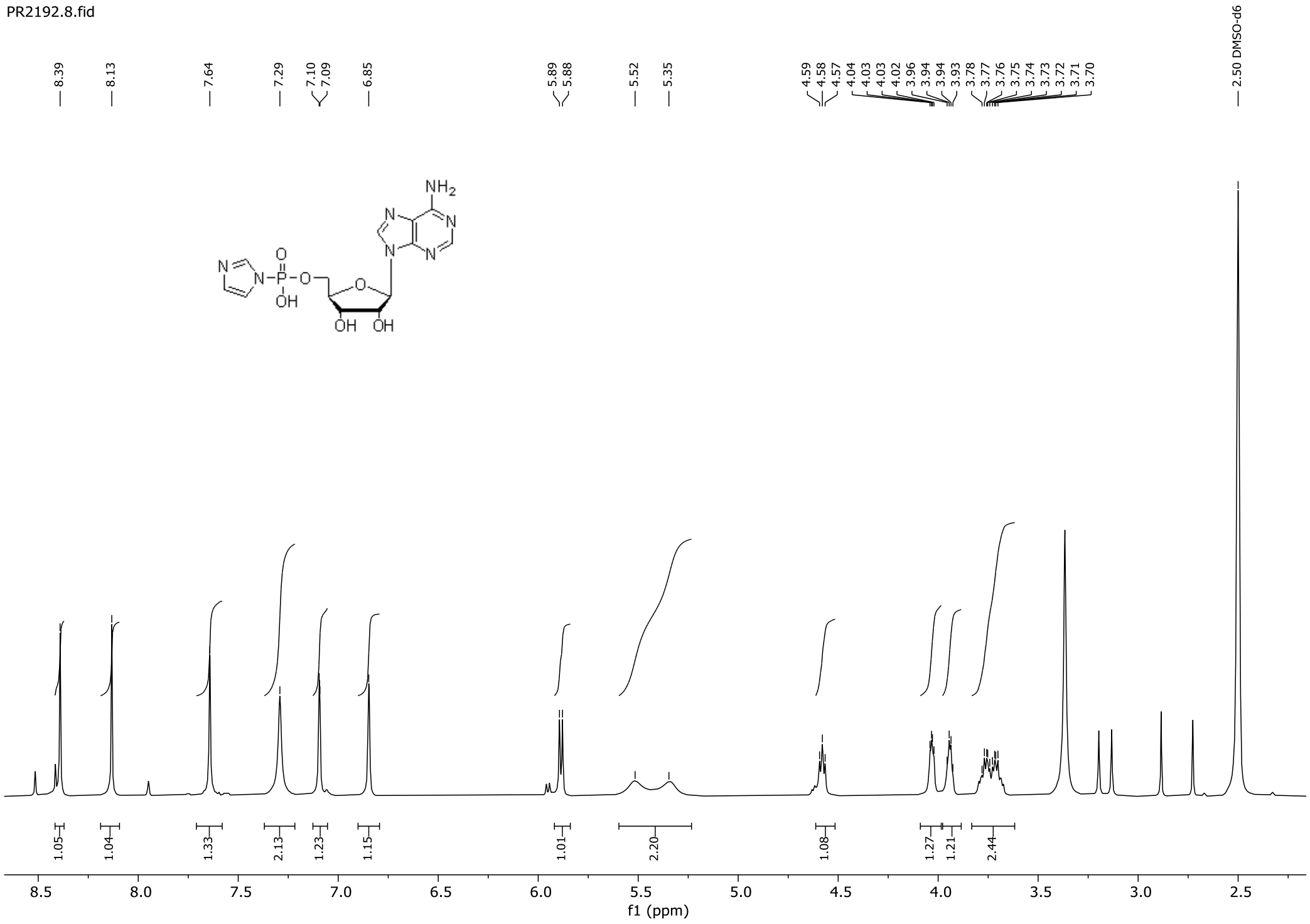

Figure S21: ^{1}H NMR spectrum of adenosine 5′-phosphoimidazolide

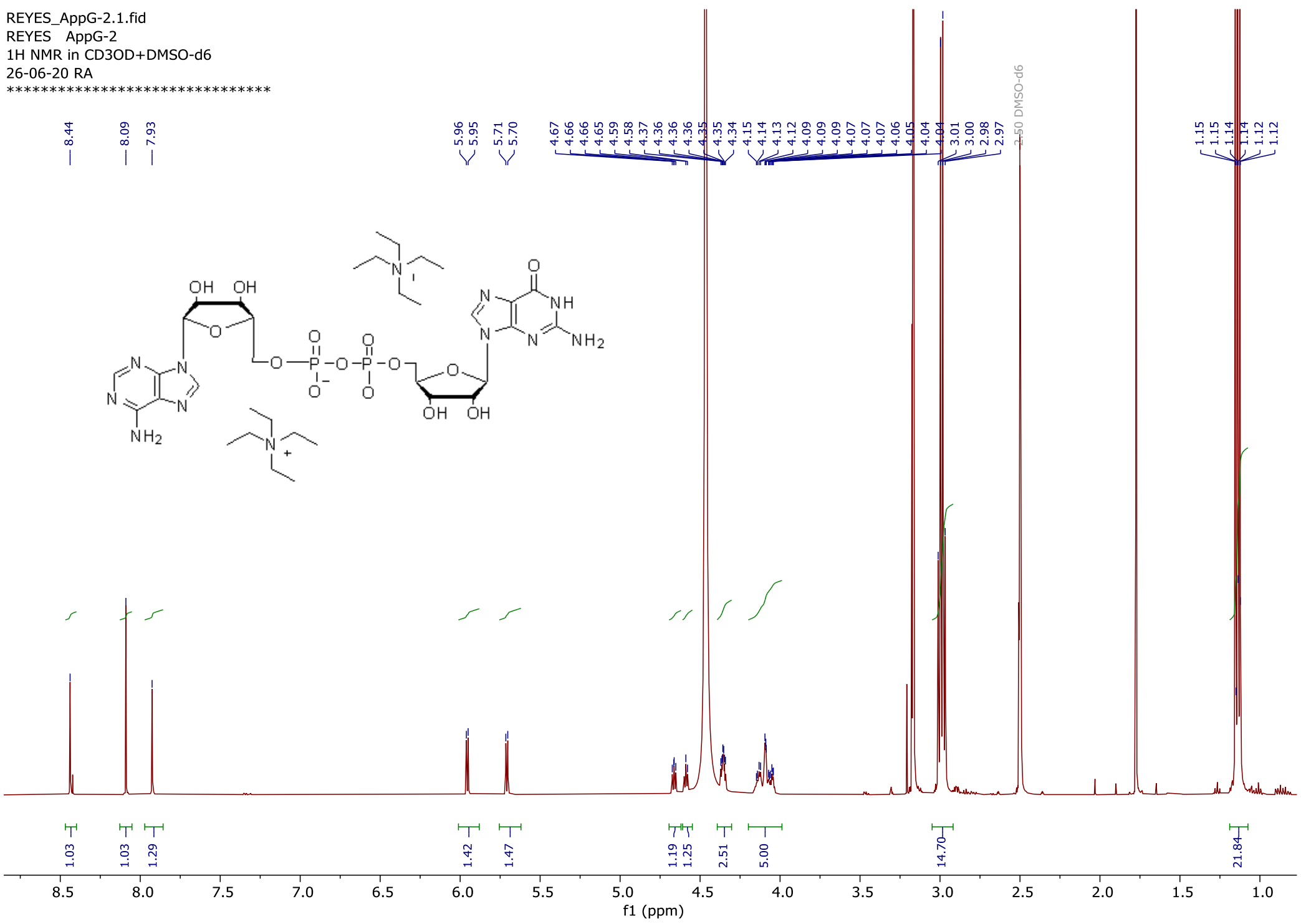

Figure S22: ^{1}H NMR spectrum of P1-(5'-adenosyl)-P2-(5'-guanosyl) diphosphate (Ap_2G)

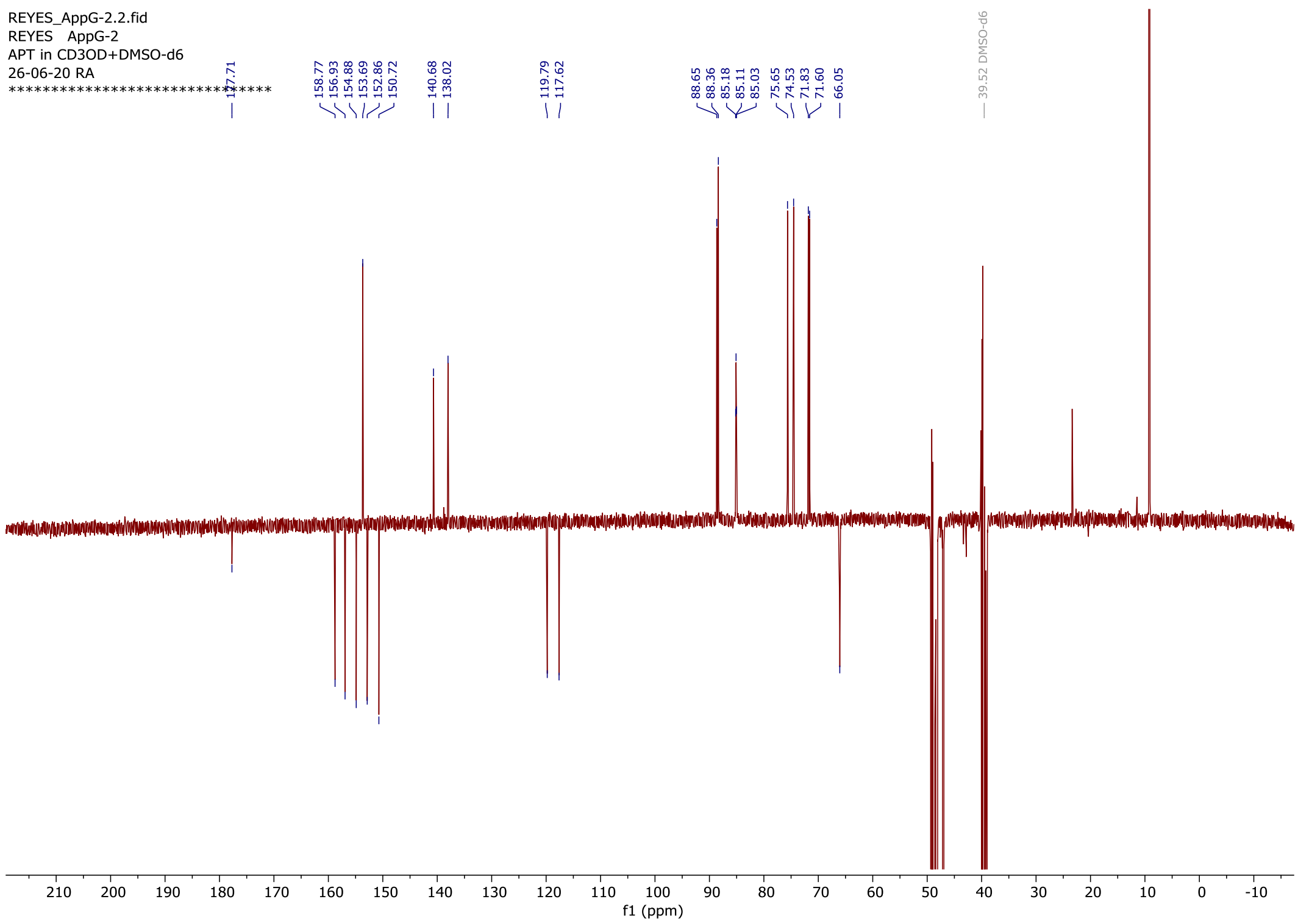

Figure S23: ^{13}C NMR spectrum of P1-(5'-adenosyl)-P2-(5'-guanosyl) diphosphate (Ap_2G)

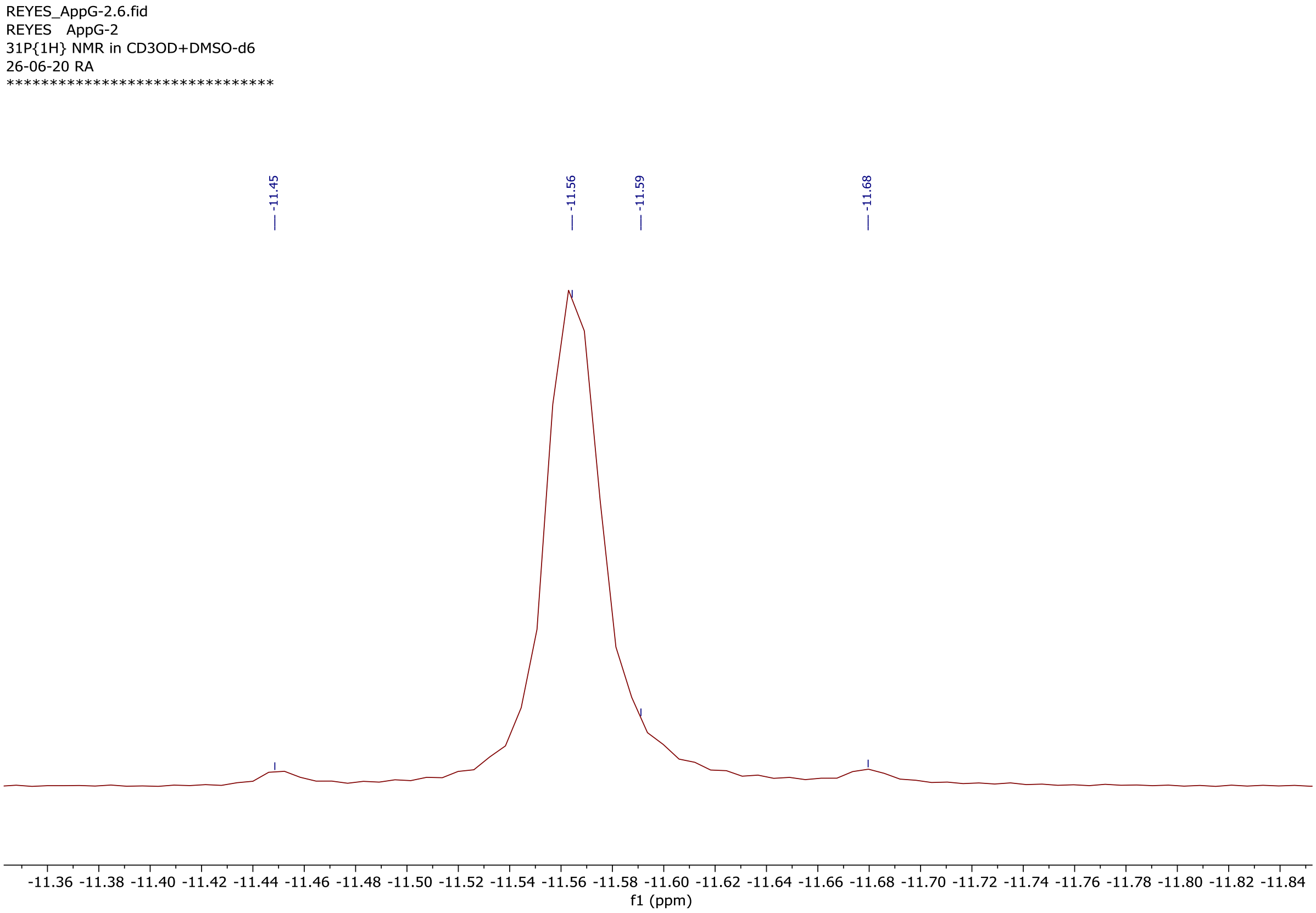

Figure S24: ^{31}P NMR of P1-(5'-adenosyl)-P2-(5'-guanosyl) diphosphate (Ap_2G)

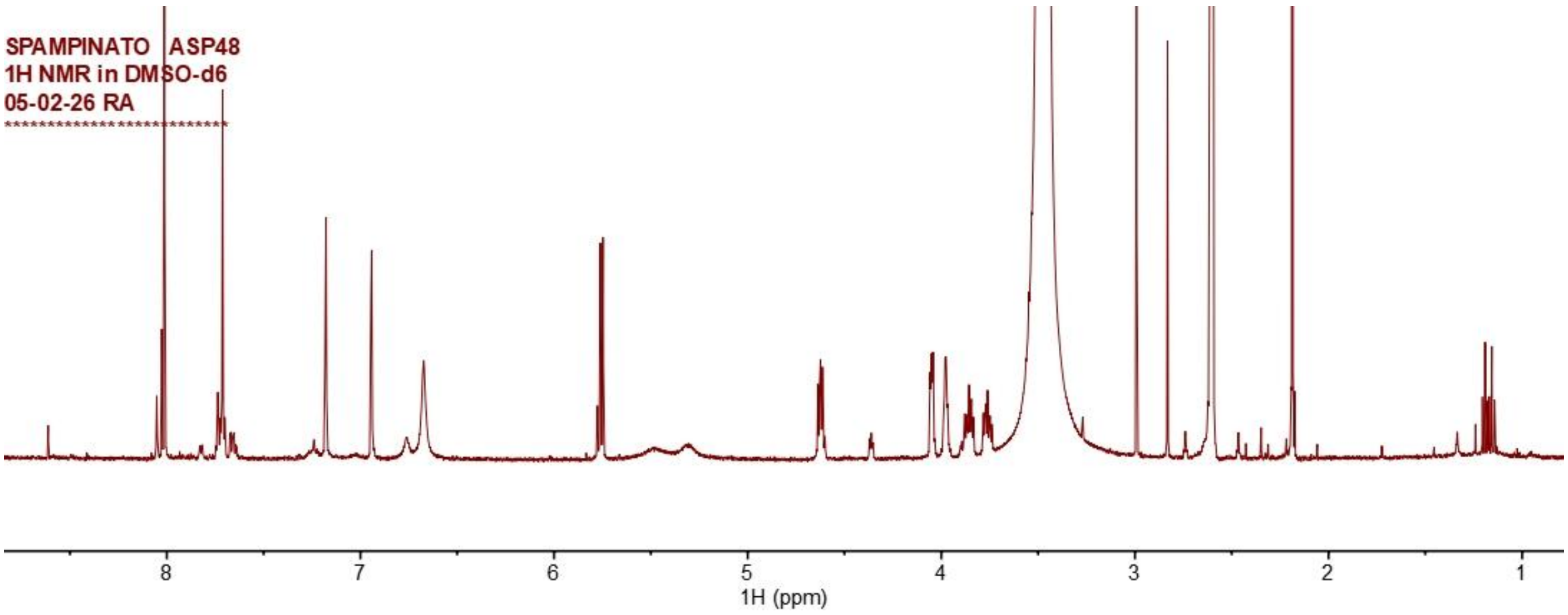

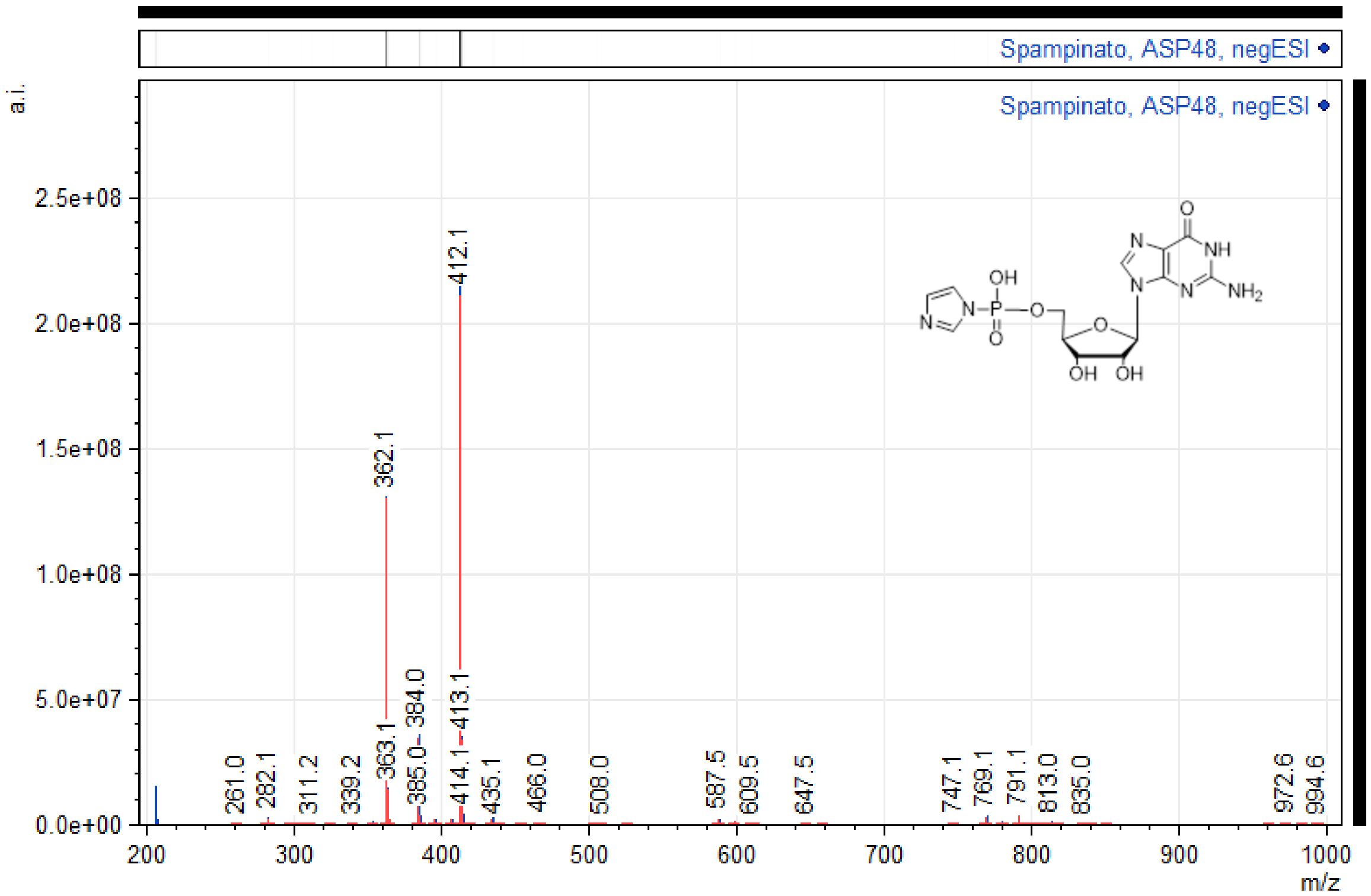

Figure S25: ^{1}H NMR and MS analysis of ImpG and GMP mixture. HR/MS (ESI-) for ImpG m/z 412.07748 [M-H]-.

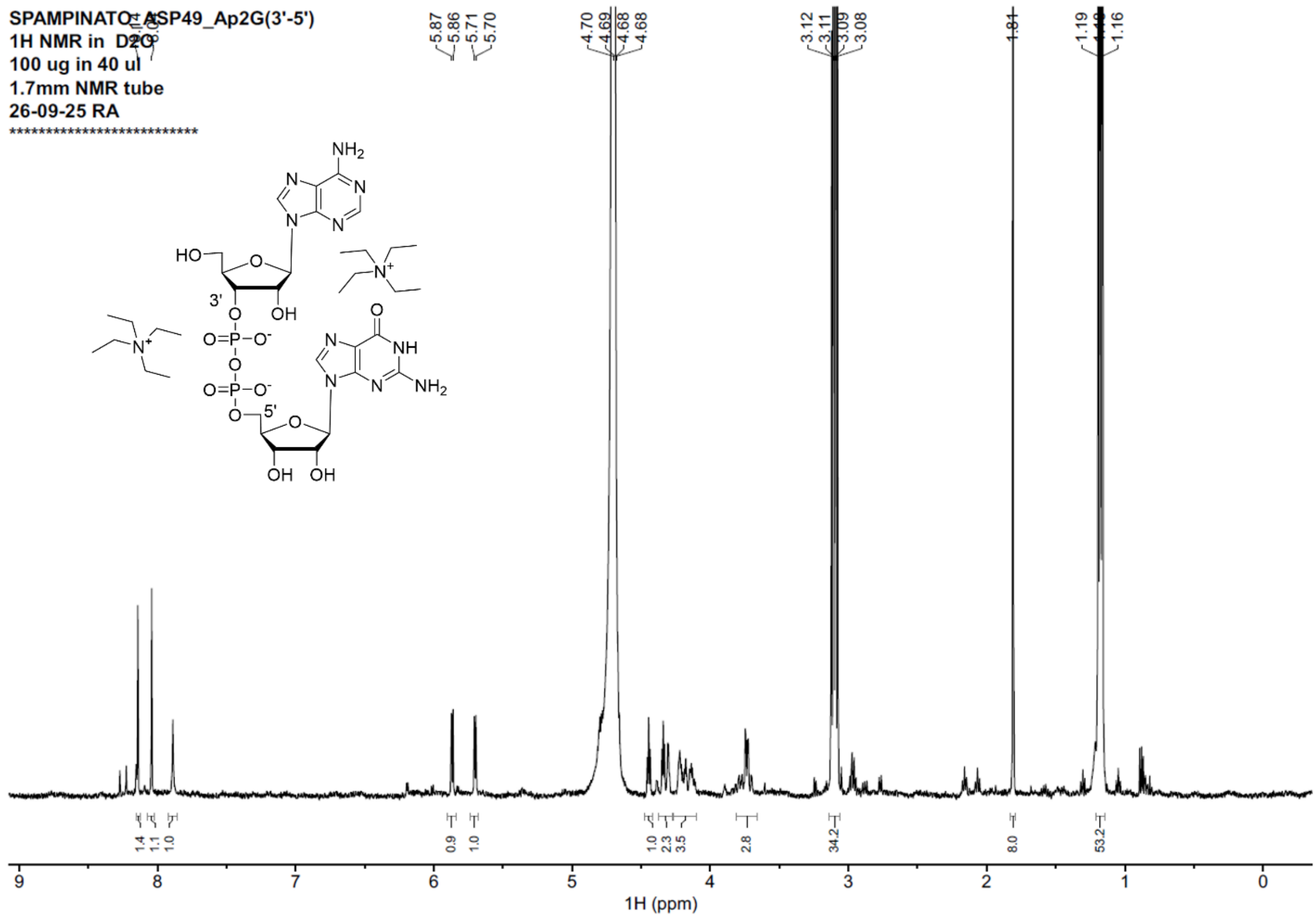

Figure S26: ^{1}H NMR spectrum of P1-(3'-adenosyl)-P2-(5'-guanosyl) diphosphate Ap$_2$G (3'-5').

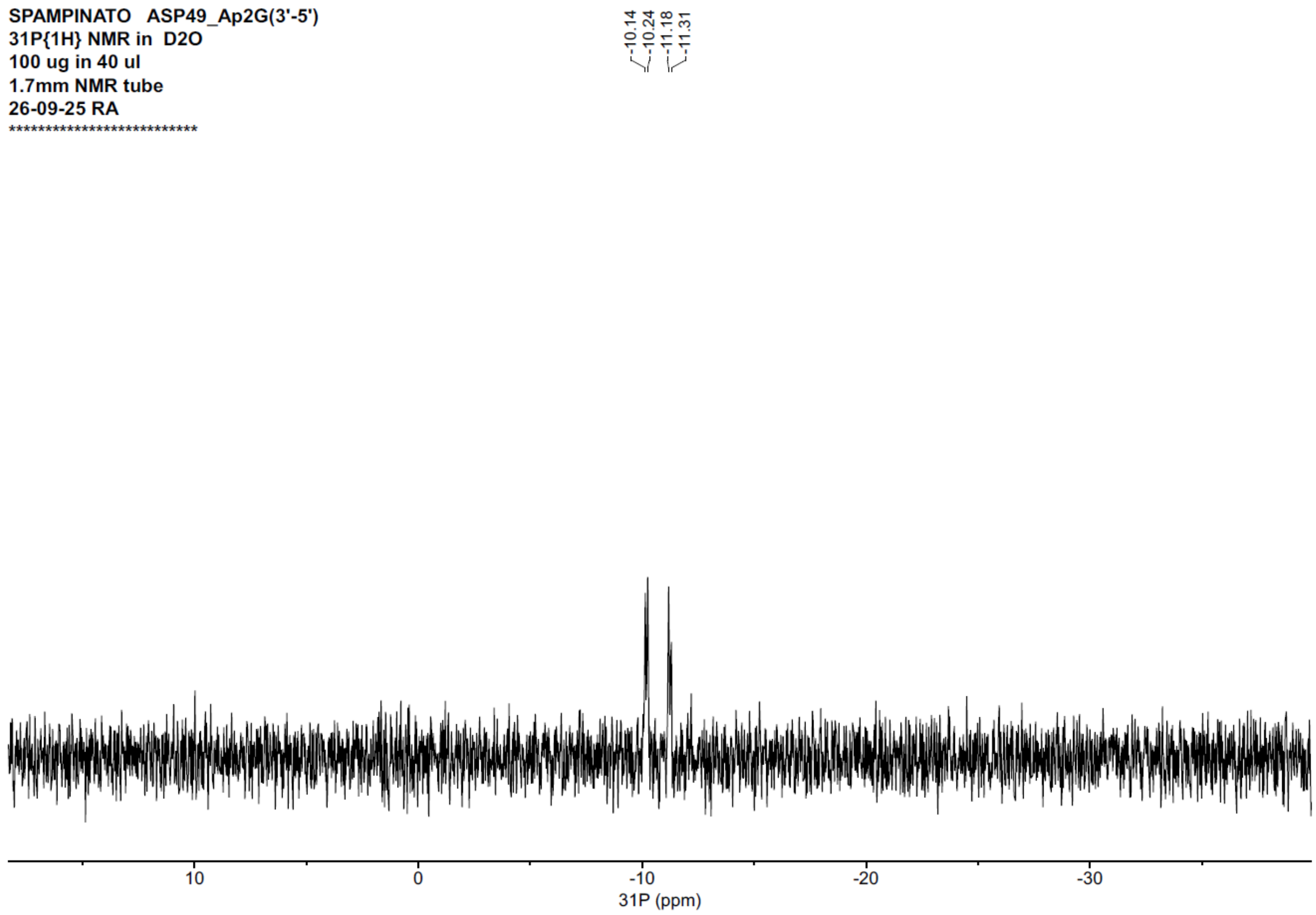

Figure S27: ^{31}P NMR spectrum of P1-(3'-adenosyl)-P2-(5'-guanosyl) diphosphate Ap$_2$G (3'-5').

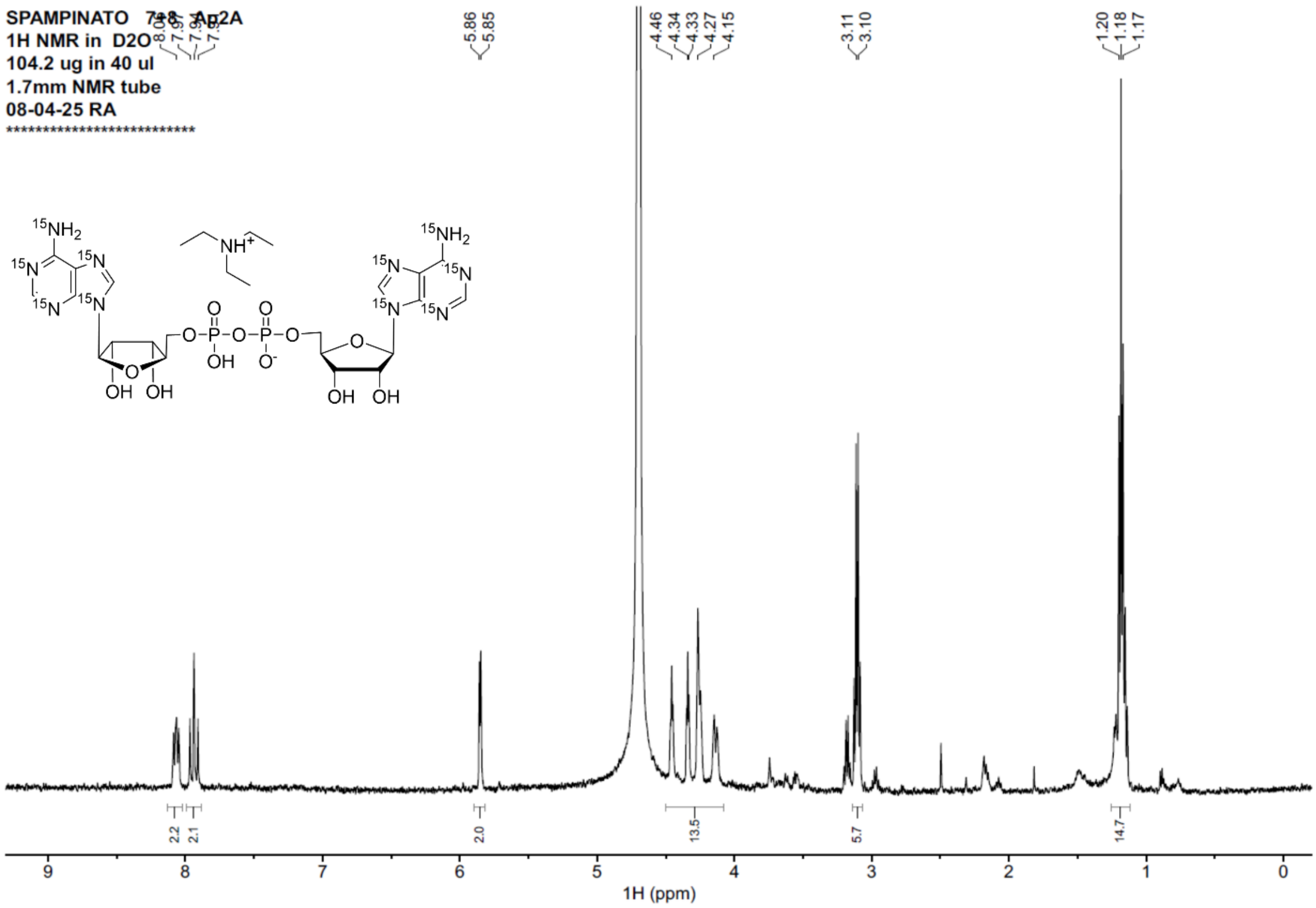

Figure S28: ^{1}H NMR spectrum of P1-(5'-^{15}N-adenosyl)-P2-(5'-^{15}N-adenosyl) diphosphate (^{15}N-Ap$_2$A).

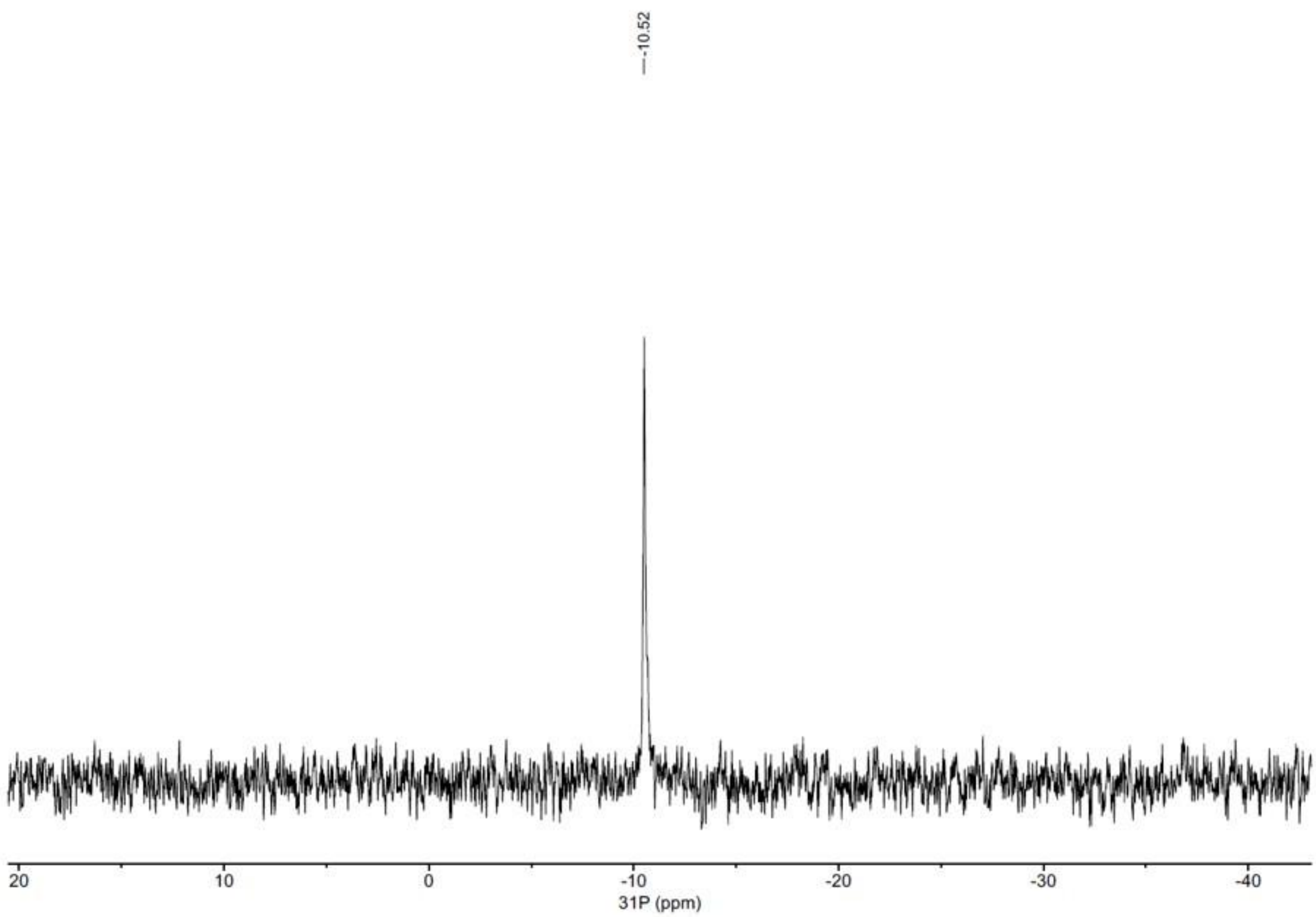

Figure S29: ^{31}P NMR spectrum of P1-(5'-^{15}N-adenosyl)-P2-(5'-^{15}N-adenosyl) diphosphate (^{15}N-Ap$_2$A).

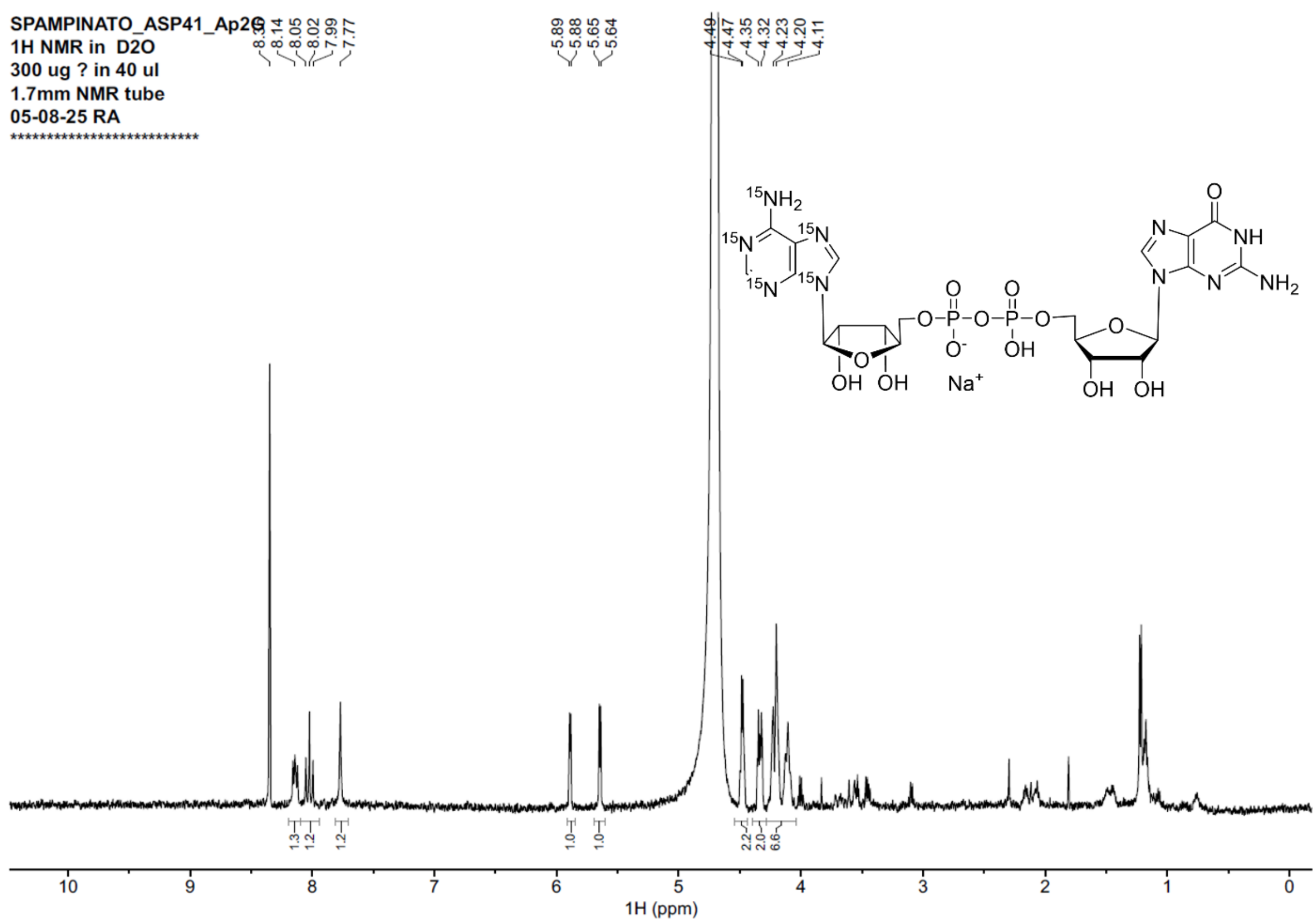

Figure S30: **^{1}H NMR spectrum of P1-(5'-^{15}N-adenosyl)-P2-(5'-guanosyl) diphosphate (^{15}N_-Ap$_2$G).**

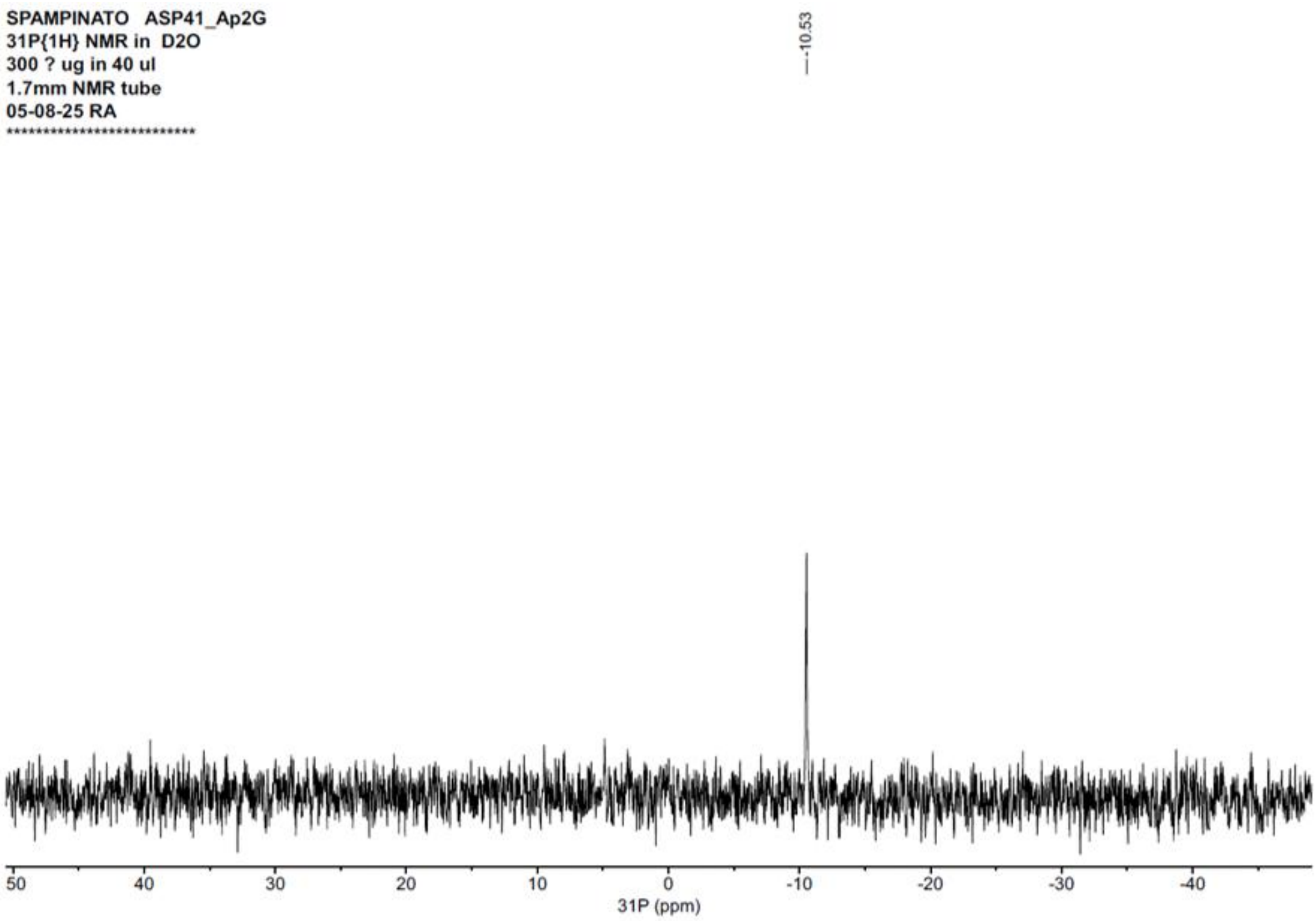

Figure S31: ^{31}P NMR spectrum of P1-(5'-^{15}N-adenosyl)-P2-(5'-guanosyl) diphosphate (^{15}N -Ap$_2$G).

Uncropped original gels:

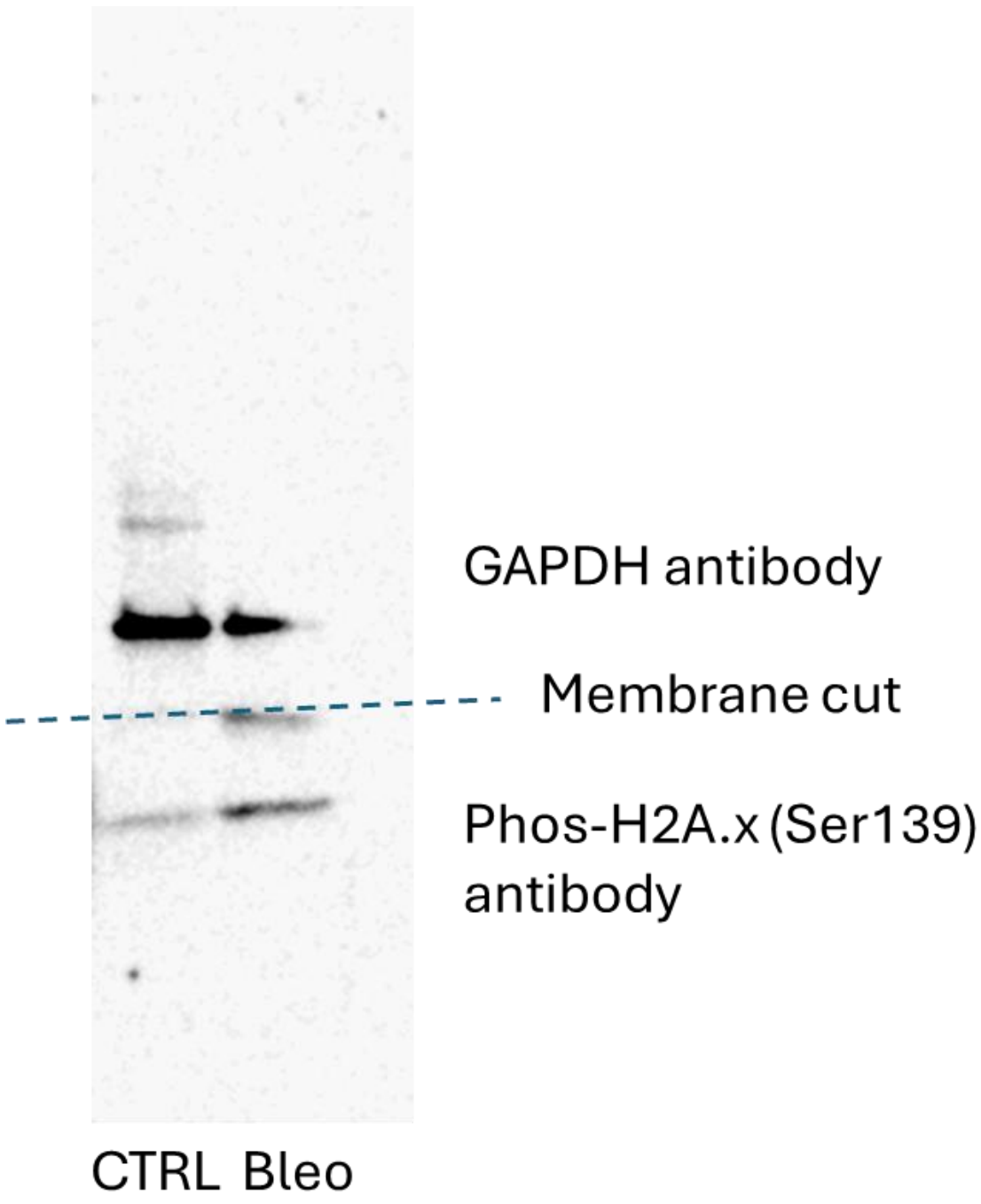

Figure S32: Uncropped western blot membrane related to Bleomycine stress in Figure S4.

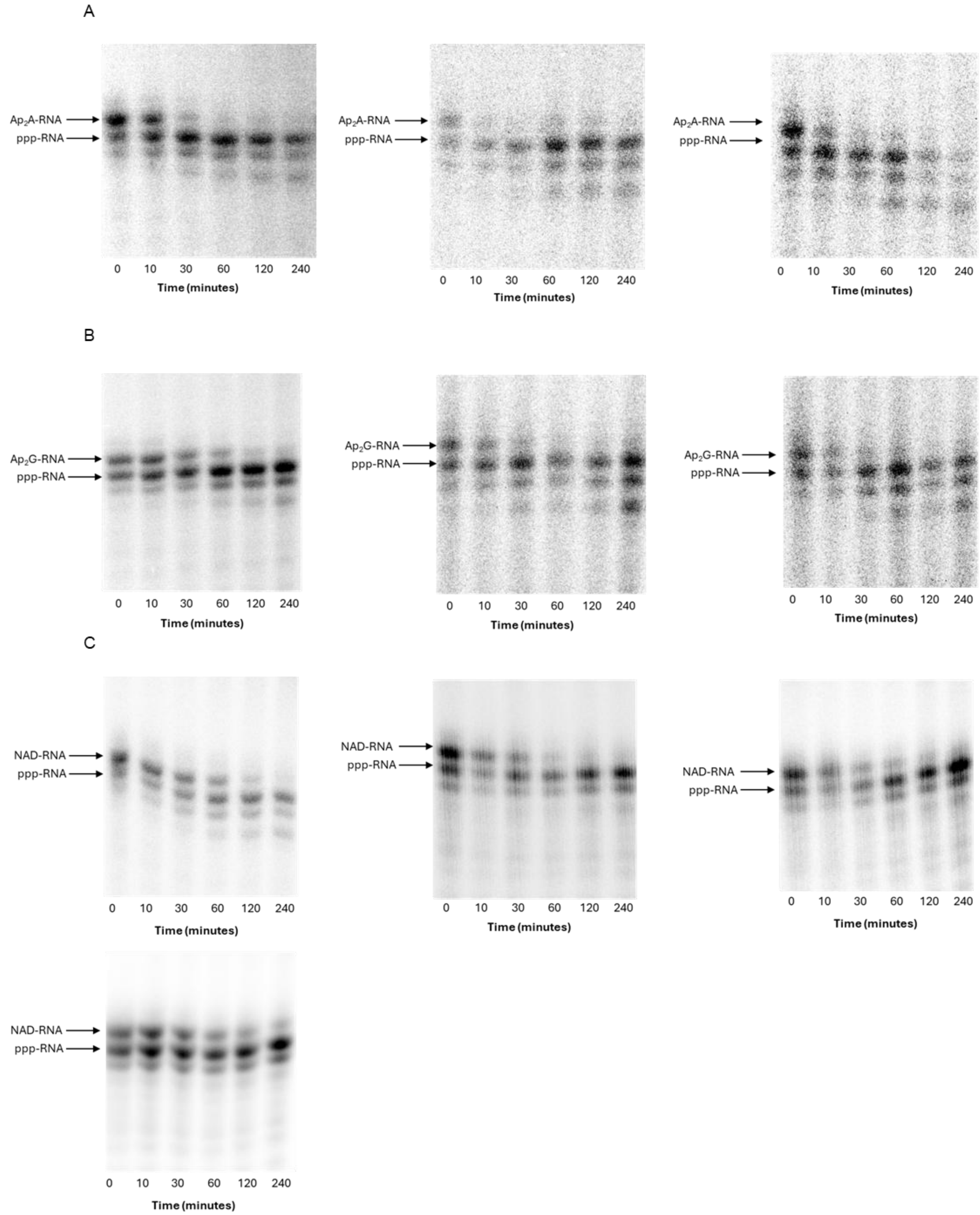

Figure S33: The original uncropped gels used for calculation of NUDT12 decapping of Ap_2A-RNA (A), Ap_2G-RNA (B) and NAD-RNA (C) shown in Figure 2B.

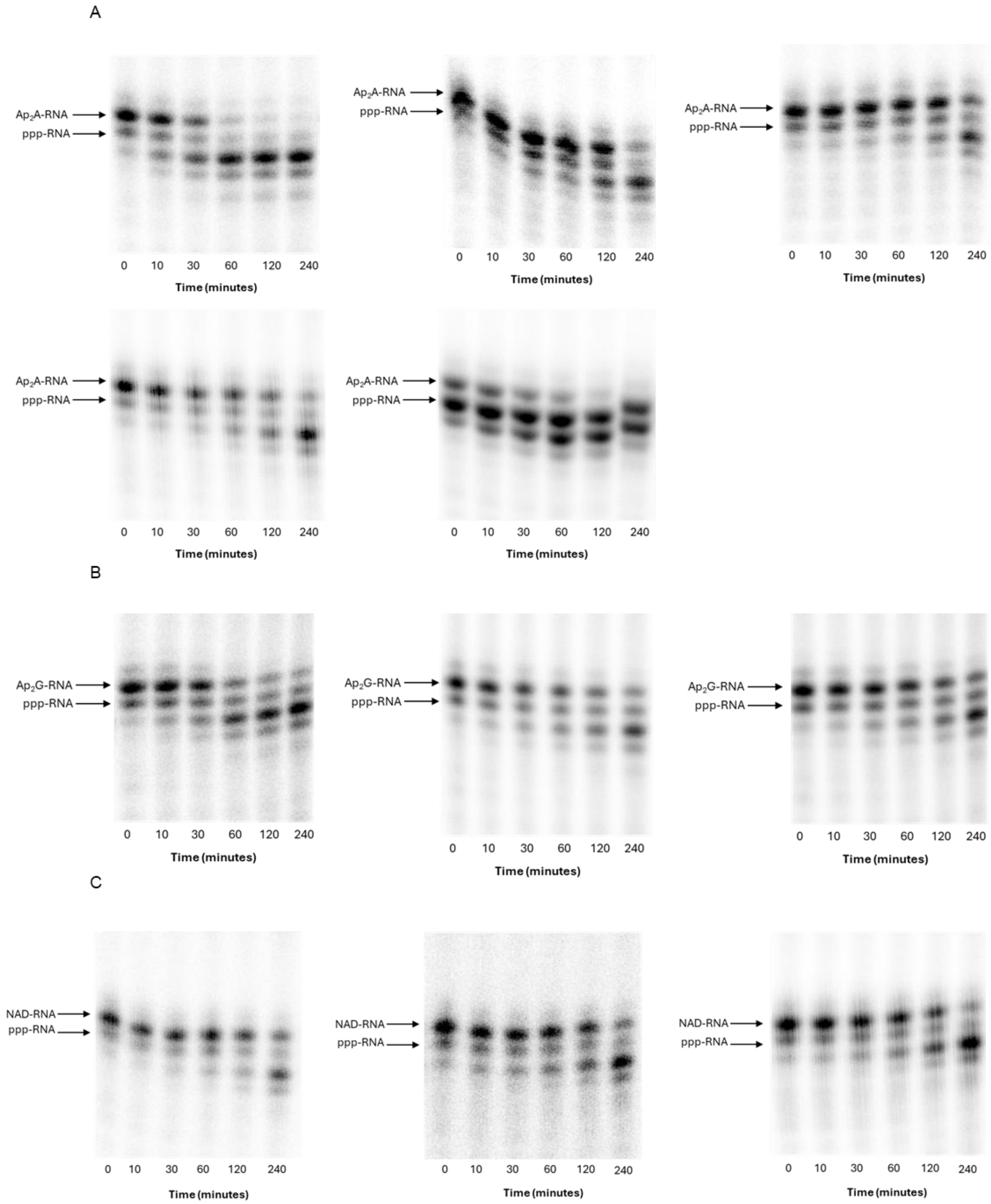

Figure S34: The original uncropped gels used for calculation of DXO decapping of Ap_2A-RNA (A), Ap_2G-RNA (B) and NAD-RNA (C) shown in Figure S6.

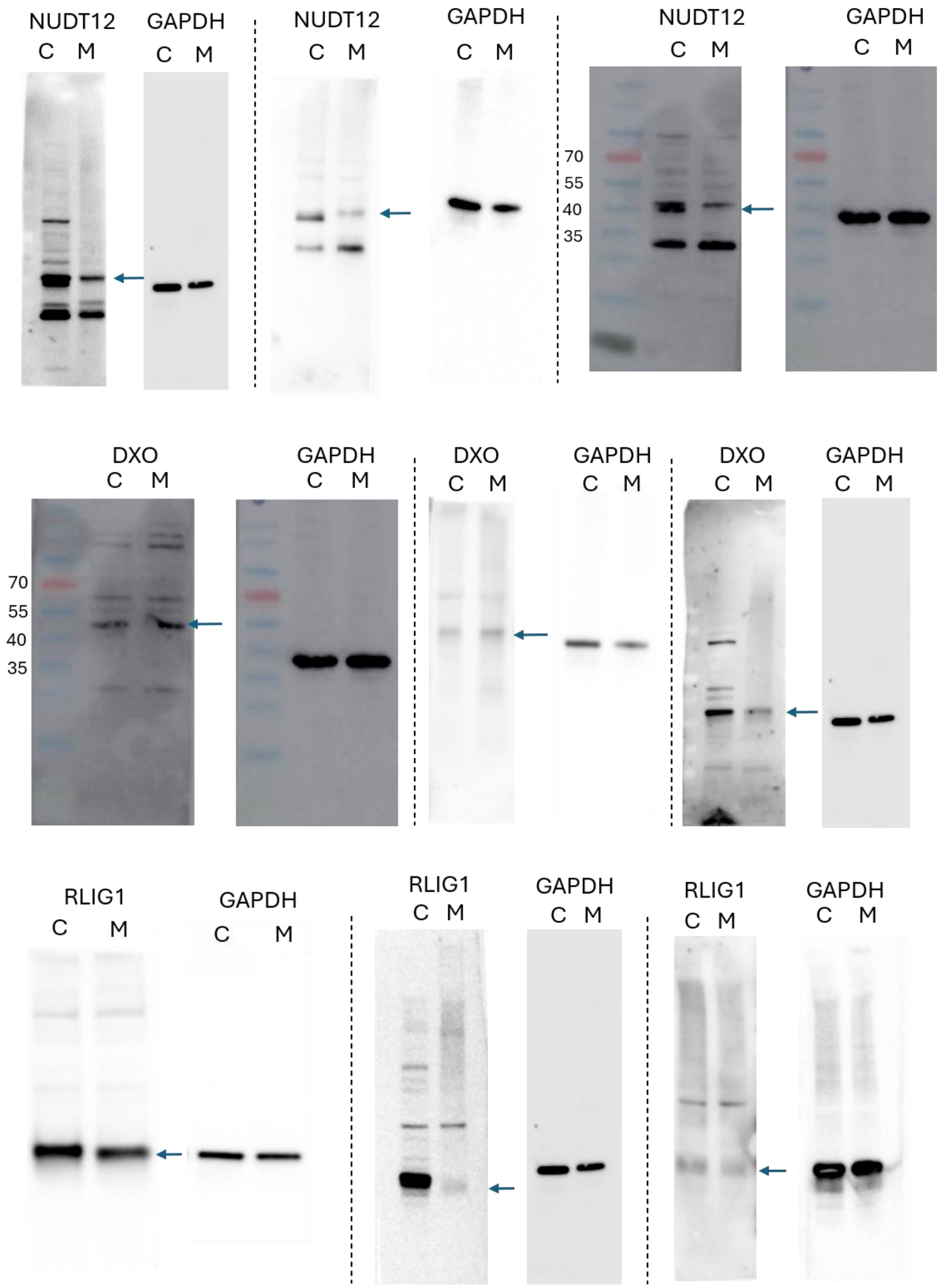

Figure S35: Uncropped western blot membranes of decapping enzymes and RLIG1 in control (c) and menadione treated cells (M) in Figure S8.

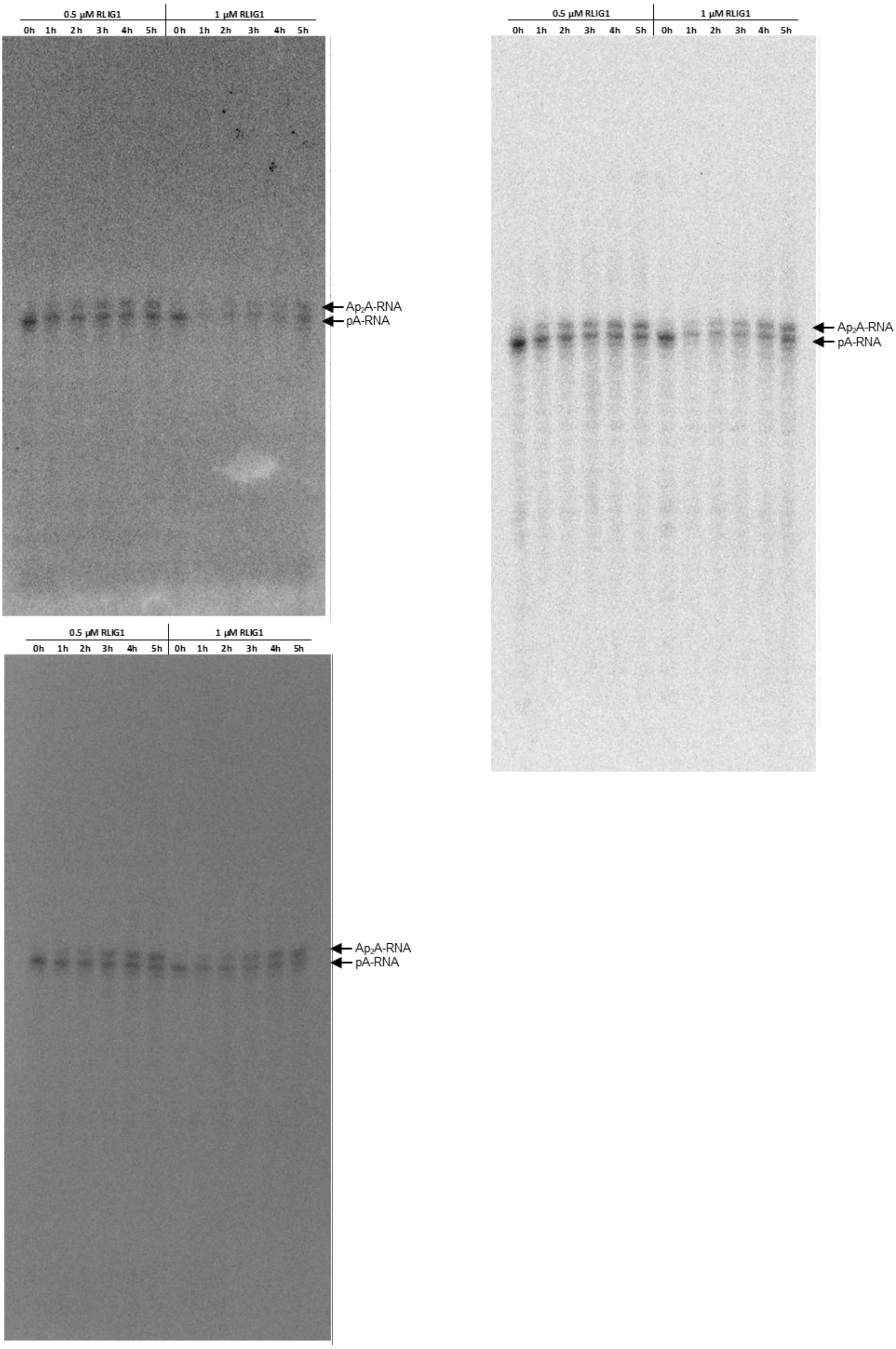

Figure S36: Uncropped gels used for quantification in Figure 2C - formation of Ap$_2$A-RNA from pA-RNA by RLIG1.

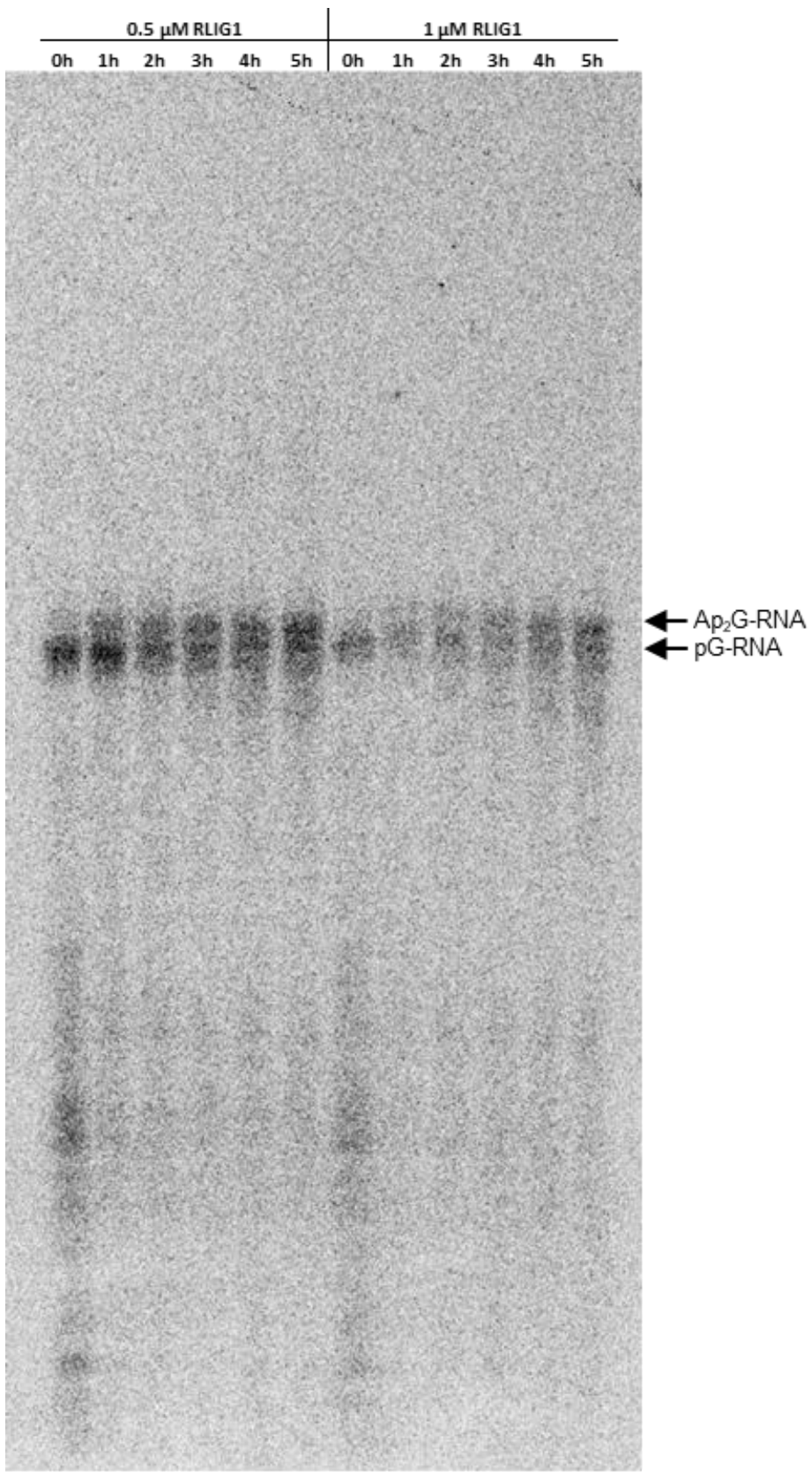

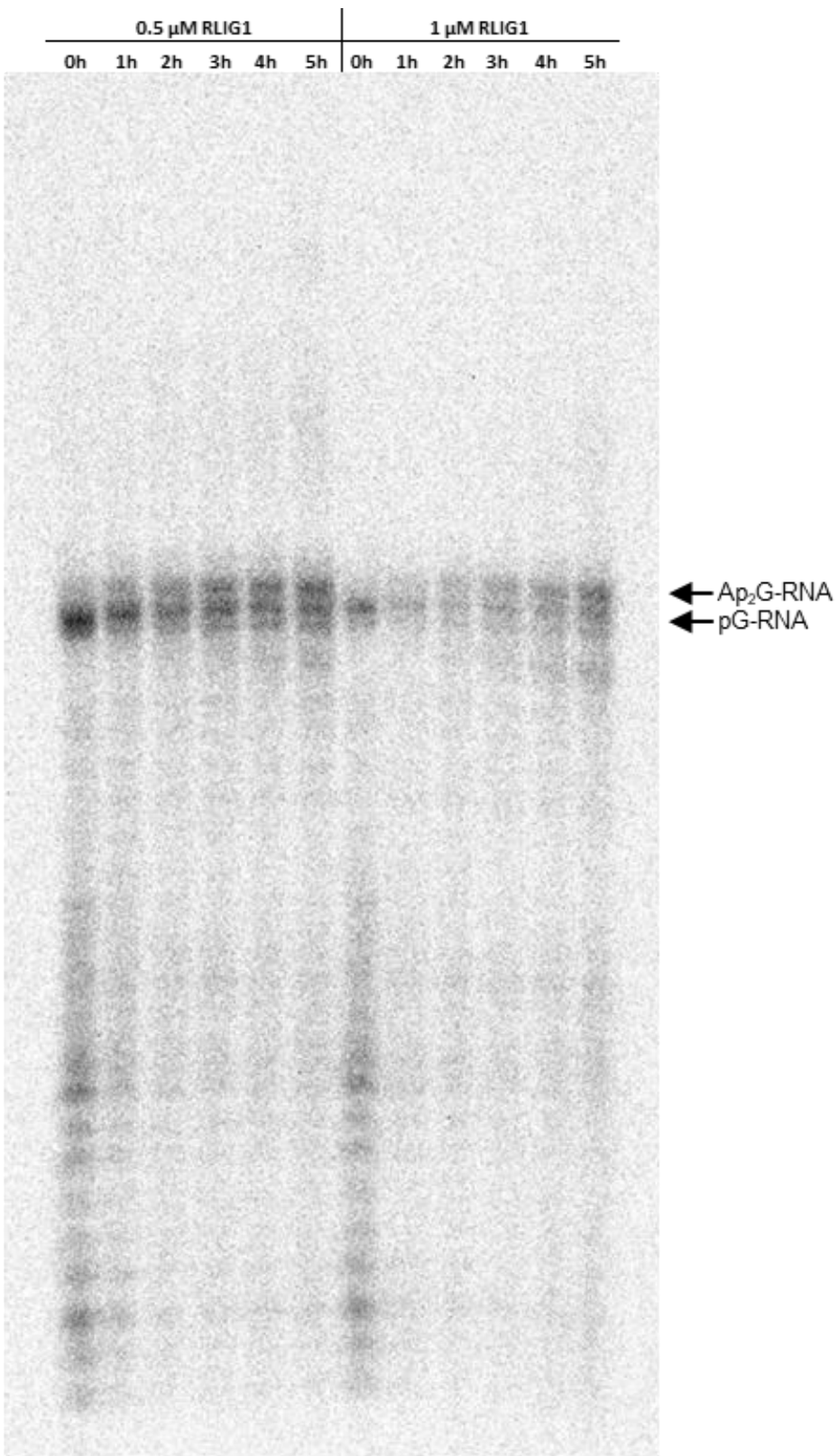

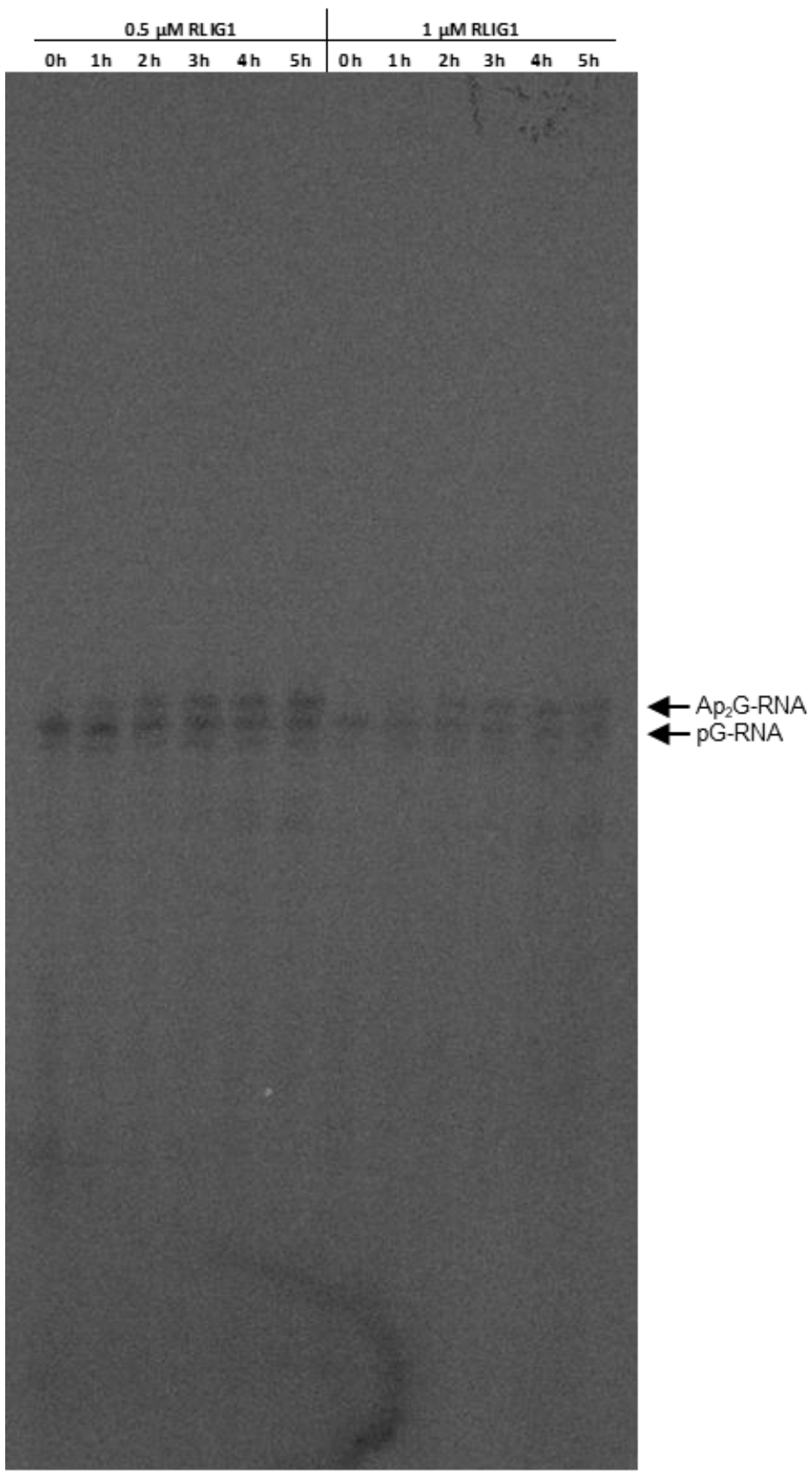

Figure S37: Uncropped gels used for quantification in Figure S10A - Formation of Ap_2G-RNA from pG-RNA by RLIG1.

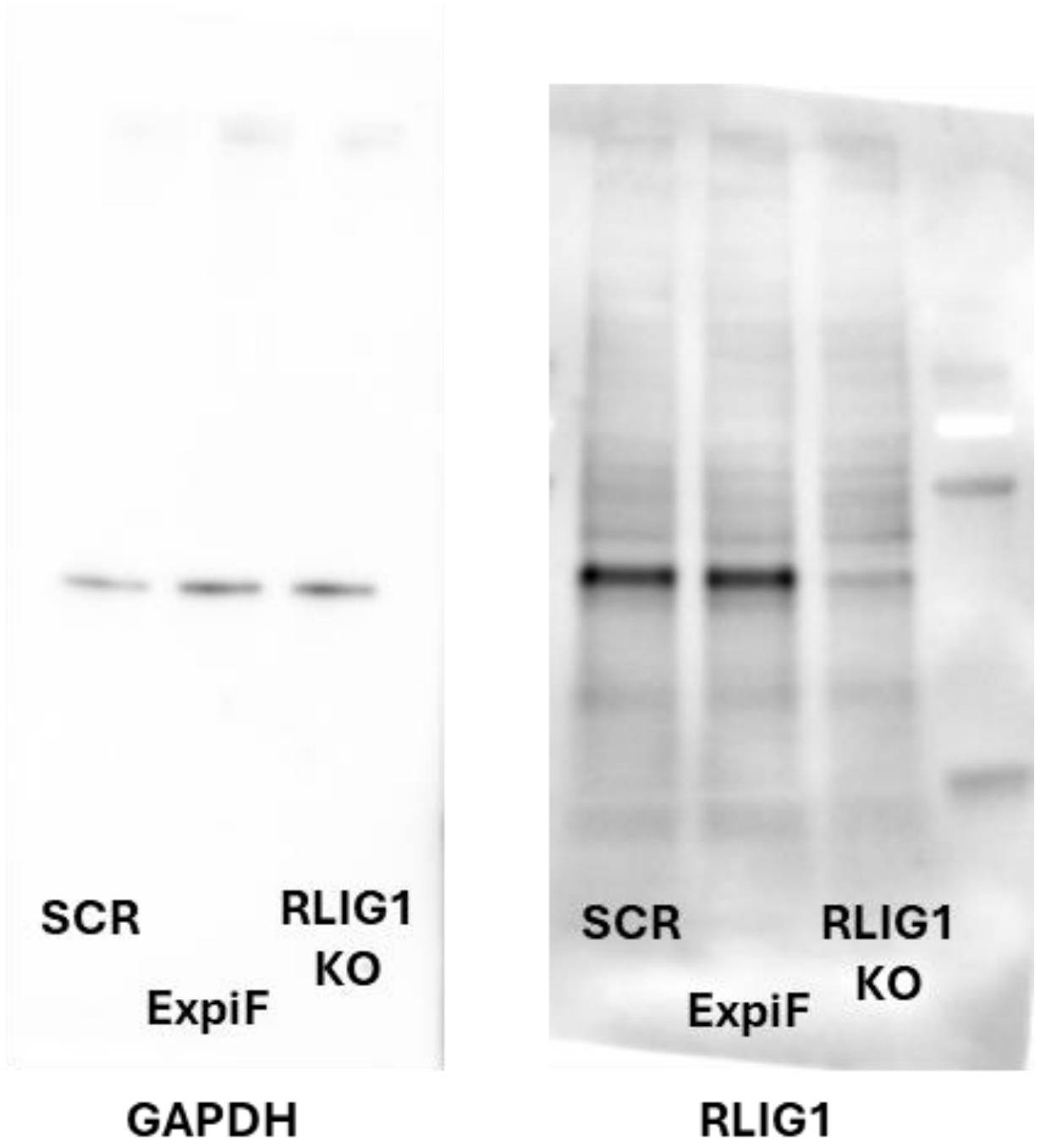

Figure S38: Uncropped membranes of Western Blot related to Figure 2D – characterization of RLIG1 knock-out line.

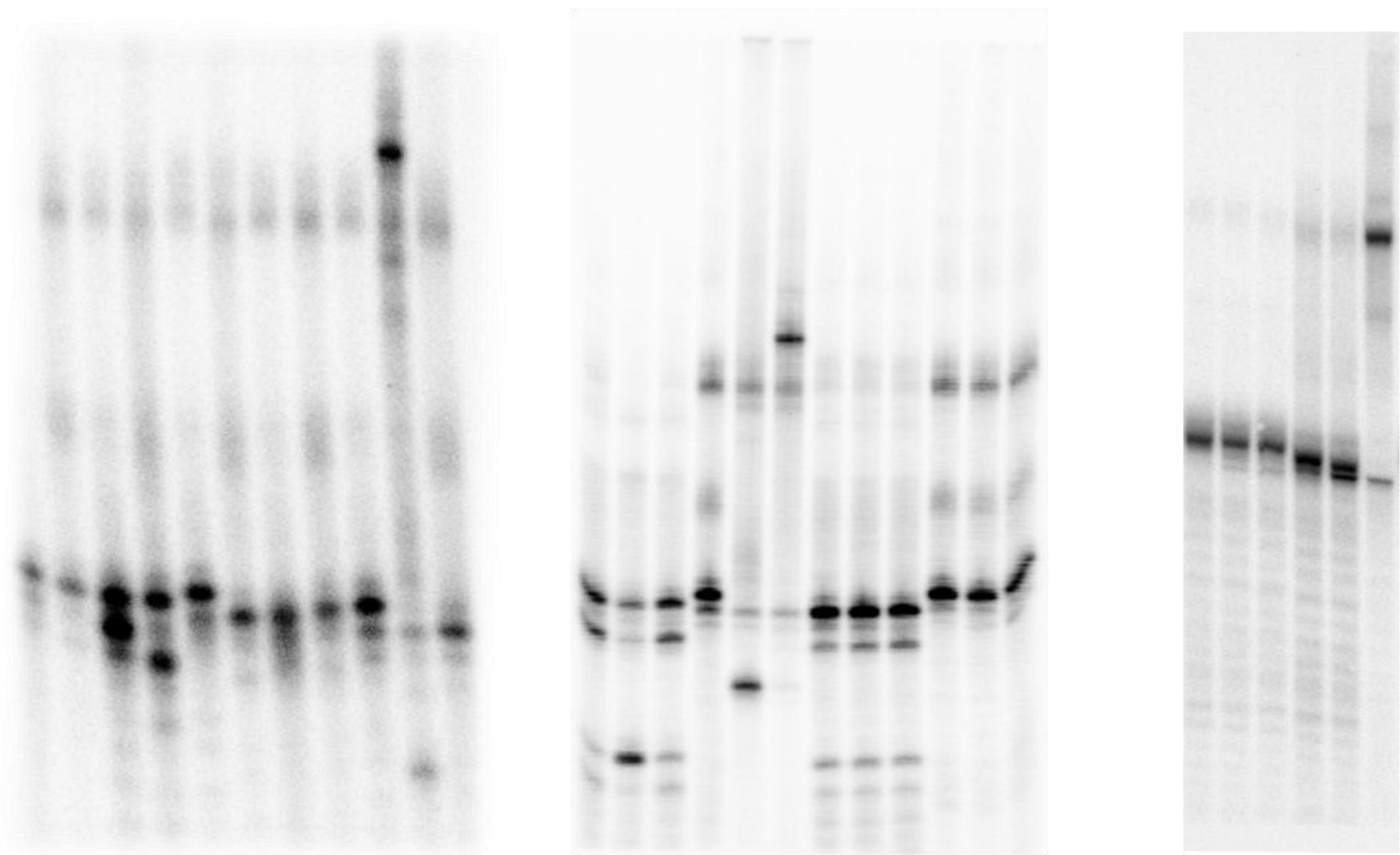

Figure S39: The original uncropped gel of Figure S12.

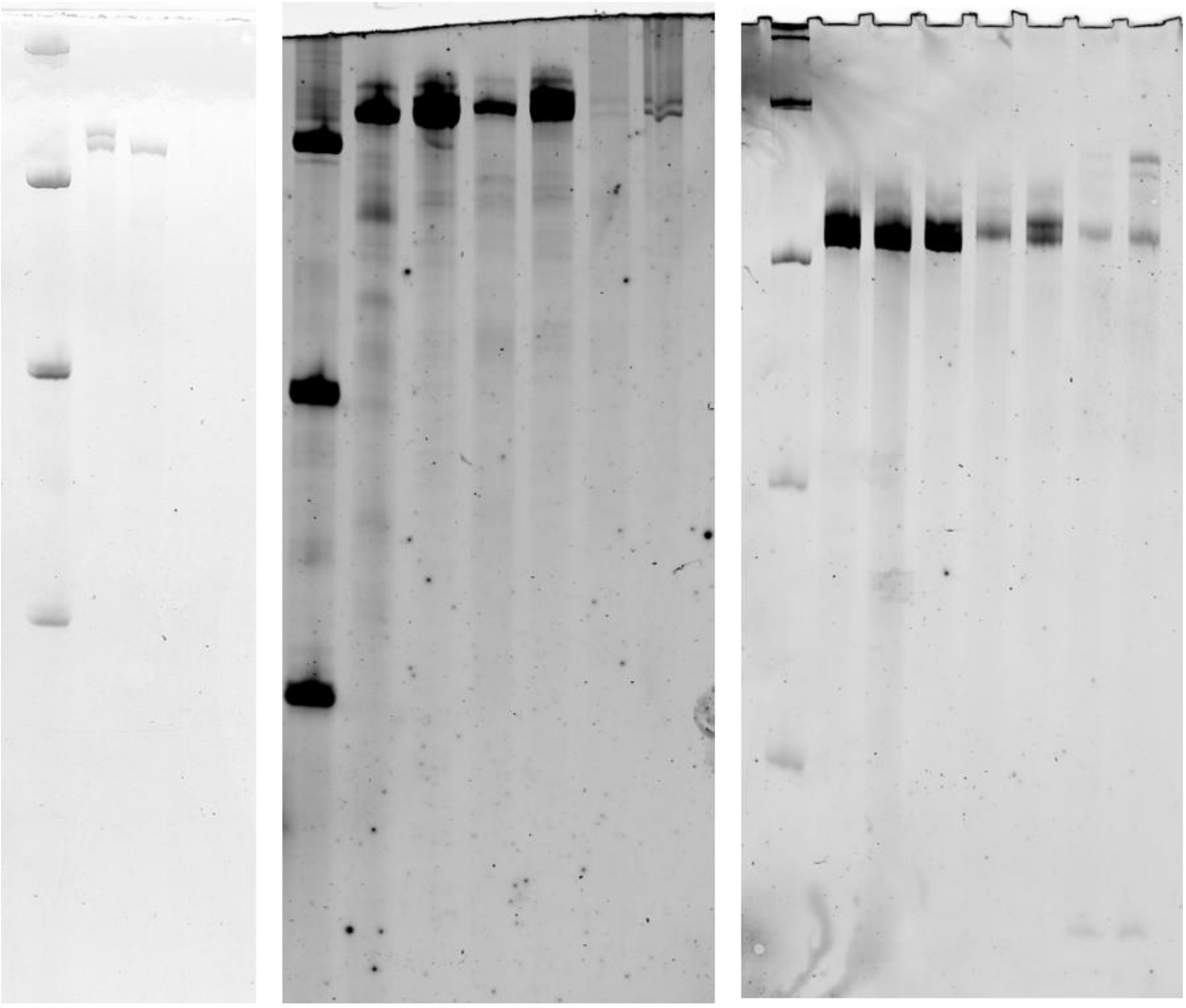

Figure S40: The original uncropped gel of Figure S12 – decapping of in vitro synthetized Ap$_2$A-U1 RNA by NUDT12.

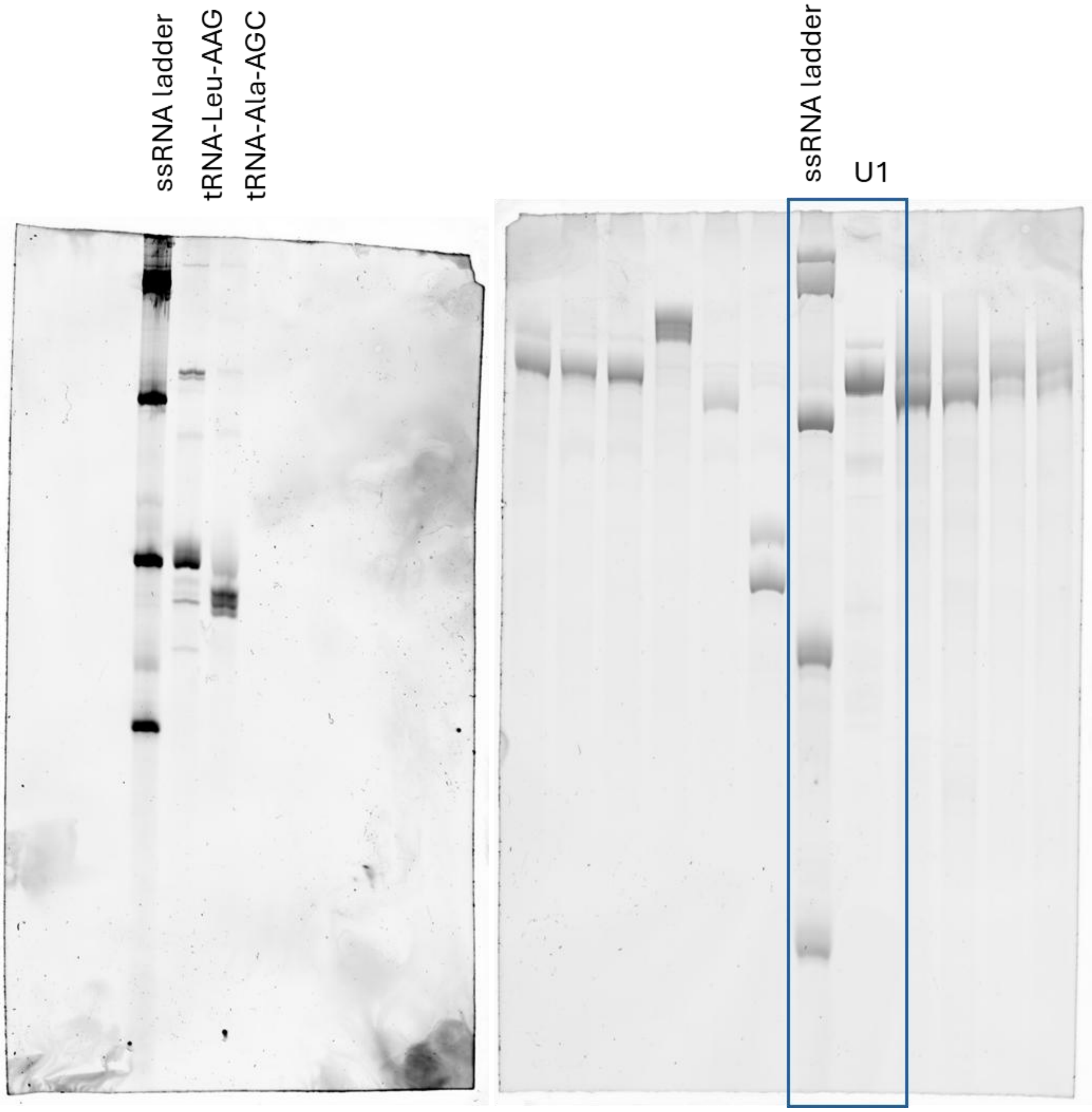

Figure S41: The original uncropped gel of Figure 4E (RNA pulldown of LeuAAG and AlaAGC tRNA and Figure S15 (RNU1-1 RNA pull-down)

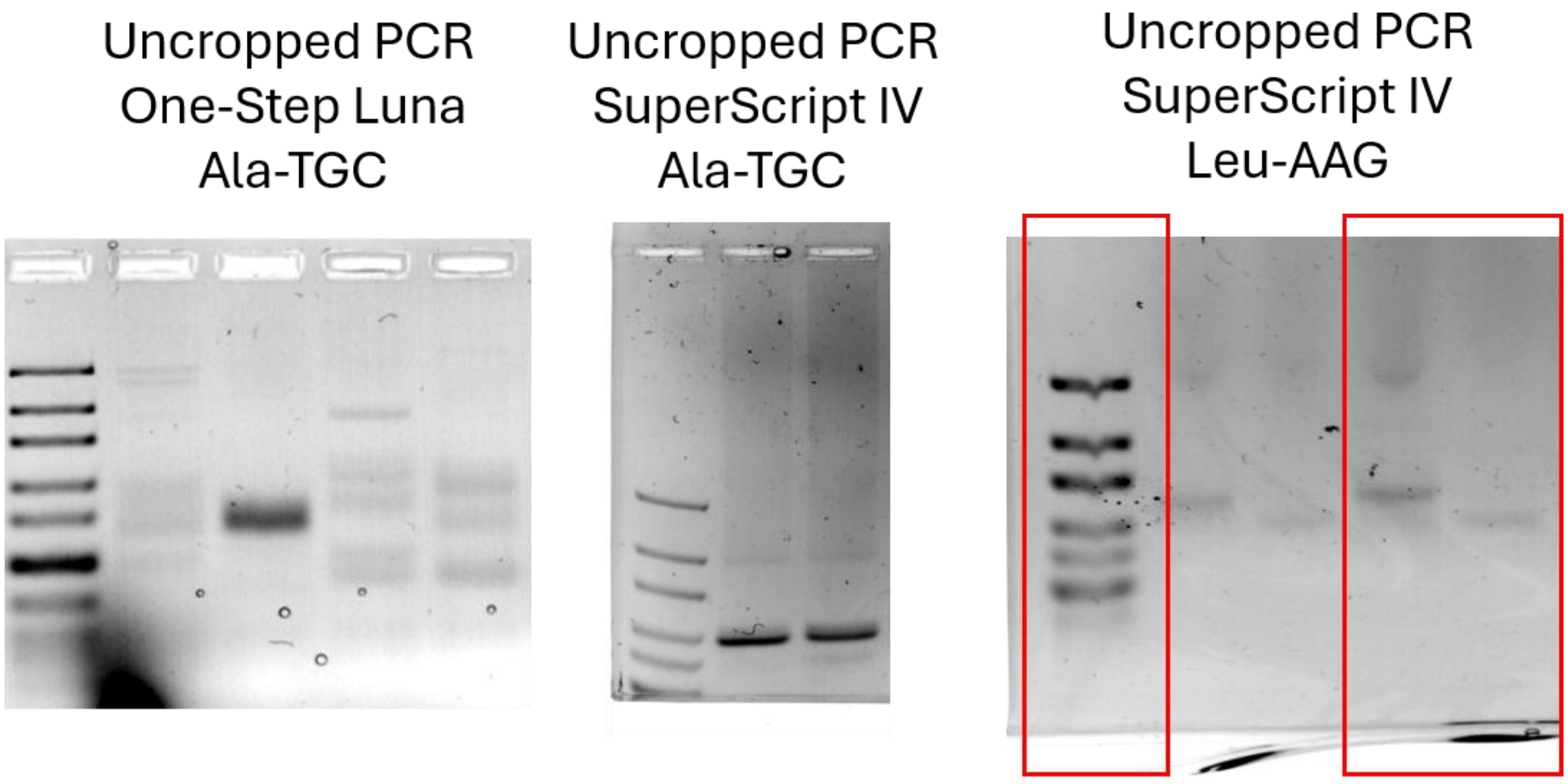

Figure S42: The original uncropped agarose gels of Figure S16 and S17 (PCR amplification of adaptor-ligated tRNA)

References

1. A. Kamali *et al.*, Enhanced Protein Precipitation with Ammonia Enables Rapid, Universal Extraction of Oligonucleotides for Bioanalysis. *ACS Omega* **10**, 46997-47006 (2025).
2. O. Hudeček *et al.*, Dinucleoside polyphosphates act as 5′-RNA caps in bacteria. *Nature Communications* **11**, 1052 (2020).
3. M. G. Song, S. Bail, M. Kiledjian, Multiple Nudix family proteins possess mRNA decapping activity. *Rna* **19**, 390-399 (2013).
4. J. Schindelin, C. T. Rueden, M. C. Hiner, K. W. Eliceiri, The ImageJ ecosystem: An open platform for biomedical image analysis. *Molecular Reproduction and Development* **82**, 518-529 (2015).
5. M. Leutert, R. A. Rodríguez-Mias, N. K. Fukuda, J. Villén, R2-P2 rapid-robotic phosphoproteomics enables multidimensional cell signaling studies. *Molecular Systems Biology* **15**, MSB199021 (2019).
6. R. Bruderer *et al.*, Extending the Limits of Quantitative Proteome Profiling with Data-Independent Acquisition and Application to Acetaminophen-Treated Three-Dimensional Liver Microtissues*[S]. *Molecular & Cellular Proteomics* **14**, 1400-1410 (2015).
7. S. Chen, Y. Zhou, Y. Chen, J. Gu, fastp: an ultra-fast all-in-one FASTQ preprocessor. *Bioinformatics* **34**, i884-i890 (2018).
8. M. Martin, Cutadapt removes adapter sequences from high-throughput sequencing reads. *EMBnet.journal* **17**, 3 (2011).
9. A. Hoffmann *et al.*, Accurate mapping of tRNA reads. *Bioinformatics* **34**, 1116-1124 (2018).
10. S. Hoffmann *et al.*, Fast mapping of short sequences with mismatches, insertions and deletions using index structures. *PLoS Comput Biol* **5**, e1000502 (2009).
11. P. Danecek *et al.*, Twelve years of SAMtools and BCFtools. *GigaScience* **10** (2021).
12. Y. Liao, G. K. Smyth, W. Shi, featureCounts: an efficient general purpose program for assigning sequence reads to genomic features. *Bioinformatics* **30**, 923-930 (2013).
13. H. Thorvaldsdóttir, J. T. Robinson, J. P. Mesirov, Integrative Genomics Viewer (IGV): high-performance genomics data visualization and exploration. *Briefings in Bioinformatics* **14**, 178-192 (2012).
14. H. Li, R. Durbin, Fast and accurate long-read alignment with Burrows–Wheeler transform. *Bioinformatics* **26**, 589-595 (2010).
15. A. Dobin *et al.*, STAR: ultrafast universal RNA-seq aligner. *Bioinformatics* **29**, 15-21 (2012).
16. M. C. Lucas *et al.*, Quantitative analysis of tRNA abundance and modifications by nanopore RNA sequencing. *Nature Biotechnology* **42**, 72-86 (2024).
17. A. G. Torres, O. Reina, C. Stephan-Otto Attolini, L. Ribas de Pouplana, Differential expression of human tRNA genes drives the abundance of tRNA-derived fragments. *Proceedings of the National Academy of Sciences* **116**, 8451-8456 (2019).
18. A. Andor *et al.*, TXNL1 has dual functions as a redox active thioredoxin-like protein as well as an ATP- and redox-independent chaperone. *Redox Biol* **67**, 102897 (2023).
19. J. M. Matés, J. A. Campos-Sandoval, J. de los Santos-Jiménez, J. Márquez, Glutaminases regulate glutathione and oxidative stress in cancer. *Archives of Toxicology* **94**, 2603-2623 (2020).
20. J. De los Santos-Jiménez, J. A. Campos-Sandoval, F. J. Alonso, J. Márquez, J. M. Matés, GLS and GLS2 Glutaminase Isoenzymes in the Antioxidant System of Cancer Cells. *Antioxidants* **13**, 745 (2024).
21. K. Klimešová, H. Petržílková, C. Bařinka, D. Staněk, SART3 associates with a post-splicing complex. *Journal of Cell Science* **136** (2023).
22. D. A. Basello *et al.*, Dynamic interaction of spliceosomal snRNPs with coilin explains Cajal body characteristics. *J Cell Biol* **224** (2025).
23. K. Klimešová *et al.*, TSSC4 is a component of U5 snRNP that promotes tri-snRNP formation. *Nature Communications* **12**, 3646 (2021).
